# Systematic integration of RNA-Seq statistical algorithms for accurate detection of differential gene expression patterns

**DOI:** 10.1093/nar/gku1273

**Published:** 2014-12-01

**Authors:** Panagiotis Moulos, Pantelis Hatzis

**Affiliations:** Biomedical Sciences Research Center ‘Alexander Fleming’, 34 Fleming str, 16672, Vari, Greece

## Abstract

RNA-Seq is gradually becoming the standard tool for transcriptomic expression studies in biological research. Although considerable progress has been recorded in the development of statistical algorithms for the detection of differentially expressed genes using RNA-Seq data, the list of detected genes can differ significantly between algorithms. We present a new method (PANDORA) that combines multiple algorithms toward a summarized result, more efficiently reflecting true experimental outcomes. This is achieved through the systematic combination of several analysis algorithms, by weighting their outcomes according to their performance with realistically simulated data sets generated from real data. Results supported by the analysis of both simulated and real data from different organisms as well as correlation with PolII occupancy demonstrate that PANDORA improves the detection of differential expression. It accomplishes this by optimizing the tradeoff between standard performance measurements, such as precision and sensitivity.

## INTRODUCTION

One of the common applications of RNA-Seq (1) is genome-wide transcript expression profiling and detection of differentially expressed genes (DEGs) across distinct biological conditions. RNA-Seq experiments, like all high-throughput techniques, are subject to several sources of bias, systematic or experimental (2). To account for such biases, several algorithms have been developed based on different statistical models (3). Another important factor contributing to the native complexity of RNA-Seq data is the often ambiguous interpretation of the observed results. For example, when two genes share functional elements (e.g. overlapping exons), it is challenging to distinguish the real source of expression. Such issues are more evident when trying to explain expression differences between different isoforms of the same gene. On the other hand, although RNA-Seq has inflated the number of potential applications of gene expression experiments, it is still often used as a replacement of DNA microarrays, because of its greater dynamic range, higher resolution and lower experimental noise.

A lot of research effort has been devoted either in developing novel methods for the detection of DEGs using RNA-Seq or in evaluating the performance of existing ones. It was recently shown that different methods perform best in different data sets or sources of error (4,5). This, however, results in significant variations in the quality of gene expression data published by different investigators, in addition to the inherent biological variations between the analyzed samples. Furthermore, different result reporting formats often lead to difficulties in the presentation and comprehension of the findings. A potential way to overcome the above caveats is to combine current algorithms toward the derivation of more robust gene lists, characterized by both higher statistical power and lower numbers of false positives and false negatives (referred to hereafter as ‘false hits’). In other words, instead of creating additional statistical algorithms, prior knowledge on the advantages and disadvantages of existing algorithms derived by extensive comparison studies (e.g. (5)) can be exploited. These algorithms can be combined based on their strength, by using, for example, a *P*-value weighting scheme (6), resulting in faster convergence to an optimized list of DEGs where the ratio between true positive genes and false hits is maximized.

In this article, we demonstrate that the systematic combination of several current RNA-Seq statistical algorithms based on performance weighting can improve the overall detection of DEGs, by reducing false hits while maintaining true positives. To this end, we developed PANDORA (**P**erform**AN**ce **D**riven sc**O**ring of **R**NA-Seq st**A**tistics), a new method for the analysis of RNA-Seq gene expression data which deploys a combination of existing algorithms. We show that PANDORA, as evaluated on simulated and real data sets, achieves significant improvements in both precision and sensitivity, and, in the worst case, similar performance to existing algorithms that have been previously evaluated as most robust (4,5). Finally, by correlating two independent methods for the evaluation of gene expression (RNA-Seq and PolII occupancy data), we show that PANDORA offers an intuitive methodology for the optimization of statistical accuracy and agreement between alternative transcriptional measurements. This method, together with additional statistical test combination methods, is implemented in metaseqR, an R/Bioconductor package. metaseqR provides a straightforward interface to several current statistical tests and normalization algorithms for RNA-Seq data. At the same time it provides greater insight to the data through extensive and comprehensive reports of the findings.

## MATERIALS AND METHODS

### *P*-value combination

metaseqR can combine the *P*-value scores returned by the application of more than one statistical tests with six approaches. Throughout the rest of this section, we generalize the term ‘*P*-value’ to include the score (1 – *posterior probability of differential expression*) returned by baySeq and the score (1 – *q statistic*) returned by NOISeq. Specifically, let *p_ij_* be the resulting *P*-value for gene *i* after the application of the statistical test *j*. Then, the combined *P*-value for gene *i*, is derived using one of the following approaches:

### Simes method

Let *p_i_*_1_, *p_i_*_2_, …, *p_im_* be the *P*-value scores returned for gene *i* after the application of *m* statistical tests. Let also *p_i_*_(1)_, *p_i_*_(2)_, …, *p_i_*_(_*_m_*_)_ be the aforementioned *P*-values sorted in increasing order. Then, according to Simes’ method, the probability

\begin{equation*} p_i^* = \mathop {\min }\limits_k \left\{ {p_{i(k)} /k} \right\},\;k \in (1, \ldots ,m) \end{equation*}

can be used as the combined *P*-value for a set of *m* statistical tests (7). In case of independent tests, this is the exact *P*-value for the combination. In case of dependency, numerical evidence (7) shows that this approximation can still be used.

### Fisher's method

According to the Fisher's method, let *f* be the statistic defined by the natural logarithm of the product of *m* individual *P*-values (from *m* statistical tests) multiplied by –2:

\begin{equation*} f_i = - 2\sum\limits_{j = 1}^m {\ln p_{ij} } \end{equation*}

It can be proved (8) that *f* follows an *X*^2^ distribution with 2*m* degrees of freedom, which can be used to derive the combined *P*-value from *m* statistical tests. However, Fisher's method was developed with the intention of combining *P*-values produced by the same statistical test applied in different data sets, where the underlying null hypothesis is the same (meta-analysis), rather than combining *P*-values produced by different statistical tests applied to the same data set. In the context of different statistical tests, the null hypothesis remains essentially the same (testing differential against non-differential gene expression), although there might be differences in its formulation, according to the context of each statistical test.

### Whitlock's method

According to Whitlock's weighted *Z*-method (9), the weighted *Z* statistic for each gene *i*

\begin{equation*} Z_j^w = \sum\limits_{j = 1}^m {{{w_j Z_j }/{\sqrt {\sum\limits_{j = 1}^m {w_j^2 } } }}} \end{equation*}

follows the standard Normal distribution *N*(0,1), which can be used to derive the *P*-value of the combined tests. Regarding the usage of the Whitlock method for combining *P*-values from different statistical tests, the assumptions described above (Fisher's method) also apply.

### Maximum *P*-value

In this case, the combined *P*-value is

\begin{equation*} p_i^* = \mathop {\max }\limits_j \left\{ {p_{ij} } \right\},\;j \in \left( {1,...m} \right) \end{equation*}

In the case of a predefined level of significance *α*, this approach is equivalent to the ‘intersection’ of the genes for which the null hypothesis of no differential expression is rejected (*p_ij_* < *α*), as these are derived from each statistical test. Thus, the final list will contain genes which have been characterized as differentially expressed by all the statistical tests applied. The maximum *P*-value ensures that the false positives are minimized at a (usually high) cost on the true positives (statistical power).

### Minimum *P*-value

In this case, the combined *P*-value is

\begin{equation*} p_i^* = \mathop {\min }\limits_j \left\{ {p_{ij} } \right\},\;j \in \left( {1,...,m} \right) \end{equation*}

In the case of a predefined level of significance *α*, this approach is equivalent to the ‘union’ of the genes for which the null hypothesis of no differential expression is rejected (*p_ij_* < *α*), as these are derived from each statistical test. Thus, the final list will contain genes which have been characterized as differentially expressed by at least one of the statistical tests applied. The minimum *P*-value ensures that the true positives are maximized at a (usually high) cost on the false positives (type I error).

### PANDORA *P*-value

In this case, the combined *P*-value is

\begin{equation*} p_i^* = \prod\limits_{j = 1}^m {p_{ij}^{w_j } } ,\;{\rm with}\;\sum\limits_{j = 1}^m {w_j = 1} \end{equation*}

where *w_j_* represent automatically assigned or user-specific weights for the *j* statistical tests performed. The weights can be automatically assigned according to the area under the false discovery curve (AUFC) estimated from simulated data based (possibly) on the data set under investigation, or can be user-defined according to previous performance experience (see ‘The choice of weights for PANDORA’ section in Supplementary Material). In the case of automatic estimation of weights using the AUFCs, the weights are estimated using the following formula:

\begin{equation*} w_j = \frac{{\sum\limits_{j = 1}^m {{{AUFC_j }/ {AUFC_j }}} }}{{\sum\limits_{j = 1}^m {\left( {\sum\limits_{j = 1}^m {{{AUFC_j }/ {AUFC_j }}} } \right)} }} \end{equation*}

where *AUFC_j_* is the area under the false discoveries curve for the results of statistical test *j*. Apart from the automatic weights assignment, metaseqR allows the user to specify weights, based. for example. on previous own experience, on previous studies or manual inspection of the performance measurements offered by metaseqR. In all cases, the supplied weights must have a unit sum.

### Precision-sensitivity tradeoff metrics

In order to quantify the tradeoff between precision and sensitivity when assessing methods, we use the *F*_1_-score (or *F*-measure). It comprises a statistical measurement used to assess the performance of statistical tests or binary classifiers (10) by combining both the precision and sensitivity. The *F*_1_-score is defined as the harmonic mean of precision and sensitivity:

\begin{equation*} F_1 = 2 \cdot \frac{{precision \cdot recall}}{{precision + recall}} \end{equation*}

If precision and sensitivity are replaced by their own definitions

\begin{equation*} precision = \frac{{TP}}{{TP + FP}},\quad recall = \frac{{TP}}{{TP + FN}}, \end{equation*}

respectively, it follows that:

\begin{equation*} F_1 = \frac{{2 \cdot TP}}{{2 \cdot TP + FP + FN}} \end{equation*}

As an additional precision-sensitivity tradeoff measurement, we use the *ad hoc* and more intuitive ratio of True Positives to the sum of False Positives and False Negatives, referred as *False Discovery Tradeoff* (*FDT*). The FDT is defined as:

\begin{equation*} FDT = \frac{{TP}}{{FP + FN}} \end{equation*}

### Performance evaluation tools

In order to assess the performance of the combined statistical testing of metaseqR as compared to the usage of single tests, we used several evaluation tools. Specifically:

- We used false discovery curves (FDCs) and false negative curves (FNCs) to measure the progression of type I and type II errors, respectively, while traversing lists ranked according to statistical significance from top-to-bottom for FDCs and from bottom-to-top for FNCs.
- We used receiver operating characteristic (ROC) analysis (the area under the curve) and assessment of the true false discoveries to demonstrate that weighting and combining statistical tests according to their performance yields results as good as or better than the best performing algorithms in the majority of test cases.
- We used the *F*_1_-score and the area under *F*_1_-score curves to demonstrate its maximization in final gene lists, produced by weighted *P*-value combination, in most simulated cases. The area under the *F*_1_-score curve is constructed by ranking the genes according to their combined *P*-value in increasing order and calculating the respective *F*_1_-score while traversing the ordered list.
- We applied ROC and *F*_1_-score analysis in real data from the SEQC project and true false discovery rate (FDR) analysis in a data subset from Brawand *et al*.
- We applied *F*_1_-score analysis in two RNA-Seq data sets (11,12), based on coupled PolII occupancy across gene bodies, the correlation of which with gene expression as measured by RNA-Seq was used as an independent measurement of transcription quantification.

All the aforementioned performance measurements regarding simulated data were calculated by averaging the results over 10 simulations.

### Simulated data

The synthetic data sets referenced in the Results section were generated with the make.sim.data.sd function included in metaseqR. This function implements the simulator described in (5), which creates synthetic RNA-Seq gene counts based on the negative binomial distribution, where the mean and dispersion parameters are estimated from real data. Additionally, as the main normalization algorithm that we use is EDASeq (13), which makes use of the GC content of genes, we assign to each synthetic gene a GC content value sampled from the respective model organism. For the purpose of this article, we used the following data sets (the Human, Mouse and Fruitfly data sets were downloaded from the ReCount database (14)):

- For the simulations based on human, we used combined data from Montgomery *et al*. (15) and Pickrell *et al*. (16) as also proposed in (5). The Montgomery data set contains gene expression abundances from 60 individual from European descent while the Pickrell data set contains gene expression abundances from 69 Nigerian individuals. The original purpose of both studies was to identify genetic variations.
- For the simulations based on chimpanzee, we used a subset of data generated by Brawand *et al*. (17) who studied gene expression evolution in 6 organs across 10 representative mammalian species. For the needs of the simulations we used a subset of four sequencing runs with libraries constructed from polyadenylated RNA extracted from the same number of male chimpanzee brain prefrontal cortexes.
- For the simulations based on mouse, we used data from Bottomly *et al*. (18). Among other work, the authors studied gene expression differences between two commonly used mouse models, C57BL/6J and DBA/2J. The data set contains RNA-Seq gene read counts from 21 samples, 10 C57BL/6J and 11 DBA/2J, respectively.
- For the simulations based on fruitfly, we used a subset of data generated by Graveley *et al*. (19) who studied *Drosophila melanogaster* transcriptome at various developmental stages. For the needs of the simulations we used a subset consisting of RNA-Seq read counts from 15 adult male fruitflies.
- For the simulations based on Arabidopsis thaliana, we used data from Yanming *et al*. (20), who studied the defense response of the plant against bacterial infection. The data set was embedded in the R package NBPSeq and the Bioconductor package TCC.

Based on the above data sets and the implemented simulator, we used two main synthetic data configurations from each organism:

- A simulated RNA-Seq data set with 10 000 genes, consisting of two experimental conditions, each with three replicates. The percentage of DEGs was set to 10% (1000 hypothetical genes), of which 50% are up-regulated in the first condition and 50% are up-regulated in the second.
- A simulated RNA-Seq data set with 10 000 genes, consisting of two experimental conditions, each with seven replicates. The percentage of DEGs was set to 12% (1200 hypothetical genes), of which ∼42% are up-regulated in the first condition and ∼58% are up-regulated in the second, comprising a slightly unbalanced set of deregulated genes among the two conditions.

### Real RNA-Seq data

In addition to the usage of simulated data, we evaluated the performance of metaseqR using data from the SEQC project, the latest development under the MAQC study, which historically has been used to assess performance, similarities and reproducibility between microarray platforms. The SEQC data set contains 92 spike-in RNA controls at known concentrations, which allow the construction of an *a priori* ground truth regarding the specific transcripts. Furthermore, the SEQC project includes a set of ∼1000 genes validated by TaqMan quantitative polymerase chain reaction (qPCR); thus the fold change of these genes as calculated by qPCR can be used to estimate true levels of differential expression and provide measurements regarding the performance of statistical algorithms and the combination methods of metaseqR. Details regarding the SEQC data set are discussed in detail elsewhere (4,21). For the purposes of this study we used processed SEQC data by Rapaport *et al*. (4), available at https://bitbucket.org/soccin/seqc/. In order to apply EDASeq normalization to SEQC data including the spike-in controls, we retrieved the nucleotide sequences of the spike-ins available from Life Technologies^TM^ and calculated their GC content. GC content for the remaining genes was retrieved from Ensembl (22).

Moreover, to assess the performance of metaseqR-supported algorithms and *P*-value combination methods regarding the control of false positive outcomes, we used the two conditions of the SEQC data individually, as well the data from Brawand *et al*. described in the previous section to perform ‘same versus same’ mock comparisons under settings where differential expression should not be detected between the mock biological conditions. For the SEQC data, we performed two comparisons using data from the individual groups of the data set (A and B) by separating each group to two subgroups (A1, A2 and B1, B2, respectively) with two replicates each. As both the groups contained five technical replicates, we excluded from each group the replicate which was clustered further from the other two in a sample-wise hierarchical clustering.

### RNA-Seq and PolII occupancy data

The performance of PANDORA was additionally assessed by correlating the fold change of DEGs derived from RNA-Seq data with the corresponding fold change in PolII occupancy across gene bodies. To this end we used the following two coupled (RNA-Seq and PolII occupancy) data sets: (i) RNA-Seq and PolII data from Mokry *et al*. (12), where a doxycyclin-inducible shRNA targeting β-catenin allows for complete and specific blocking of the constitutively active Wnt pathway in colorectal cancer cells and (ii) RNA-Seq and PolII data from Lin *et al*. (11) (GEO accession number GSE38148), where the RNA PolII elongation factor Ell3 is knocked-down in mouse ES cells. Genes were categorized as up- or down-regulated according to PolII occupancy if the logarithm (base 2) of fold change between average ChIP-Seq reads per 1 kb across the gene body in treatment over control was above the 90th or the 10th quantile of the fold change distribution, respectively.

## RESULTS

### PANDORA's approach and performance evaluation criteria

PANDORA is based on an intuitive approach for ranking each statistical test according to its performance based on real-time simulations. Synthetic data sets, with *a priori* known DEGs, are constructed using parameters estimated from well characterized real data sets for several organisms or from the investigator's own data. Consequently, the statistical analysis of these data sets can be used to assess the performance of each algorithm. The latter is assessed using FDCs, where the performance is measured by the number of false positive findings encountered in a list, ordered according to decreasing statistical significance. PANDORA uses the AUFC to construct weights for each algorithm which are applied to the *P*-values returned by each test.

We evaluated the performance of PANDORA using experimental settings based on simulated data or data acquired from the SEQC study (21) and Brawand *et al*. (17). The synthetic data sets were created using a previously described methodology (5,23) based on real publicly available RNA-Seq count data for five organisms, namely, *Homo sapiens* (Human), *Pan troglodytes* (Chimpanzee), *Mus musculus* (Mouse), *Drosophila melanogaster* (Fruitfly) and *Arabidopsis thaliana* (Arabidopsis). We used a limited set of simulations and real data, which are sufficient to prove the added value of PANDORA. Specifically, we restricted the properties of synthetic data sets to two main configurations: a data set with two conditions of three biological replicates each and balanced differential expression between conditions (‘3 replicates—balanced DEG’) and a data set with two conditions of seven biological replicates each and slightly unbalanced differential expression between conditions (‘7 replicates—unbalanced DEG’).

As shown below, the combined usage of statistical tests for RNA-Seq data does not necessarily perform better in terms of statistical power than certain algorithms reported to stand out in related evaluations. With certain evaluation metrics, this is something to be expected because of the convex *P*-value weighting scheme (Supplementary Material, Section 2.1). However, as performance is not only measured by power, but sensitivity as well, PANDORA offers an optimal tradeoff between precision and sensitivity, when multiple statistical tests are applied on the same data set and their resulting statistical scores are properly weighted. To demonstrate this, we used established measurements, such as ROC analysis and FDCs, as well as the *harmonic mean of precision and sensitivity* (*F*_1_-score, Materials and Methods). To avoid confusion, we will use the term ‘*P*-value’ to refer to all the statistical scores (*P*-values, posterior probabilities, etc.), even if not all statistical scores comprise nominal *P*-values.

### PANDORA performs similar to the best algorithms in detecting differential expression

We compared PANDORA with five additional combination methods, either intuitive (e.g. the intersection of all genes marked as statistically deregulated by each examined test) or mined from the related statistical literature and used in the appropriate context (e.g. the Simes *P*-value combination method). Specifically, we applied the following methods:

- The *P*-value combination method proposed by Simes (7), referred to henceforth as *Simes*.
- The union of all genes marked as statistically significant deregulated by each examined test by considering the minimum statistical score among the examined tests as the final *P*-value, referred to henceforth as *Union*.
- The intersection of all genes marked as statistically deregulated by each examined test by considering the maximum *P*-value among the examined tests as the final *P*-value, referred to henceforth as *Intersection*.
- The *P*-value combination method proposed by Fisher for the meta-analysis of multiple independent observations testing the same hypothesis (8), referred to henceforth as *Fisher*. Although the classical interpretation of meta-analysis refers to the application of the same statistical test in similar data sets to test against a total null hypothesis, we use it to combine the *P*-values of different statistical tests applied to the same data set under a generalized hypothesis test for each gene, which in our case is ‘differential’ against ‘non-differential’ expression.
- The meta-analysis procedure proposed by Whitlock (9), referred to henceforth as *Whitlock*. Its main advantage over the Fisher method is the possibility to use weights for each set of observations; we thus apply it as a counterpart to the PANDORA weighting scheme, using the same weights. We use it under the same assumptions as the Fisher method.

Moreover, we paralleled the performance of the combination methods to the performance of the six statistical tests (DESeq, edgeR's exact test, limma with the voom method, NBPSeq, NOISeq and baySeq (20,24–28)) when applied alone. In total, we compared 12 possible statistical analyses.

**Figure 1.**
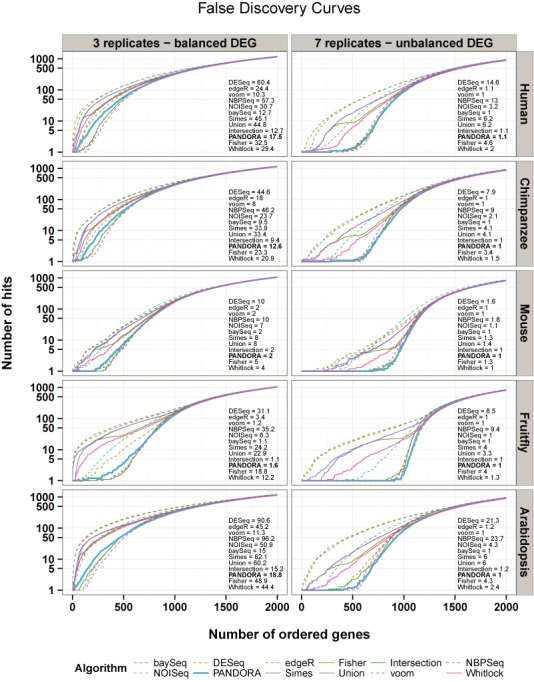
FDCs using EDASeq normalization. FDCs generated with simulated data for each statistical test and each *P*-value combination method for five organisms and two simulation configurations. The results for each organism and simulation configuration can be distinguished by the right and top side titles of each panel, referring to the organism from which simulation parameters are estimated and the simulation configuration, respectively. The performance value for each algorithm is displayed in each panel next to the curves and lower values depict best performing algorithms (Supplementary Methods). The lowest possible value is 1, indicating extremely few or no false discoveries among the first 500 top ranked genes according to statistical significance. Dashed lines represent individual tests, whereas solid lines represent *P*-value combinations and the thicker solid line highlights the FDC produced by PANDORA. As expected, the higher number of replicates has a definite impact in the accuracy and performance of all algorithms (right panels). The curves as well as the performance values are constructed and calculated, respectively, across 10 simulations for each organism and simulation configuration. The quantification of the AUFC for the PANDORA method is shown in bold.

Figure 1 depicts the FDCs for the six statistical tests alone and the six *P*-value combinations, applied on the synthetic data sets generated based on real data from five organisms. To avoid possible biases introduced by major (DESeq) or minor (edgeR, limma voom, NBPSeq, NOISeq and baySeq) differences in the normalization approaches followed by each algorithm, we used EDASeq (13) as a common normalization framework. An exploration of the ‘3 replicates—balanced DEG’ simulation (left panel), which resembles typical real-life experimental settings, reveals that the overall best performing individual test is limma voom, followed by baySeq and edgeR's exact test (edgeR's General Linear Models test was not examined here). DESeq and NBPSeq always share the last positions in terms of numbers of false positives located among the top ranked genes, whereas NOISeq is in the middle. Regarding the *P*-value combinations, classical meta-analysis (Fisher, Whitlock) and the Simes method, all perform quite poorly, presenting a large number of false positives among the top genes, very similar to the Union method which is the most liberal, and thus expected to return a large number of false discoveries. PANDORA performs quite well but not better than limma voom because of the convex nature of the weights. Notably, the Intersection, which is supposed to be the strictest selection strategy, appears inferior to limma voom and sometimes baySeq, apart from the Fruitfly simulations. This observation suggests that the often intuitive use of the common DEGs, returned by a variety of tests, as a robust signature to distinguish among biological conditions and sometimes as a ‘ground truth’ for evaluation (4), is not always an optimal solution, especially with a small number of replicates. The performance trends of individual as well as combined tests as assessed by FDCs do not change significantly when using normalization algorithms suggested in the distinct analysis packages (Supplementary Figure S4). They also do not change when combining adjusted for multiple testing instead of raw *P*-values for the four tests (DESeq, edgeR, limma and NBPSeq) returning nominal *P*-values (Supplementary Figures S13 and S15). Finally, we examined the progression of Type II errors using FNCs for each algorithm and simulation configuration. In this case, the above performance evaluation is almost reversed and many of the best performing algorithms exhibit a large number of false negatives, with certain combination methods (PANDORA among them) showing quite stable results (Supplementary Results).

### ROC analysis and assessment of FDRs place PANDORA among the top performing algorithms

**Figure 2.**
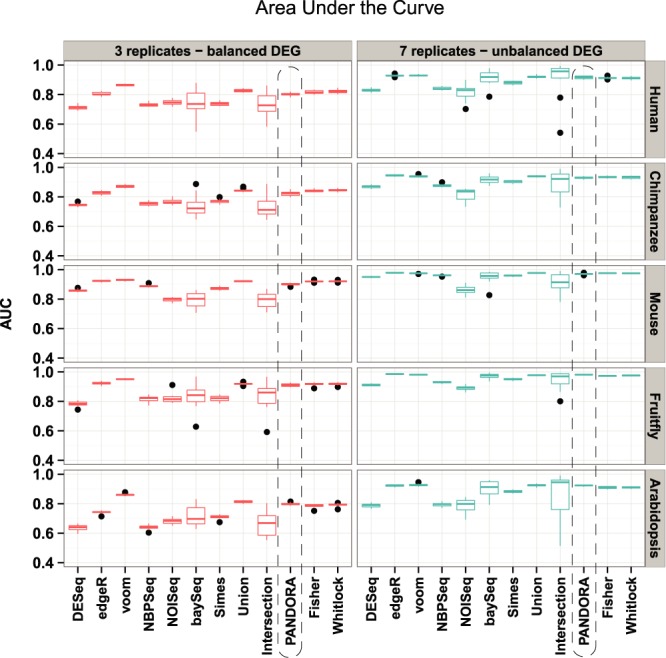
ROC analysis using EDASeq normalization. The boxplots depict the summarized areas under the ROC curve across 10 simulations for each organism and for each simulation configuration. The results for each organism and simulation configuration can be distinguished by the right and top side titles of each panel, referring to the organism from which simulation parameters are estimated and the simulation configuration, respectively. The dashed rectangle highlights the PANDORA method results. Each statistical test's and each *P*-value combination method's performance trends are similar, apart from the Mouse case, where the performance is increased in both simulation configurations. The impact of incorporating more replicates to the simulation is evident, as most methods achieve area under the curve values closer to 1 in all cases. PANDORA performs very similar to the highest scoring algorithms according to the area under the ROC curves.

Next, we evaluated the performance of PANDORA as compared to the other methods using ROC analysis and assessment of the true FDR, inspired by previous comparison work (5). ROC analysis using simulated data and the six individual statistical tests is in accordance with previous studies (5,26). Specifically, when using three replicates with balanced differential expression between two conditions (Figure 2, left panels), limma voom was constantly the best performer, followed by edgeR. The remaining four positions are shared among DESeq, NBPSeq, NOISeq and baySeq, with DESeq usually presenting lower performance and baySeq the most variant performance. The performance rank of the tests does not notably change when using a higher number of replicates and slightly unbalanced differential expression between conditions (Figure 2, right panels). Instead, the ROC area under the curve is significantly increased, as expected due to the larger number of replicates. The test that does not seem to benefit from more replicates is NOISeq. In addition, the performance variability of baySeq is reduced. Regarding the performance of the combination methods, Intersection is constantly the lowest scoring and most variable method when using a small number of replicates, followed by Simes. These observations do not particularly change in the presence of more replicates, apart from Intersection, which becomes more accurate, but equally variable. Finally, PANDORA performs well and very close to the highest performing individual tests in both simulation sets for all organisms, but not better, again due to the convex weighting scheme.

**Figure 3.**
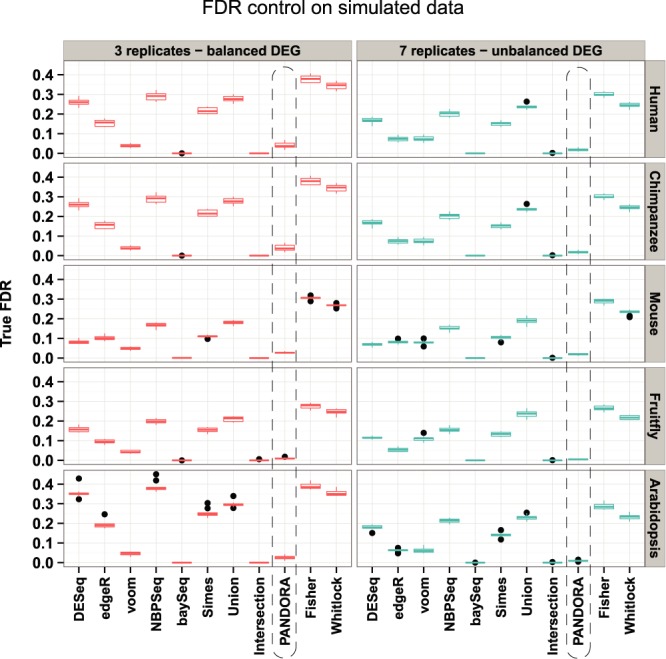
Analysis of true FDRs using EDASeq normalization. The boxplots summarize the true FDRs across 10 simulations for each organism and for each simulation configuration. The true FDRs for each organism and simulation configuration can be distinguished by the right and top side titles of each panel, referring to the organism from which simulation parameters are estimated and the simulation configuration, respectively. The true FDRs are estimated as the ratio of false positive hits determined by the *a priori* knowledge of DEGs for each simulation, to the respective number of genes passing a Benjamini–Hochberg FDR of 5%. The dashed rectangle highlights the PANDORA method results. From the individual statistical tests, NBPSeq constantly shows the highest true FDR, indicating poor performance regarding false discovery control and baySeq the lowest. From the *P*-value combination methods, Fisher and Whitlock show the highest true FDRs. PANDORA is the next best combination method to the Intersection (which is expected to have a good FDR because of stringency) in terms of FDR control.

We also assessed the accuracy of individual as well as combined tests by measuring their true FDR using simulated data (Figure 3). From this, we excluded NOISeq for the reasons explained in (5). Regarding the individual tests in the ‘3 replicates—balanced DEG’ simulations, only limma voom and baySeq show true FDR levels below 5%. DESeq and NBPSeq show the poorest performance, while edgeR is in the middle. These performance trends do not change for the ‘7 replicates—unbalanced DEG’ case, where the true FDR is closer to 5% for all individual tests. From the *P*-value combination methods, Intersection performs best, as expected due to stringency of selection, while the Simes method performance is between the performances of individual tests. Remarkably, the Union method, which is the most liberal and expected to exhibit the highest number of true false discoveries, performs better than the Fisher and Whitlock methods. Finally, and most importantly, PANDORA exhibits true FDR values below 5%, which is better than limma voom, the best individual algorithm so far according to our study. All the aforementioned observations do not change when using each package's specific normalization algorithm instead of EDASeq (Supplementary Figure S10).

### PANDORA exhibits the best tradeoff between precision and sensitivity

**Figure 4.**
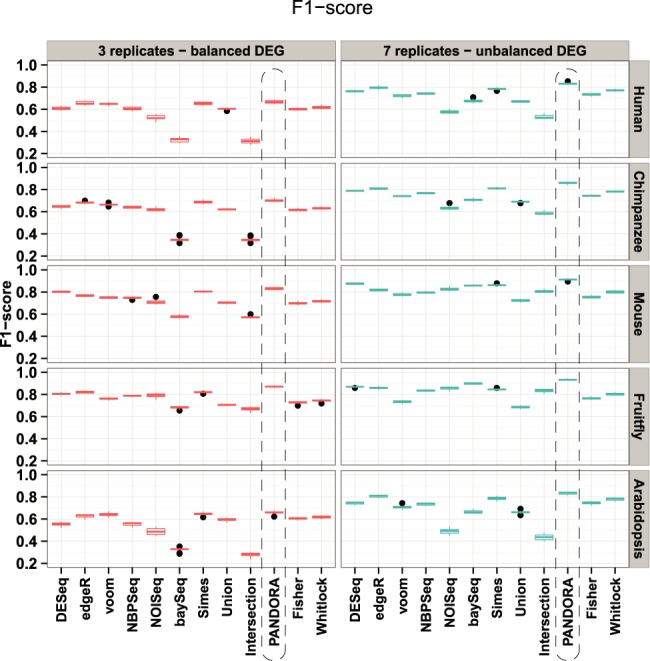
Analysis of the *F*_1_-score using EDASeq normalization. The boxplots summarize the *F*_1_-scores across 10 simulations for each organism and for each simulation configuration. The *F*_1_-scores for each organism and simulation configuration can be distinguished by the right and top side titles of each panel, referring to the organism from which simulation parameters are estimated and the simulation configuration, respectively. The dashed rectangle highlights the PANDORA method results. The prevalence of PANDORA is evident in all cases. Intersection and baySeq consistently show the lowest *F*_1_-score. *F*_1_-scores are calculated using the final gene lists returned by each method at a *P*-value cutoff of 0.05.

We evaluated the ability of individual as well as proposed combined tests to maximize the number of true positives, while at the same time keeping the number of false hits at a low level thus to maximize the *F*_1_-score. Figure 4 shows that in all simulated cases, PANDORA successfully combines several statistical tests toward a more robust gene list. Specifically, for the ‘3 replicates—balanced DEG’ configuration, PANDORA achieves *F*_1_-score levels slightly higher than the best performing individual tests for Human, Chimpanzee and Arabidopsis. The *F*_1_-score levels are much higher in Mouse and Fruitfly simulations. In the ‘7 replicates—unbalanced DEG’ study, the PANDORA method markedly stands out for all five organisms. Moreover, the good performance of PANDORA is supported by the area under the *F*_1_-score curve (Supplementary Figure S7), which depicts the evolution of the *F*_1_-score while traversing ranked gene lists from higher to lower statistical significance. Regarding the other combination methods, in the majority of cases they demonstrate lower *F*_1_-scores than PANDORA, but higher *F*_1_-scores than certain rather conservative individual tests (NOISeq, baySeq). Specifically, among the other methods, Simes presents the highest *F*_1_-score, followed by Whitlock, Fisher, Union and Intersection. Although Whitlock performs quite well, if we break down the *F*_1_-score value to its components (true and false hits, Supplementary Table S1 and Figure S12), we see that, although it takes into account the performance of each algorithm, the Whitlock method is poor because of the high number of false positives as compared to the gain in true positives. This suggest that the use of weighted *Z*-scores is not appropriate for combining statistical tests for RNA-Seq data and that PANDORA manages to accomplish this task in simulated data. Overall, our observations strengthen the main rationale behind PANDORA: as each statistical test presents certain advantages (whether these are low numbers of false positives at the cost of true positives or high numbers of true positives but also an excessive number of false positives too), a more robust gene list can be derived by applying an objective methodology to combine them. PANDORA achieves that by AUFC weighting, which ranks the statistical tests according to their ability to provide a reasonably low number of false positives in the top genes (500 in simulation studies). Finally, as expected, the Intersection method presents the lowest *F*_1_-score in all simulations across all organisms, being overly conservative and ignoring a lot of true hits returned by each statistical test (Supplementary Table S1 and Figure S12).

From the individual tests, limma voom and edgeR demonstrate the highest *F*_1_-score values, while baySeq and NOISeq show consistently lower values in most cases. This suggests that although the latter two are both successful in a satisfactory top-to-bottom statistical ranking of genes, their final outcome is excessively strict, resulting in significant loss of true positives (and a respective disproportional increase in false negatives, Supplementary Table S1 and Figure S12). Notably, baySeq presents an *F*_1_-score as low as the conservative strategy of constructing final gene lists by intersecting lists derived by individual tests. This is also supported by FNCs (Supplementary Figures S3, S5, S14 and S16), where in the majority of the cases baySeq presents a higher area under the FNC as compared to the other algorithms, either combined or individual. The tradeoff between precision and sensitivity is also assessed using corrected *P*-values (Supplementary Results) and by measuring the ratio of true positives to false hits (Supplementary Figures S25 and S26). Overall, the *F*_1_-score analysis reveals what more classical statistical algorithm performance evaluation measures, such as FDCs and ROC, do not: PANDORA successfully provides optimized gene lists, with respect to the tradeoff between true positives and false hits.

### Robust performance of PANDORA on real RNA-Seq data

Our evaluation results are based mostly on the simulated data sets, where we could control the outcome of the experiment. However, as data sets from real biological sources often exhibit unexpected biases and other sources of biological noise, we also evaluated PANDORA regarding (i) its ability to identify true or false differential expression with RNA-Seq data generated in the latest versions of the MAQC study, namely, the SEQC project for the evaluation of sequencing platforms regarding gene expression and (ii) its ability to control false discoveries with mock comparisons using data from SEQC and four male chimpanzee prefrontal cortex samples from Brawand *et al*. (17) which have also been used in the evaluation of the DEXSeq package (29). The SEQC study includes spike-in RNA controls at known concentrations (ERCC spike-in data), as well as a set of ∼1000 genes validated by TaqMan qPCR analysis (TaqMan data) which aid in testing the performance of DEG discovery.

**Figure 5.**
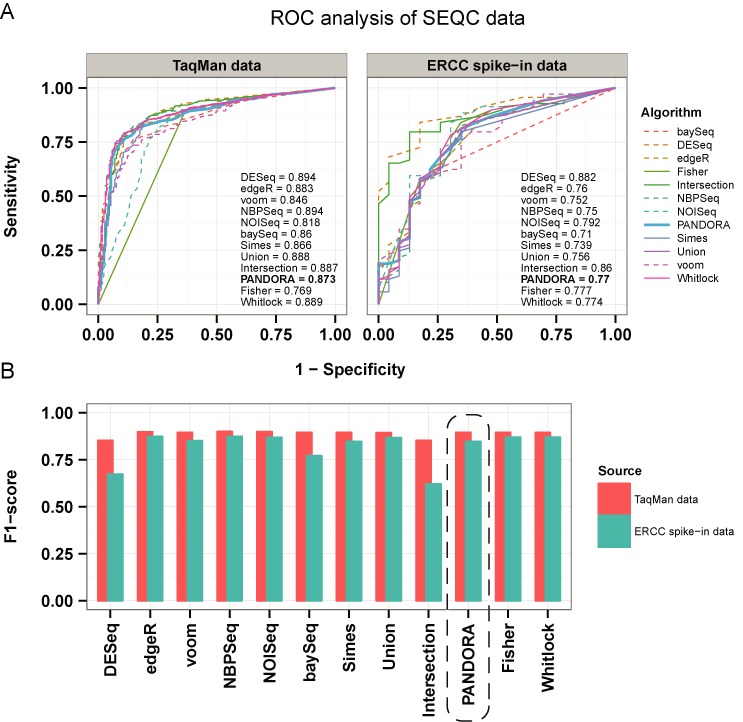
ROC and *F*_1_-score analysis for SEQC data using EDASeq normalization. (A) ROC curves calculated using TaqMan data (left panel) and ERCC spike-in data (right panel). Area under the curve values are shown on the right of each panel. Dashed lines represent individual tests, whereas solid lines represent *P*-value combinations and the thicker solid line highlights the ROC produced by PANDORA. In both cases DESeq performs slightly better than all the rest. In the case of TaqMan data, NBPSeq demonstrates the same performance as DESeq. The performance of PANDORA lies in the middle of area under the curve values in both cases, indicating adequate performance. See the main text for an explanation. (B) *F*_1_-scores using TaqMan data (red bars) and ERCC spike-in data (green bars). All methods apart from DESeq and Intersection achieve similar *F*_1_-scores when looking only at the TaqMan gene list (not the total putative DEG list of the SEQC data). The dashed rectangle highlights the PANDORA method results. When using ERCC spike-in data, baySeq and Simes also perform poorly. PANDORA is in the middle in both cases.

ROC analysis using the SEQC data classified PANDORA somewhere in the middle of the performances of the individual tests (Figure 5A). This was again to be expected because of the convexity of weights and because limma voom (which is weighted more than the rest) seems to underperform both in TaqMan and ERCC spike-in data. It should be noted that for the analysis of the SEQC data, we used weights estimated for Human from the combined data set of Montgomery *et al*. (15) and Pickrell *et al*. (16). We did not estimate weights based on the actual SEQC data, as the technical replication in the experimental design would bias the dispersion estimation in our simulator.

From the individual algorithms, DESeq and NBPSeq show the highest area under the curve, followed by edgeR, limma voom, baySeq and NOISeq when using the TaqMan data. When using ERCC spike-ins, DESeq is again top, followed by NOISeq, edgeR, limma voom and NBPSeq. Regarding the *P*-value combination methods, they all present similar values, apart from Fisher in TaqMan data and Simes in ERCC spike-in data. Union and Intersection score among the highest in both cases. The performance classification obtained by inspecting the *F*_1_-score values for each data set and for each algorithm (individual or combined, Figure 5B) is quite different. Thus, DESeq shows one of the lowest *F*_1_-scores, very close to the Intersection, which is expected to have a low *F*_1_-score for reasons explained in previous sections. All the other methods perform similarly when using TaqMan data, with NBPSeq and NOISeq slightly above the others. With ERCC spike-ins, edgeR, NBPSeq and NOISeq stand out, followed by limma voom. baySeq performs poorly and Fisher and Whitlock are the best *P*-value combination methods in this case. Again, the performance of PANDORA is in the middle, for the reasons explained above. The aforementioned observations do not change when using adjusted *P*-values (Supplementary Figure S23A and B), apart from the performance of DESeq regarding the *F*_1_-score, which drops even more for ERCC spike-in data.

Finally, we evaluated the approximation of the true FDR of individual algorithms and *P*-value combination methods using three ‘same versus same’ comparisons, two by separating the SEQC data groups into two subgroups each and one more using data from Brawand *et al*. (Table 1), excluding again NOISeq (5). All the tested methods achieve a ‘same versus same’ comparison FDR below 5% for all three comparisons. From the individual tests DESeq and NBPSeq score worst, presenting a higher FDR in two out of three comparisons. The best individual test is limma voom with FDR < 0.01% in all comparisons, followed by baySeq and edgeR. From the *P*-value combination methods, Intersection is the best, as expected (but with several pitfalls explained in previous sections), followed by PANDORA. The worst combination method is Fisher, followed by Whitlock, Union and Simes. Notably, PANDORA achieves one of the best rankings, presenting also several advantages, as described in previous sections. The aforementioned performance trend does not change when using each package's normalization algorithm instead of EDASeq (Supplementary Table S2).

**Table 1.** False discovery rates (Benjamini–Hochberg adjusted *P*-values) from three ‘same versus same’ comparisons (EDASeq normalization)

	DESeq	edgeR	voom	NBPSeq	baySeq	Simes	Union	Intersection	PANDORA	Fisher	Whitlock
SEQC group A	0.0238	0.0007	<0.0001	0.0177	0.0001	0.0201	0.0270	<0.0001	0.0007	0.0420	0.0266
SEQC group B	0.0003	0.0012	<0.0001	0.0019	<0.0001	0.0014	0.0033	<0.0001	<0.0001	0.0343	0.0101
Brawand data	0.0046	0.0009	<0.0001	0.0048	0.0001	0.0045	0.0065	<0.0001	<0.0001	0.0184	0.0133

### Optimal performance of PANDORA in correlating RNA-Seq with PolII occupancy

**Figure 6.**
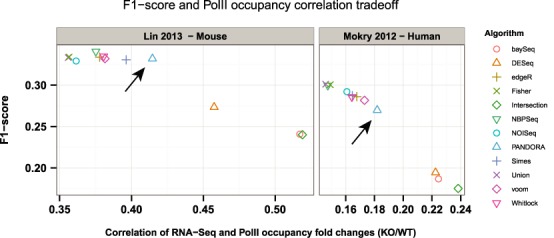
Tradeoff between accuracy (*F*_1_-scores) and correlation with PolII occupancy for all methods. When using two coupled RNA-Seq and PolII ChIP-Seq data sets, stricter algorithms (Intersection, baySeq) yield higher correlation with differences in transcription as measured by PolII occupancy at the cost of false hits (low *F*_1_-scores). The opposite is observed for more liberal methods (Union, edgeR). PANDORA achieves an optimal tradeoff between accuracy and correlation of transcription differences between treatment and control, as measured by RNA-Seq and PolII occupancy. The arrows point at the PANDORA method.

Next, we sought to evaluate PANDORA's performance on real RNA-Seq data by utilizing coupled PolII occupancy across gene bodies as an alternative approach for transcription quantification. The data sets we utilized included coupled RNA-Seq and PolII ChIP-Seq data generated, in one instance, in a colorectal cancer cell line engineered to carry a doxycyclin-inducible shRNA targeting β-catenin, which allows for complete and specific blocking of the—in these cells constitutively active—Wnt pathway (12); in the other instance in mouse ES cells after shRNA-mediated knock-down of the RNA PolII elongation factor Ell3 (11). Both coupled data sets utilized consider knock-down studies of factors that are expected, among others, to change PolII occupancy over gene bodies as well. Expression changes were calculated by RNA-Seq and PolII occupancy knock-down over wild type. *F*_1_-scores for each statistical algorithm and *P*-value combination method were calculated by using PolII occupancy as a ‘ground truth’ representation. To evaluate the tradeoff between the correlations of transcription changes, the above were deployed as a graphical evaluation method of each algorithm (Figure 6). As expected, stricter algorithms which yield fewer DEGs (Intersection, baySeq, DESeq) show better correlation with PolII occupancy but much lower *F*_1_-scores, because of the exclusion of many true hits, as those are defined by a cutoff in PolII occupancy across gene bodies. On the other hand, more liberal algorithms (Union, NBPSeq, Fisher, edgeR) show a larger *F*_1_-score but a much lower correlation with PolII occupancy. It is evident that PANDORA achieves an optimal tradeoff between precision-sensitivity (*F*_1_-score) as defined by PolII occupancy and correlation of gene expression as measured by two independent techniques. The optimization is clearer for the Lin *et al*. data set (Figure 6, left panel). This observation highlights the added value of PANDORA regarding both the optimization of the tradeoff between true and false hits, as well as the fact that it yields biologically significant results: i.e. good correlation between two gene expression abundance methods using real data, when the number of DEGs is not known *a priori*.

## DISCUSSION

In this article, we present PANDORA, a method which combines existing statistical tests for RNA-Seq data. PANDORA is implemented in metaseqR, a Bioconductor package, which offers an interface for several RNA-Seq data normalization methods and statistical tests coupled with extensive and comprehensive reports of the results. The strong points of PANDORA and metaseqR not fully addressed by existing packages are:

- The optimization of the tradeoff between precision and sensitivity in the vast majority of test cases.
- The robustness in detecting differential expression without loss of statistical power.
- The provision of a straightforward interface to eight normalization methods and nine statistical tests for RNA-Seq gene expression data through the use of seven R/Bioconductor packages developed for this purpose, allowing any combination among the above.
- The detailed, comprehensive and interactive report produced at the end of each analysis, which to our knowledge is unique among related open-source tools.
- The intuitive combination of multiple statistical tests applied on the same data with six possible methods.

RNA-Seq is gradually becoming the standard tool in gene expression research, with a wealth of statistical algorithms for the detection of DEGs emerging recently. Although there is a recent shift in software tool development aimed at understanding more complex genomic events that can be detected and studied by RNA-Seq (e.g. alternative splicing), it is most frequently applied to investigate changes in a ‘summarized gene’ expression across several experimental conditions. In addition, looking into more complex events, such as alternative isoform representation, often benefits from having established robust calls for deregulated genes. This is partly justified by a recent publication (30) where the authors analyzed transcript expression across several human tissues and cell lines and concluded that there is one dominant transcript per gene.

Our results were described based on raw, rather than corrected for multiple testing, *P*-values where appropriate. The issue of multiple testing correction and its application has been exhaustively treated in the literature. Its effect can be quite misleading with certain algorithms (e.g. DESeq, NBPSeq). Specifically, using simulated data sets for five organisms and two experimental configurations, the performance of DESeq, edgeR, NBPSeq and limma voom is not improved when using adjusted *P*-values, as measured by FDCs (Supplementary Figures S13 and S15). On the other hand, when using adjusted *P*-values for these four statistical tests, the performance as measured by FNCs dramatically drops, with limma voom exhibiting the most moderate accuracy loss. We thus believe that such results/phenomena are usually not noted, as most efforts in statistical algorithms are directed toward the minimization of false positives—without regard to the false negatives. This leads to overly strict outcomes and restricted downstream analyses (e.g. pathway analysis).

One possible solution to this problem would be to use either Bayesian approaches (e.g. baySeq or EBSeq) or more empirical ones (e.g. NOISeq), where ‘traditional’ multiple testing correction is not applied. We demonstrate that although these methods perform quite well as assessed by FDCs, FNCs and ROC analysis, when looking at actual numbers of true and false hits at specific statistical thresholds, NOISeq and baySeq perform badly: they generate an excessively high number of false hits, particularly false negatives. For the researcher who wants to look at a strict list of top DEGs, this is welcome. It, however, can also be achieved by imposing stricter statistical thresholds with other algorithms. In addition, not all methods return nominal *P*-values which can be subjected to multiple testing correction (NOISeq, baySeq) and, for these reasons, raw *P*-values have been used before for evaluation purposes (5). Using *P*-value combination applied on many statistical tests with unadjusted *P*-values seems to provide a solution to the problem described above. Specifically, PANDORA achieves the goal of a good tradeoff between true and false hits, by combining statistical tests using weights based on FDCs. Most importantly, it performs well when assessing accuracies based on real data. Thus, we propose that PANDORA can be used as an empirical alternative to the often strict multiple testing correction procedures.

As shown by *F*_1_-score analysis, PANDORA is not the best performer when combining *P*-values adjusted for multiple testing correction. This, in our opinion is not a bottleneck, because, first, in a realistic experimental setting, the inclusion of more than three biological replicates is rare. Our analysis indicates that in the presence of a low number of replicates per experimental condition, and when using adjusted *P*-values for DESeq, edgeR, limma voom and NBPSeq: (i) the restriction of false positives is rather low as demonstrated by FDCs, (ii) the loss in numbers of false negatives is high as demonstrated by FNCs, (iii) ROC analysis does not show notable differences in performance and (iv) *F*_1_-scores slightly drop in most cases for the four tests, especially in the human and mouse cases, two organisms that dominate current biological studies. Second, in some cases a PANDORA type of combining tests may not be desirable. This would occur either because it does not perform well for a specific data set (metaseqR provides evaluation facilities to test this) or a specific statistical test known to work well for a biological system under investigation. In such cases, metaseqR still provides an easy interface to several normalization algorithms, statistical tests and quality control measures, combined with very rich and detailed reporting capabilities.

In simulation studies, where the synthetic data sets are produced by strictly controlled computational means, PANDORA works considerably well. This may not always be the case with real data. The analysis of real RNA-Seq data from the SEQC project demonstrated a more moderate performance of the PANDORA method. At this point, it should be stressed that (i) we used Human weights estimated from the Montgomery–Pickrell combined data sets, which give greater weights to limma voom, edgeR and baySeq algorithms and (ii) the assessment is based on the TaqMan data and the ERCC spike-in controls, which both constitute a fixed set of ‘ground truth’ transcripts and the performance is based on these fixed numbers. In a real-life situation, there is no fixed number of DEGs that are expected from an experiment. This number is rather defined by statistical and/or empirical thresholds chosen by the analyst, without any guarantee that the false positives and false negatives are minimized. In this sense, methodology assessment with simulated data sets, where the number of differential synthetic genes is known but each test is free to return any number of genes, may well prove more crucial in the determination of which methods perform best.

Furthermore, technical replication in the SEQC data set is suitable for assessing technology reproducibility. However, it fails to capture biological noise present in more realistic experimental settings, which can be partially caught by simulated data created with parameters estimated from real data. We thus advocate the use of PANDORA, which excels in the majority of test cases, as its usage can offer a very good starting point for downstream analyses. The gene lists with minimized false hits it generates should be appreciated especially in low replication data sets, such as clinical applications.

The robustness of PANDORA is further supported when using PolII occupancy as a measurement of transcription. When the correlation of differential gene expression, as detected by RNA-seq, with differential average PolII occupancy across gene bodies was used as an independent accuracy measurement, we observed that PANDORA offers a good tradeoff between precision and sensitivity (*F*_1_-score). At the same time it achieves a good correlation with PolII occupancy. On the other hand, very liberal methods (e.g. Union, NBPSeq) fail to correlate well with PolII, even if they generate higher *F*_1_-scores. This is the expected outcome of the inclusion of more false positives. Stricter methods correlate better with PolII (again to be expected because of stringency), but fail to offer a good tradeoff between precision and sensitivity.

Finally, at the end of each analysis, metaseqR creates a very detailed and partially interactive report, not offered by similar open-source packages. This report includes, among others, an automatically generated text which can be directly used in the ‘Methods’ section of a scientific article and aids the bench biologist in understanding the pre-processing, normalization, data filtering and statistical testing that has been performed. Detailed information regarding the numbers of DEGs and the reproducibility of the analysis is also provided. In addition, a lot of diagnostic plots with short explanatory texts are available, coupled with tables depicting the top DEGs and all the intermediately generated read count tables can be retrieved through the report for further analysis. In this way, the experimentalist has a quick overview of the results without traversing multiple and separated gene lists and figures.

To conclude, PANDORA is a new method for weighting the results of statistical tests for RNA-Seq data based on their performance. Using simulated data generated based on parameter estimation from real data sets we have demonstrated that PANDORA achieves optimal tradeoff between precision and sensitivity. Finally, we have shown that PANDORA performs as well as individual tests using classical evaluation measurements and that it achieves an optimal tradeoff between performance and correlation of differential expression as measured by PolII occupancy.

## AVAILABILITY

PANDORA is implemented in metaseqR, a Bioconductor (http://www.bioconductor.org) package for the analysis of RNA-Seq gene expression data providing an interface for several normalization methods and statistical tests, methods for combining statistical tests as well as detailed and comprehensive reporting facilities.

## SUPPLEMENTARY DATA

Supplementary Data are available at NAR Online.

SUPPLEMENTARY DATA
