# Supplementary material for: Systematic integration of RNA-Seq statistical algorithms for accurate detection of differential gene expression patterns
Source: Nucleic Acids Res. 2014 Dec 1;43(4):e25. doi: 10.1093/nar/gku1273 (PMC4344485; doi:10.1093/nar/gku1273)
Supplement: SUPPLEMENTARY DATA [file supp_43_4_e25__index.html]

Systematic integration of RNA-Seq statistical algorithms for accurate detection of differential gene expression patterns — Systematic integration of RNA-Seq statistical algorithms for accurate detection of differential gene expression patterns — SUPPLEMENTARY DATA 

# Systematic integration of RNA-Seq statistical algorithms for accurate detection of differential gene expression patterns

## SUPPLEMENTARY DATA

**Files in this Data Supplement:**

- SUPPLEMENTARY DATA
