# Supplementary material for: Systematic integration of RNA-Seq statistical algorithms for accurate detection of differential gene expression patterns
Source: Nucleic Acids Res. 2014 Dec 1;43(4):e25. doi: 10.1093/nar/gku1273 (PMC4344485; doi:10.1093/nar/gku1273)
Supplement: SUPPLEMENTARY DATA [file supp_gku1273_nar-02511-met-n-2014-File009.pdf]

Systematic integration of RNA-Seq statistical algorithms for accurate detection of differential gene expression patterns

Panagiotis Moulos, Pantelis Hatzis

Biomedical Sciences Research Center ‘Alexander Fleming’, 34 Fleming str, 16672, Vari, Greece.

Supplementary Material

Contents

Contents ... 2
1. Supplementary results ... 4
1.1. metaseqR package overview ... 4
1.2. General performance evaluation using the progression of Type II errors (FNCs) ... 4
1.3. ROC analysis and assessment of FDR with package specific normalization and corrected p-values ... 5
1.4. Tradeoff between precision and sensitivity when using corrected p-values ... 6
1.5. ROC and F_1-score analysis on SEQC data using normalization algorithms specific to each statistical algorithm ... 6
2. Supplementary methods ... 8
2.1. The choice of weights for PANDORA ... 8
2.2. Quantitation of FDCs and FNCs ... 9
2.3. Data input ... 9
2.4. Data filtering ... 11
2.4.1. Exon filters ... 11
2.4.2. Gene filters ... 12
2.5. Data normalization ... 13
2.6. Statistical testing ... 14
2.7. p-value combination ... 14
2.8. Diagnostic plots ... 17
2.9. Data export and reporting ... 23
3. Supplementary figures ... 25
Figure S1: The analysis workflow of metaseqR ... 25
Figure S2: False Discovery Curves for six statistical tests using EDASeq normalization ... 26
Figure S3: False Negative Curves using EDASeq normalization and unadjusted p-values ... 28
Figure S4: False Discovery Curves using each package's specific normalization and unadjusted p-values ... 30
Figure S5: False Negative Curves using each package's specific normalization and unadjusted p-values ... 32
Figure S6: Receiver Operating Characteristic analysis using each package's specific normalization and unadjusted p-values ... 34
Figure S7: Areas under the F_1-score curves using EDASeq normalization and unadjusted p-values . 36

Figure S8: Analysis of the F_1-score using each package's specific normalization and unadjusted p-values ... 39
Figure S9: Areas under normalized F_1-score curves using each package's specific normalization and unadjusted p-values ... 41
Figure S10: Analysis of true False Discovery Rates using each package's specific normalization ... 43
Figure S11: Receiver Operating Characteristic and F_1-score analysis for SEQC data using each package's specific normalization and unadjusted p-values ... 45
Figure S12: False Negative, False Positive and True positive hits for all simulated data ... 47
Figure S13: False Discovery Curves using EDASeq normalization and adjusted p-values ... 49
Figure S14: False Negative Curves using EDASeq normalization and adjusted p-values ... 51
Figure S15: False Discovery Curves using each package's specific normalization and adjusted p-values ... 53
Figure S16: False Negative Curves using each package's specific normalization and adjusted p-values ... 55
Figure S17: Receiver Operating Characteristic analysis using EDASeq normalization and adjusted p-values ... 57
Figure S18: Receiver Operating Characteristic analysis using each package's specific normalization and adjusted p-values ... 59
Figure S19: Analysis of the F_1-score using EDASeq normalization and adjusted p-values ... 61
Figure S20: Analysis of the F_1-score using each package's specific normalization and adjusted p-values ... 63
Figure S21: Areas under the F_1-score curves using EDASeq normalization and adjusted p-values ... 65
Figure S22: Areas under the F_1-score curves using each package's specific normalization and adjusted p-values ... 67
Figure S23: Receiver Operating Characteristic and F_1-score analysis for SEQC data using EDASeq normalization and adjusted p-values ... 69
Figure S24: Receiver Operating Characteristic and F_1-score analysis for SEQC data using each package's specific normalization and adjusted p-values ... 71
Figure S25: Analysis of the False Discovery Tradeoff using EDASeq normalization and unadjusted p-values ... 73
Figure S26: Analysis of the False Discovery Tradeoff using each package's specific normalization and unadjusted p-values ... 75
4. Supplementary tables ... 76
5. References ... 79

1. Supplementary results

1.1. metaseqR package overview

We developed metaseqR, a gene expression analysis pipeline for RNA-Seq data, starting from raw mapped reads in SAM/BAM/BED format or a table of read counts for each gene, and resulting in a comprehensive report including lists of candidate differentially expressed genes accompanied with diagnostic plots and associated statistics. The workflow implemented in metaseqR is presented in Figure S1 and it consists of seven main steps: i) data import, ii) annotation retrieval (gene models or exons), iii) data normalization, iv) data filtering, v) statistical analysis with one or more of the supported statistical tests, vi) when applying more than one statistical test, p-value combination to produce a summarized statistical score and vii) data export and report generation. metaseqR is implemented as an R package and is freely available from the Bioconductor repository (1). It provides an interface to several normalization algorithms implemented as Bioconductor packages (EDASeq, DESeq, edgeR, NBPSeq, NOISeq) and several widely used statistical tests developed for RNA-Seq data (DESeq, edgeR, limma voom, NBPSeq, NOISeq and baySeq). It additionally supports a variety of quality control and data exploration plots, parts of which are adopted from the aforementioned packages, while the rest are metaseqR-specific. Further details are presented in Supplementary methods. Analytical usage details and explanation of various pipeline arguments can be found in the package documentation.

1.2. General performance evaluation using the progression of Type II errors (FNCs)

The performance evaluation for each of the presented algorithms (individual and combined) presented in the main text is almost reversed when looking at the progression of type II error both for EDASeq normalized data (Figure S3) as well as when using normalization specific to each package (Figure S5) and adjusted p-values, where applicable (Figures S14, S16). Specifically, limma voom and baySeq exhibit extended areas under the false negative curve indicating large numbers of false negatives. baySeq performs worse in terms of type II error assessment in three out of five organism simulations (Chimpanzee, Mouse and Fruitfly) while limma voom performs worse in the other two. In addition, baySeq does not seem to benefit from the presence of more replicates ('7 replicates – unbalanced DEG' simulation) like the rest of the tests. The above are confirmed by inspecting the number of false negatives in Table S1 where limma and baySeq show high numbers of missed true cases as compared to the rest of the individual tests. Regarding the combination methods, the Union performs best, as expected, as using the smallest p-value ensures the minimization of false negatives at the cost of type I error. Intersection is among the worst methods, as expected, but, interestingly, not the worst, as in most

cases, either limma voom or baySeq performance is poorer. Finally, Simes, Fisher, Whitlock and PANDORA appear quite stable, changing performance positions according to simulation configuration and organism. When normalizing the data using the suggested normalization method of each package (Figure S5), there is a small improvement in the performance of most algorithms, apart from NOISeq in all simulations and baySeq in most. At this point it should be noted that, apart from DESeq and NOISeq, all other packages use as default normalization the Trimmed Mean of M-values normalization (TMM) (2), usually as this is implemented in edgeR or slight alternatives. The default normalization method in NOISeq is RPKM normalization, but we changed it to TMM because of reported poor performance of RPKM ((3) and therein). This change only improved the performance of NOISeq.

Finally, when using adjusted instead of raw p-values for DESeq, edgeR, limma voom and NBPSeq (Figures S14, S16), we observed an explosion in the number of false negatives for these methods, with limma exhibiting the smallest increase. This explosion affected mostly the Simes, Intersection and Whitlock, and less the Union, PANDORA and Fisher methods. Notably, among the less affected methods is PANDORA. Overall, our observations based on the analysis of FDCs and FNCs with simulated data suggest that p-value combination methods do not always improve the performance of individual tests. Nevertheless, when used they perform better both in terms of type I and type II error, than tests exhibiting poor performance according to the aforementioned methods, without significantly affecting the performance of the best single tests. In particular, when using raw p-value for the four statistical tests returning nominal p-values (DESeq, edger, limma voom and NBPSeq), the Simes and PANDORA methods are the most stable ones. When using adjusted p-values for the aforementioned tests and combining afterwards, Fisher and PANDORA seem to maintain quite stable areas under the FDCs.

1.3. ROC analysis and assessment of FDR with package specific normalization and corrected p-values

In the main text, we describe the performance of individual as well as p-value combination methods using raw p-values and the common EDASeq normalization framework (subsection “ROC analysis and assessment of False Discovery Rates places PANDORA among the top performing algorithms” in the “Results” section). These performance trends do not show extreme alterations when using normalization methods bundled with each package (Figure S6) orusing adjusted p-values for DESeq, edgeR, limma voom and NBPSeq (Figures S17, S18). The only notable change in the former case is that some of the individual tests perform slightly better than with the common EDASeq normalization, while, in the latter case, the performance of the four tests with adjusted p-values drops (with limma

voom and edgeR presenting moderate losses) causing a subsequent drop in the weighted combination methods.

1.4. Tradeoff between precision and sensitivity when using corrected p-values

In the main text, we describe the precision and recall tradeoff which is accomplished by the application of each individual statistical test as well as the p-value combination methods and show that PANDORA achieves an optimal tradeoff between precision and recall as measured by the F_1-score. This assessment is performed using raw p-values. When combining corrected p-values for the four algorithms for which this can be applied, the results are quite different regarding the F_1-score assessment, as compared with the uncorrected case (Figure S19). Specifically, the F_1-score in some cases (Human, Chimpanzee and Arabidopsis in the '3 replicates – balanced DEG' simulations) slightly drops. This drop seems to have an effect on the PANDORA method, whose performance also either drops or remains at the similar levels. In other cases (Mouse and Fruitfly in the '3 replicates – balanced DEG' simulations), F_1-score is increased for all apart from DESeq, and especially increased for edgeR (expected if we refer to the p-value histograms in the supplementary material of (4)). PANDORA is no longer the best performing method in terms of absolute comparisons in the simulations with three replicates. However, its F_1-score values remain stable as compared with the F_1-score analysis using unadjusted p-values. Simes, Union and Intersection methods behave similarly to the unadjusted p-values case and the performance of Fisher as well Whitlock is improved. The F_1-score for all individual tests and combination methods is boosted when using more replicates ('7 replicates – unbalanced DEG' configuration). edgeR alone seems to perform very well in all cases and the performance of PANDORA is restored in most, especially in the Mouse case, where it shows the highest overall F_1-score. Finally, although p-value adjustment renders DESeq, edgeR, limma voom and NBPSeq stricter, they still perform better in terms of F_1-score than NOISeq and baySeq. These observations are not altered when using each package's specific normalization method (Figure S20) and the results are similar when inspecting the progression of F_1-score through the area under the F_1-score curve in lists ranked according to statistical significance (Figures S21, S22).

1.5. ROC and F_1-score analysis on SEQC data using normalization algorithms specific to each statistical algorithm

The overall performance of the individual tests and p-value combination methods in metaseqR when assessing them based on SEQC TaqMan data are not significantly altered when using each package specific normalization algorithm instead of EDASeq. The only exceptions are NOISeq whose performance drops dramatically and Fisher (Figure S11A, left panel). This is not the case for ERCC

spike-ins where there is a significant drop of performance for all algorithms and especially for NOISeq, which is now close to the ROC baseline (0.5) and Whitlock (Figure S11A, right panel). The same remarks apply when using adjusted p-values for DESeq, edgeR, limma voom and NBPSeq (Figures S24A, S24B).

2. Supplementary methods

2.1. The choice of weights for PANDORA

One of the main reasons for the development of PANDORA and metaseqR was to efficiently combine the advantages of several established statistical analysis algorithms. Hence, an intuitive approach to do so is to rank each algorithm according to its performance, either based on previous experience and/or related studies, or based on real-time simulations. In the latter case, virtual datasets can be constructed by drawing synthetic counts from a Negative Binomial distribution where the mean and dispersion parameters are estimated from the dataset under investigation. Consequently, the statistical analysis of the virtual datasets, where the differentially expressed genes and their expression levels are known beforehand, can be used to assess the performance of each algorithm with respect to a particular real dataset. A real-time ranking of the statistical tests is then possible and weights can be estimated and applied to the final statistical analysis of the original dataset. The performance of each statistical test can be assessed using established methods, for example ROC curves (sensitivity versus specificity) where the quantification of the area under the ROC curve represents a performance index, or FDCs, where the performance is measured by the number of false positive genes encountered in a list, ordered according to decreasing statistical significance. PANDORA uses the Area Under the False Discovery Curve to construct weights as follows: given an RNA-Seq dataset *D(n,c,m)*, where *n* is the number of genes, *c* the number of experimental conditions and *m* the total number of samples, we perform real-time *S* simulations, using *S* synthetic datasets constructed with a previously described simulator (4), the parameters of which are estimated from *D*. Subsequently, based on the *a priori* defined differentially expressed genes, we construct averaged FDCs for each applied statistical test and, using the AUFC under the first top *N=500* genes, we calculate convex weights with unit sum for each statistical test. Regarding the choice of *N*, it should not exceed the number of genes passing the statistical significance threshold (historically p-value or FDR less than 0.05), or encompass a number of genes containing false positives. Based on previous experience with the analysis of high-throughput genomics data, we set *N* to 500. The purpose of the unit sum, convex weighting scheme is that the resulting p-value and consequently the differentially expressed genes should be well within the boundaries set by the combination of the established statistical tests, thus taking into account their advantages without overestimating the number of differentially expressed genes and increasing the false positives. Figure S2 depicts such FDCs for six statistical tests supported by metaseqR. By analyzing the FDCs for the simulation set with three replicates and balanced differential expression (comprising a common experimental setting in real experiments), based on the five real datasets, we observe that the limma

voom algorithm performs better in terms of placing differentially expressed genes on the top of the lists, in accordance with previous studies (4-6), followed by baySeq and edgeR for all five organisms. NOISeq, NBPSeq and DESeq share the next three ranking positions in various orders among the five test cases. However, by observing also the FNCs (Figure S3), we note that the top performing algorithms present a higher number of false negatives as compared to the rest.

2.2. Quantitation of FDCs and FNCs

In order to calculate a quantitation value to characterize the performance of each method presented in the main text, we calculated the exact Area under the False Discovery (AUFC) or the False Negative (AUNC) Curves for the top $N=500$ or the bottom $M=2000$ ranked genes respectively, using arithmetic integration and functions provided in the R package `zoo`. Genes are ranked in the respective lists according to their statistical significance (from top to bottom for FDCs and from bottom to top for FNCs). Lower values for both AUFC and AUNC are indicative of best performing methods in terms of presenting a few false positives or a few false negatives, according to the respective ranking. The lowest possible value for the AUFC (or AUNC) depends on N (or M) and in our case is 499 (or 1999). Thus, in order to simplify the performance measure for each method, the AUFC (or AUNC) for each algorithm is divided by the respective lowest possible value. The result of this procedure is that values closer to 1 represent the best performing methods, both in terms of FDCs and FNCs. For example, an FDC performance value of 1 means that while traversing the top 500 statistically significant genes (ranked according to significance), no false positive was found. An FNC performance of 1 means that while traversing the bottom 2000 genes (ranked according to significance), no false negative was found. All calculations are averaged over 10 simulations.

2.3. Data input

The metaseqR package currently supports four methods of data input:

i) Aligned reads in SAM/BAM or BED format. In this case, the input files are passed to the metaseqR pipeline through a simply structured tab-delimited text file. This file can contain between three and five columns holding information regarding each sample. The first column must contain unique names that are used to identify each sample. The second column must contain the SAM/BAM/ BED file names (preferably with their full path) and the third column should provide biological conditions/groups for each sample/file. The fourth and fifth columns are optional and can contain information regarding whether the SAM/BAM files contain paired or single end reads and whether the library preparation protocol resulted in stranded reads or not. If both columns are provided, then information on paired end reads should be in the fourth

column and information regarding stranded reads should be in the fifth. The column for single or paired end read information should contain the work “single” for single end reads or the word “paired” for paired end reads. The column for stranded reads information should contain the word “yes” for stranded reads or the word “no” for unstranded reads. If the fourth and the fifth columns are not provided, single end unstranded reads are assumed. All this information is passed to the `summarizeOverlaps` function of the Bioconductor package GenomicRanges. The columns may or may not be named, as this information is not used by metaseqR. The above order (sample names, file names, biological condition, single or paired end reads, stranded reads) is used instead. This is the preferred method of data input, as in this way, the analysis is streamlined within R from beginning to end, ensuring data integrity (e.g. the compatibility of the genome annotations used, something that can occasionally be broken when using external read counting software). SAM/BAM files are imported through the Bioconductor packages Rsamtools and GenomicRanges and BED files are imported through the Bioconductor package rtracklayer. In the latter case, the fourth and fifth columns of the input text file are ignored. The aligned reads are converted to a read counts table through facilities provided in the Bioconductor package GenomicRanges. The Ensembl/UCSC/RefSeq gene or exon regions which are used to summarize the read alignments and create the final read counts table for each gene or exon can be obtained through downloading at the time of usage using the Bioconductor packages biomaRt and RMySQL or from a user-specified file satisfying some standards (the user should see examples in the package documentation).

ii) Summarized numbers of reads for each genomic feature (gene or exon) by providing a file containing the read counts table. This file should contain at least: a) a column with a unique identifier for each gene/exon (currently Ensembl, UCSC and RefSeq identifiers are supported unless the required annotation elements for each genomic region are embedded in the file), b) as many columns as the number of samples in the experiment. Each column with sample read counts should be named with a unique sample name. Optionally, the read counts file can contain annotation elements for each genomic feature (gene or exon) corresponding to the unique identifier column. In this case, the pipeline may be executed with the `annotation=”embedded”` argument, which is very useful when the user does not wish to use Ensembl/UCSC/RefSeq annotation or the organisms under investigation are not supported by metaseqR. If the user chooses to use embedded to the read counts file annotation, then, apart from its unique name, at least the following elements should be provided for each genomic region (in parenthesis next to each element, the required column name): chromosome where the genomic feature is located (chromosome), starting base pair in the chromosome (start), ending

base pair in the chromosome (end). For best performance (e.g. availability of all quality control and diagnostic plots), the following annotation elements should also be provided: GC content (gc_content) for genomic features of type “gene”, the gene model name (gene_name) for genomic features of type “exon”, the transcribing strand of each feature, denoted as “+” or “-“ (strand), HUGO (or other alias) gene name (gene_name) and each genomic features biotype/biological categorization, for example Ensembl’s categorizations like “protein_coding”, ”ncRNA”, “pseudogene”, etc. (biotype). If user needs are satisfied with one of metaseqR’s supporting annotations, it is better practice not to use embedded annotation in the counts file but download it.

iii) Like case (ii) but all the data mentioned in (ii) are stored in an R data frame object (for example, derived after a user-defined or otherwise customized preprocessing).

iv) A named list of the same length as the number of samples, where each member is named after the corresponding sample name. This list contains read counts for each exon of each gene and is the output of the `construct.gene.model` package function. It can be used only when `count.type=”exon”` and serves the purpose of not repeating the time-consuming step of exon assembly to gene models when an analysis has to be repeated with possibly different parameters.

In any of the above cases, some of the main input arguments to the pipeline can become mutually exclusive. For example, the user cannot supply both an input read counts table and a file with targets including sample filenames. Details on such issues can be found in the package documentation.

2.4. Data filtering

Two optional data filter types are implemented in the metaseqR package, operating at the exon (when exon counts are requested or provided to/from the pipeline) or the gene level (applied after summarizing exon counts to gene counts when exon counts are provided, or applied to gene counts if only a gene read counts table is provided). It should also be noted that, like the metaseqR pipeline, these filters are created for only the detection of differential expression at the gene level and not at the exon level or the detection of differential splicing.

2.4.1. Exon filters

Currently only one exon filter is implemented in metaseqR. This filter excludes genes that do not have enough reads presence in a fraction of their exons. This fraction should be moderate enough to avoid excluding genes that are possibly simply differentially spliced (although metaseqR is not intended to detect differential splicing), but stringent enough to exclude artifacts. This filter aims to exclude genes

containing “spikes” of read data in their UTR regions or a couple of their exons. These spikes are usually artifacts that can affect the subsequent differential expression analysis. The exon filter has three parameters: `exons.per.gene`, `min.exons` and `frac` and is applied as follows: if a gene has up to `exons.per.gene` exons, then read presence is required in at least `min.exons` of them, else read presence is required in a `frac` fraction of the total exons in the gene mode. With the default values, the filter instructs that if a gene has up to 5 exons, read presence is required in at least 2, else in at least 20% of its exons, in order to be included in further analysis. More filters will be implemented in future versions and users are encouraged to propose exon filter ideas.

After the determination of the genes that will be filtered from further processing (normalization and/or statistical analysis), a gene model expression value is constructed based on the sum of all exons of an annotated Ensembl/UCSC/RefSeq gene. It should be noted that while this particular way of summarizing a gene expression value is not recommended in applications where, for example, differential splicing, differential isoform expression or differential exon usage is studied, it is sufficient for most applications where only expression of a gene as a total is the goal of the study. Thus, it should be sufficient for a majority of related projects, where the researchers are interested in summarized gene expression values.

2.4.2. Gene filters

Gene filters are applied to gene expression as this is manifested through the read presence on each gene and can be applied before or after normalization. While for some categories this is not important (e.g. gene length filter), for others (e.g. expression filters), the application prior to or post normalization is important, as any expression filter must be applied to normalized data. In that case, metaseqR performs two rounds of normalization. The first round serves as a temporary normalization in order to get normalized expression values. The expression filters are then applied and genes not passing the filters are excluded from the second and final round of normalization. The gene filters can be applied both when the pipeline input consists of exon read counts and gene read counts. Such filters can for example be verbalized to "accept all genes above a certain count threshold" or "accept all genes with expression above the median of the normalized counts distribution" or "accept all genes with length above a certain threshold in kb" or "exclude the 'pseudogene' biotype from further analysis". Currently, there are four categories of gene filters. The first category is a qualitative filter, specifically a gene length filter where genes are accepted for further analysis if they are above a certain (the filter parameter) length. The second category consists of a combined qualitative/quantitative filter, where a gene is accepted for further analysis if it has more average reads per *x* bp than the quantile of the average normalized count distribution per *x* bp in the gene body. This filter `avg.reads` has two parameters: `average.per.bp`

expressing the number of base pairs for which reads are summarized and `quantile` for the quantile of the averaged normalized count distribution. The latter quantiles are calculated for each normalized sample and genes passing the filter should have an average read count larger than the maximum of the quantiles vector calculated above. The third category consists of a set of expression filters which can be applied together or only in subsets. The expression filters are the following:

i) A global median filter, where genes below the median of the global normalized count distribution in all samples are not accepted for further analysis (this filter has been used to distinguish between "expressed" and "not expressed" genes in several cases, e.g. (7)). The value of this filter is a boolean, `TRUE` for applying this filter and `FALSE` for not applying.
ii) A global mean filter, similar to the global median filter, but using the global mean.
iii) A global quantile filter, which is the same as the previous two, but using a specific quantile of the total counts distribution
iv) A filter based on the expression of genes known to be specifically expressed (e.g. not expressed) under the system under investigation. In this case, a set of known not-expressed genes is used to estimate an expression cutoff. This can be quite useful, as the genes are filtered based on a *true biological* cutoff instead of a statistical cutoff. The value of this filter is a character vector of HUGO gene symbols (which must be contained in the annotation, see previous section). Thus, if the user intends to use this filter, it is advisable to instruct metaseqR to download annotation on the fly or use the annotations embedded in the package. Then, these genes are used to build a "null" expression distribution. The 90th quantile of this distribution is then the expression cutoff. This filter can be combined with any other filter. The user should be careful with gene names, as they are case sensitive and must match exactly ("Pten" is different from "PTEN")

The fourth filter category is a qualitative filter based on the biological categorization of each gene (for example using Ensembl biotypes). In this case, genes with a certain biotype (which must be contained in the annotation) are excluded from the analysis.

2.5. Data normalization

The metaseqR package currently supports eight count data normalization algorithms from five different RNA-Seq analysis Bioconductor packages. Each package may provide additional options for normalization (e.g. more than one normalization algorithm present in a package, for example the NOISeq package), which can be controlled through normalization options (`norm.opts` parameter) passed to the pipeline call. The initial normalization parameters are the default parameters provided by the authors of each package. Specifically, metaseqR supports normalization with the EDASeq package

(8), which is the default option, with the edgeR package (9), with the NOISeq package (10) and the NBPSeq package (11). There is also an option to not normalize the data (not recommended). Popular normalization methods (e.g. RPKM normalization) are not directly supported, as they are coded within the current package and can be used by changing the normalization parameters passed to the pipeline. For example, RPKM normalization can be performed with the NOISeq package, although this is not currently recommended due to RPKM limitations discussed in several articles (e.g. (8)).

Data normalization can be performed before or after data filtering. The issue of applying normalization to the total dataset or to a filtered instance of the dataset is not sufficiently treated in the relative literature and there is not a definite answer. Our own experience suggests that normalization is smoother and the statistical analysis consequently more accurate, when normalization is applied after filtering. In the case of pre-normalization filtering, a first round of normalization applied as certain thresholds (e.g. gene expression thresholds) must be defined based on the global distributions of normalized data; otherwise, biases present in individual samples will cause confusion. For example, if the user filters data below a specific quantile of the normalized count distribution and that quantile is not determined based on normalized data, it will not be representative of the global data distribution but will produce a biased estimation dependent on the initial count distribution of the un-normalized samples. After the first normalization round, filters are applied and the filtered un-normalized data are normalized again. In the case of post-normalization filtering, data are first normalized and then filtered. In either case (pre- or post-normalization filtering), genes with zero counts across all samples are removed prior to normalization (as also suggested by most package authors).

2.6. Statistical testing

The metaseqR package currently supports nine statistical testing algorithms developed for RNA-Seq data. The algorithms supported are the testing procedures in the Bioconductor packages DESeq (12), edgeR (9), NOISeq (10), baySeq (13), limma voom (5) and NBPSeq (11). The arguments required for each statistical testing algorithm are passed by the metaseqR pipeline through the argument `stat.opts` (please refer to the package documentation for instructions on how to use the argument). The default options for each algorithm are the same as the corresponding default arguments used by the authors of each package. The default algorithm used by metaseqR is DESeq.

2.7. p-value combination

When analyzing data with metaseqR, the user may use more than one statistical testing method (any combination of the nine currently supported, implemented in six Bioconductor packages). In this case, metaseqR will combine the p-values that will be returned from each of the applied statistical tests and

will also report, apart from the p-value from each method, a combined p-value using one of the following methods:

i) The Simes p-value combination method (14). This method uses the minimum ordered p-value from all methods divided by the inverse order of the p-values. The ordering is performed across the number of statistics used. This is the default method.

ii) Fisher's p-value combination meta-analysis method (15), implemented in the R package MADAM.

iii) Same as (ii) but using permutations, as implemented in the R package MADAM. This option is quite computationally intensive and requires (much more) additional running time.

iv) Whitlock's p-value combination method (16). This method has the advantage of allowing weighting for each methodology. In the current version of metaseqR, the initial weights are equal for all statistical tests and can be changed by the user. For example, the user may estimate weights for his/her dataset using metaseqR facilities for this purpose or use one of the predefined weight sets for five organisms.

v) The maximum p-value returned by a set of statistical tests for the same gene. This is equivalent to the "intersection" of the results derived from each statistical test, returning genes which have been found as statistically significant by all the statistical tests applied. The maximum p-value ensures that the false positives are minimized at a (usually high) cost on the true positives (statistical power).

vi) The minimum p-value returned by a set of statistical tests for the same gene. This is equivalent to the "union" of the results derived from each statistical test, returning genes which have been found as statistically significant by at least one of the statistical tests applied. The minimum p-value ensures that the true positives are maximized at a (usually high) cost on the false positives (type I error).

vii) A weighted p-value where the weights can be either fixed (e.g. equal or set by the user according to performance evidence from the literature), or estimated using metaseqR's facilities for this purpose (simulation based on the user data and weighting according to performance measurement on simulated data). The weights must sum to 1. A set of pre-defined weights for each of metaseqR's supported organisms can be used or the weights can be estimated based on the user's data, provided sufficiency of samples (preferably more than 6 samples across no more than two conditions). The function `estimate.aufc.weights` can be used to this end. This method corresponds to the PANDORA method presented in the main article.

viii) A method based on permutations. This method has three variants:

a. In the first variant (`dperm.min`), an initial p-value vector is constructed for each gene, containing the minimum p-value resulting from the applied statistical tests (looser, see above).

b. In the second variant (`dperm.max`), an initial p-value vector is constructed for each gene, containing the maximum p-value resulting from the applied statistical tests (stricter, see above).

c. In the third variant (`dperm.weight`), an initial p-vale vector is constructed for each gene, containing the convex linear combination of the p-values resulting from all the statistical tests applied for each gene. To construct the convex linear combination, a vector of weights is used, one for each statistical test, and the sum of all weights must be 1.

After the construction of the original combined p-value with one of the aforementioned variants, a permutation procedure is initiated, where `nperm` permutations are performed across the samples of the normalized counts matrix, producing `nperm` permuted instances of the initial dataset. Then, all the chosen statistical tests are re-executed for each permutation. The final p-value is the number of times that the p-value of the permuted datasets is smaller than the original dataset. The p-value of the original dataset is created based on the choice of one. Generally, the permutation procedure usually requires a lot of time in order to yield accurate results (at least 10000 iterations especially in smaller datasets). Additionally, this method will NOT work when there are no replicated samples across biological conditions. In that case, the Simes method or one of the other methods should be used.

It should be noted that in the case of NOISeq, the significance value returned is not similar to the "classic" t-test like statistics, thus its inclusion in a meta-analysis should be handled and interpreted with caution. Furthermore, the meta-analysis feature provided by metaseqR is currently experimental and does not satisfy the strict definition of "meta-analysis", which is the combination of multiple similar datasets under the same statistical methodology. Instead it is the use of multiple statistical tests applied to the same data, so the results at this point are not guaranteed and should be interpreted appropriately. We are working on a more solid methodology for combining multiple statistical tests based on multiple testing correction and Monte Carlo methods.

2.8. Diagnostic plots

metaseqR supports a variety of diagnostic plots, some of which are adopted from the analysis packages supporting metaseqR, while others are metaseqR specific. A list of possible diagnostic plots follow, coupled with a short explanation. The short explanations provided here are also provided as interpretation hints inside each metaseqR report.

- *Multidimensional scaling plots.* The Multi-Dimensional Scaling (MDS) plots comprise a means of visualizing the level of similarity of individual cases of a dataset. It is similar to Principal Component Analysis (PCA), but instead of using the covariance matrix to find similarities among cases, MDS uses absolute distance metrics such as the classical Euclidean distance. Because of the relative linear relations among sequencing samples, it provides a more realistic clustering among samples. MDS serves quality control and can be interpreted as follows: when the distance among samples of the same biological condition in the MDS space is small, this is an indication of high correlation and reproducibility among them. When this distance is larger or heterogeneous (e.g. the 3rd sample of a triplicate set is further from the other 2), this constitutes an indication of low correlation and reproducibility among samples. It can help exclude poor samples from further analysis.

- *Biotype detection plots.* The biotype detection bar diagrams are a set of quality control charts that show the percentage of each biotype in the genome (i.e. in the whole set of features provided, for example, protein coding genes, non-coding RNAs or pseudogenes) in grey bars, which proportion has been detected in a sample before normalization and after a basic filtering by removing features with zero counts in red lined bars, and the percentage of each biotype within the sample in solid red bars. The difference between grey bars and solid red bars is that the grey bars show the percentage of a feature in the genome while the solid red bars show the percentage in the sample. Thus, the solid red bars may be sometimes higher than the grey bars because certain features (e.g. protein coding genes) may be detected within a sample with a higher proportion relative to their presence in the genome, as compared with other features. For example, while the percentage of protein coding genes in the whole genome is already higher than other biotypes, this percentage is expected to be even higher in an RNA-Seq experiment where one expects protein-coding genes to exhibit greater abundance. The vertical green line separates the most abundant biotypes (on the left-hand side, corresponding to the left axis scale) from the rest (on the right-hand side, corresponding to the right axis scale). Otherwise, the lower abundance biotypes would be indistinguishable. Unexpected outcomes in this quality

control chart (e.g. very low detection of protein coding genes) would signify possible low quality of a sample.

- *Biotype detection count plots.* The biotype detection counts boxplots are a set of quality control charts that depict both the biological classification for the detected features and the actual distribution of the read counts for each biological type. The boxplot comprises a means of summarizing the read counts distribution of a sample in the form of a bar with extending lines, as a commonly used way of graphically presenting groups of numerical data. A boxplot also indicates which observations, if any, might be considered outliers and is able to visually show different types of populations, without making any assumptions of the underlying statistical distribution. The spacing between the different parts of the box help indicate variance, skewedness and identify outliers. The thick bar inside the colored box is the median of the observations while the box extends over the Interquartile Range of the observations. The whiskers extend up (down) to +/-1.5xIQR. Unexpected outcomes (e.g. protein coding read count distribution similar to pseudogene read count distribution) indicate poor sample quality.
- *Read and biotype saturation plots.* The read and biotype saturation plots are a set of quality control charts that depict the read count saturation levels at several sequencing depths. Thus, they comprise a means of assessing whether the sequencing depth of an RNA-Seq experiment is sufficient in order to detect the biological features under investigation. These quality control charts are separated in two subgroups: the first subgroup (read saturation per biotype for all samples) is a set of plots, one for each biological feature (e.g. protein coding, pseudogene, lincRNA, etc.), that depict the number of detected features in different sequencing depths and for all samples in the same plot. The second subgroup (read saturation per sample for all biotypes) is a set of plots similar to the above, but with one pair of plots with two panels for each sample, presenting all biological features. The left panel depicts the saturation levels for the less abundant features, while the right panel, the saturation for the more abundant features, as placing them all together would make the less abundant features indistinguishable. All the saturation plots should be interpreted as follows: if the read counts for a biotype tend to be saturated, the respective curve should tend to reach a plateau at higher depths. Otherwise, more sequencing is needed for the specific biotype.
- *RNA-Seq reads noise plots.* The read noise plots depict the percentage of biological features detected when subsampling the total number of reads. Very steep curves in read noise plots indicate that although the sequencing depth reaches its maximum, a relatively small percentage of total features is detected, indicating that the level of background noise is relatively high. Less

steep RNA composition curves indicate less noise. When a sample curve deviates from the rest, it could indicate lower or higher quality, depending on the curves of the rest of the samples.

- *Correlation plots.* The sample correlation plots depict the accordance among the RNA-Seq samples, as this is manifested through the read counts table used with the metaseqr pipeline, with two representations that both use the correlation matrix (a matrix which depicts all the pairwise correlations between each pair of samples) of the read counts matrix. The first is a correlation clustered heatmap which depicts the correlations among samples as color-scaled image and the hierarchical clustering tree depicts the grouping of the samples according to their correlation. Samples from the same group that are not clustered together provides an indication that there might be a quality problem with the dataset. The second is a 'correlogram' plot, where again the samples are hierarchically clustered and grouped, but this time correlations are presented as ellipses inside each cell. Each cell represents a pairwise comparison and each correlation coefficient is represented by an ellipse whose 'diameter', direction and color depict the accordance for that pair of samples. Highly correlated samples are depicted as ellipses with narrow diameter, while poorly correlated samples are depicted as ellipses with wide diameters. Also, highly correlated samples are depicted as ellipses with a left-to-right upwards direction, while poorly correlated samples are depicted as ellipses with a right-to-left upwards direction.
- *Pairwise scatterplots.* The pairwise comparison plots are split in three parts: the upper diagonal consists of simple scatterplots for all pairwise sample comparisons, together with their Pearson correlation coefficient. It is a simple measure of between sample correlations using all the available data points instead of only the correlation matrix. The lower diagonal consists of mean-difference plots for all pairwise sample comparisons. A mean-difference plot (or a Bland-Altman plot) is a method of data plotting used in analyzing the agreement between two different assays/variables. In this graphical method the differences (or alternatively the ratios) between the two variables are plotted against the averages of the two. Such a plot is useful, for example, for analyzing data with strong correlation between x and y axes, when the (x,y) dots on the plot are close to the diagonal x=y. In this case, the value of the transformed variable X is about the same as x and y and the variable Y shows the difference between x and y. In both representations, irregular shapes of the red smoother lines are an indication of poor correlation between samples or of other systematic bias sources, which is usually corrected through data normalization.
- *Boxplots.* The boxplot comprises a means of summarizing the read counts distribution of a sample in the form of a bar with extending lines, as a commonly used way of graphically

presenting groups of numerical data. A boxplot also indicates which observations, if any, might be considered outliers and is able to visually show different types of populations, without making any assumptions about the underlying statistical distribution. The spacings between the different parts of the box help indicate variance, skewedness and identify outliers. The thick bar inside the colored box is the median of the observations while the box extends over the Interquartile Range of the observations. The whiskers extend up (down) to +/-1.5xIQR. Boxplots at similar levels indicate good quality of the normalization. If boxplots remain at different levels after normalization, another normalization algorithm may have to be examined. The un-normalized boxplots show the need for data normalization in order for the data from different samples to follow the same underlying distribution and statistical testing becoming possible.

- *RNA composition plots.* The RNA composition plots depict the differences in the distributions of reads in the same biological features across samples. The following is taken from the NOISeq vignette: '*...when two samples have different RNA composition, the distribution of sequencing reads across the features is different in such a way that although a feature had the same number of read counts in both samples, it would not mean that it was equally expressed in both... To check if this bias is present in the data, the RNA composition plot and the corresponding diagnostic test can be used. In this case, each sample s is compared to the reference sample r (which can be arbitrarily chosen). To do that, M values are computed as log2(counts_sample = counts_reference). If no bias is present, it should be expected that the median of M values for each comparison is 0. Otherwise, it would be indicating that expression levels in one of the samples tend to be higher than in the other, and this could lead to false discoveries when computing differential expression. Confidence intervals for the M median are also computed by bootstrapping. If value 0 does not fall inside the interval, it means that the deviation of the sample with regard to the reference sample is statistically significant. Therefore, a normalization procedure is required.*'
- *GC-content bias plots.* The GC-content bias plot is a quality control chart that shows the possible dependence of the read counts (in log2 scale) under a gene to the GC content percentage of that gene. In order for the statistical tests to be able to detect statistical significance which occurs due to real biological effects and not by other systematic biases present in the data (e.g. a possible GC-content bias), the latter should be accounted for by the applied normalization algorithm. Although the tests are performed for each gene across biological conditions, one could assume that the GC content does not represent a bias, as it is

the same for the tested gene across samples and conditions. However, Risso et al. (2011) showed that the GC-content could have an impact in the statistical testing procedure. The GC-content bias plot depicts the dependence of the read counts on the GC content before and after normalization. The smoothing lines for each sample should be as 'straight' as possible after normalization. In addition, if the smoothing lines differ significantly between biological conditions, this would constitute a possible quality warning.

- *Gene/transcript length bias plots.* The gene/transcript length bias plot is a quality control chart that shows the possible dependence of the read counts (in log2 scale) under a gene to the length that gene (whole gene or sum of exons depending on the analysis). In order for the statistical tests to be able to detect statistical significance which occurs due to real biological effects and not by other systematic biases present in the data (e.g. a possible length bias), the latter should be accounted for by the applied normalization algorithm. Although the tests are performed for each gene across biological conditions, one could assume that the gene length does not represent a bias as it is the same for the tested gene across samples and conditions. However, it has been shown in several studies that the gene length could have an impact on the statistical testing procedure. The length bias plot depicts the dependence of the read counts to the gene/transcript length before and after normalization. The smoothing lines for each sample should be as 'straight' as possible after normalization. In addition, if the smoothing lines differ significantly among biological conditions, this would constitute a possible quality warning.
- *Mean-difference plots.* A mean-difference plot (or a Bland-Altman plot) is a method of data plotting used in analyzing the agreement between two different assays/variables. In this graphical method the differences (or alternatively the ratios) between the two variables are plotted against the averages of the two. Such a plot is useful, for example, for analyzing data with strong correlation between x and y axes, when the (x,y) dots on the plot are close to the diagonal x=y. In this case, the value of the transformed variable X is about the same as x and y and the variable Y shows the difference between x and y. When the data cloud in a mean difference plot is centered around the horizontal zero line, this is an indication of good data quality and good normalization results. On the other hand, when the data cloud deviates from the center line or has a 'banana' shape, this constitutes an indication of systematic biases present in the data and that either the chosen normalization algorithm has not worked well, or that data are not normalized. The smoothing curve that traverses the data summarizes the above trends.
- *Mean-variance plots.* The mean-variance plot comprises a graphical means of displaying a possible relationship between the means of gene expression (counts) values and their variances

across replicates of the same biological condition. Thus data can be inspected for possible overdispersion (greater variability in a dataset than would be expected based on a given simple statistical model). In such plots for RNA-Seq data, overdispersion is usually manifested as increasing variance with increasing gene expression (counts) and it is summarized through a smoothing curve (red curve). The following is taken from the EDASeq package vignette: '*...although the Poisson distribution is a natural and simple way to model count data, it has the limitation of assuming equality of the mean and variance. For this reason, the negative binomial distribution has been proposed as an alternative when the data show over-dispersion...*' If overdispersion is not present, the data cloud is expected to be evenly scattered around the smoothing curve.

- *Chromosome and biotype distribution of filtered genes.* The chromosome and biotype distribution of filtered genes is a quality control chart with two rows and four panels: on the left panel of the first row, the bar chart depicts the numbers of filtered genes per chromosome (actual numbers shown above the bars). On the right panel of the first row, the bar chart depicts the numbers of filtered genes per biotype (actual numbers shown above the bars). On the left panel of the second row, the bar chart depicts the fraction of the filtered genes to the total genes per chromosome (actual percentages shown above the bars). On the right panel of the second row, the bar chart depicts the fraction of the filtered genes to the total genes per biotype (actual percentages shown above the bars). This plot should indicate possible quality problems when, for example, the filtered genes for a specific chromosome (or the fraction) is extremely higher than the rest. Generally, the fractions per chromosome should be uniform and the fractions per biotype should be proportional to the biotype fraction relative to the genome.
- *Volcano plots.* A volcano plot is a scatterplot that is often used when analyzing high-throughput -omics data (e.g. microarray data, RNA-Seq data) to give an overview of interesting genes. The log2 fold change is plotted on the x-axis and the negative log10 p-value is plotted on the y-axis. A volcano plot combines the results of a statistical test (aka, p-values) with the magnitude of the change enabling quick visual identification of those genes that display large-magnitude changes that are also statistically significant. The horizontal dashed line sets the threshold for statistical significance, while the vertical dashed lines set the thresholds for biological significance. It should be noted that the volcano plots become harder to interpret when using more than one statistical algorithm and performing meta-analysis. This happens because the genes that have stronger evidence of being differentially expressed obtain lower p-values, while the rest either remain at similar levels or obtain higher p-values. The result is a 'warped' volcano

plot, with two main data clouds: one in the upper part of the plot, and one in the lower part of the plot. You can always zoom in when using interacting mode (the default).

- *DEG heatmaps.* The Differentially Expressed Genes (DEGs) heatmaps depict how well samples from different conditions cluster together according to their expression values after normalization and statistical testing, for each requested statistical contrast. If samples from the same biological condition do not cluster together, this would constitute a warning sign regarding the quality of the samples. In addition, DEG heatmaps provide an initial view of possible clusters of co-expressed genes.
- *Chromosome and biotype distributions of DEGs.* The chromosome and biotype distributions bar diagram for Differentially Expressed Genes (DEGs) is split in two panels: i) on the left panel DEGs are distributed per chromosome and the percentage of each chromosome in the genome is presented in grey bars, the percentage of DEGs in each chromosome is presented in red lined bars and the percentage of certain chromosomes in the distribution of DEGs is presented in solid red bars. ii) on the right panel, DEGs are distributed per biotype and the percentage of each biotype in the genome (i.e. in the whole set of features provided, for example, protein coding genes, non-coding RNAs or pseudogenes) is presented in grey bars, the percentage of DEGs in each biotype is presented in blue lined bars and the percentage of each biotype in DEGs is presented in solid blue lines. The vertical green line separates the most abundant biotypes (on the left-hand side, corresponding to the left axis scale), from the rest (on the right-hand side, corresponding to the right axis scale). Otherwise, the lower abundance biotypes would be indistinguishable.
- *Venn diagrams.* The Venn diagrams are an intuitive way of presenting overlaps between lists, based on the overlap of basic geometrical shapes. The numbers of overlapping genes per statistical algorithm are shown in the different areas of the Venn diagrams, one for each contrast.

2.9. Data export and reporting

The final product of metaseqR is an extended HTML report, including an auto-generated summary that can be used e.g. in the "Methods" section of a scientific article, all the analysis parameters in an eye-friendly format, several diagnostic plots and the final gene lists. The latter are exported in text tab delimited format and as such can be easily imported either to spreadsheet software or manipulated in further analyses. The output gene lists are of two kinds (the lists of differentially expressed genes for each comparison defined by the user and the total list of genes in the organism, including the filtered

ones) and they can be retrieved through the report or from the resulting directory structure that metaseqR creates. The report lives in the folder specified by the `export.where` parameter. The report is accessed through the file `index.html`. Although the output gene lists and figures can be accessed through the report, they can be retrieved by the respective directory structure created by the pipeline:

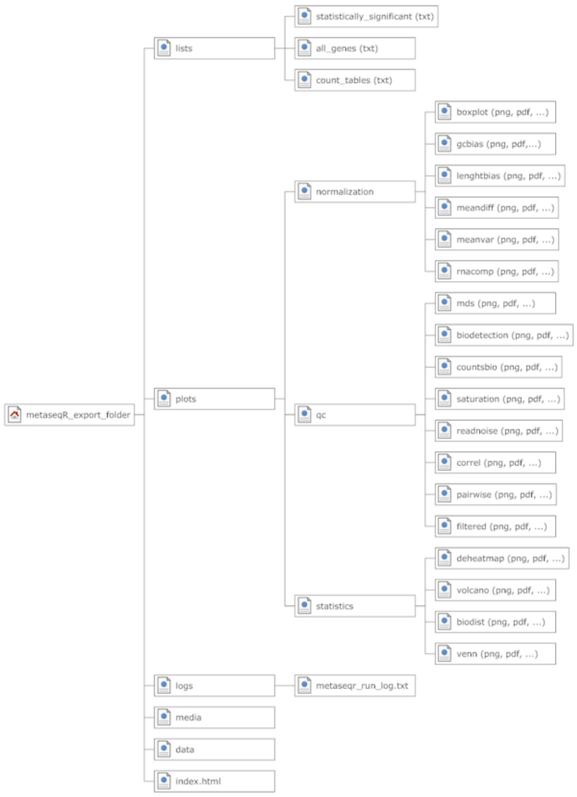

Additional information regarding export options can be found in the package manual and help pages.

3. Supplementary figures

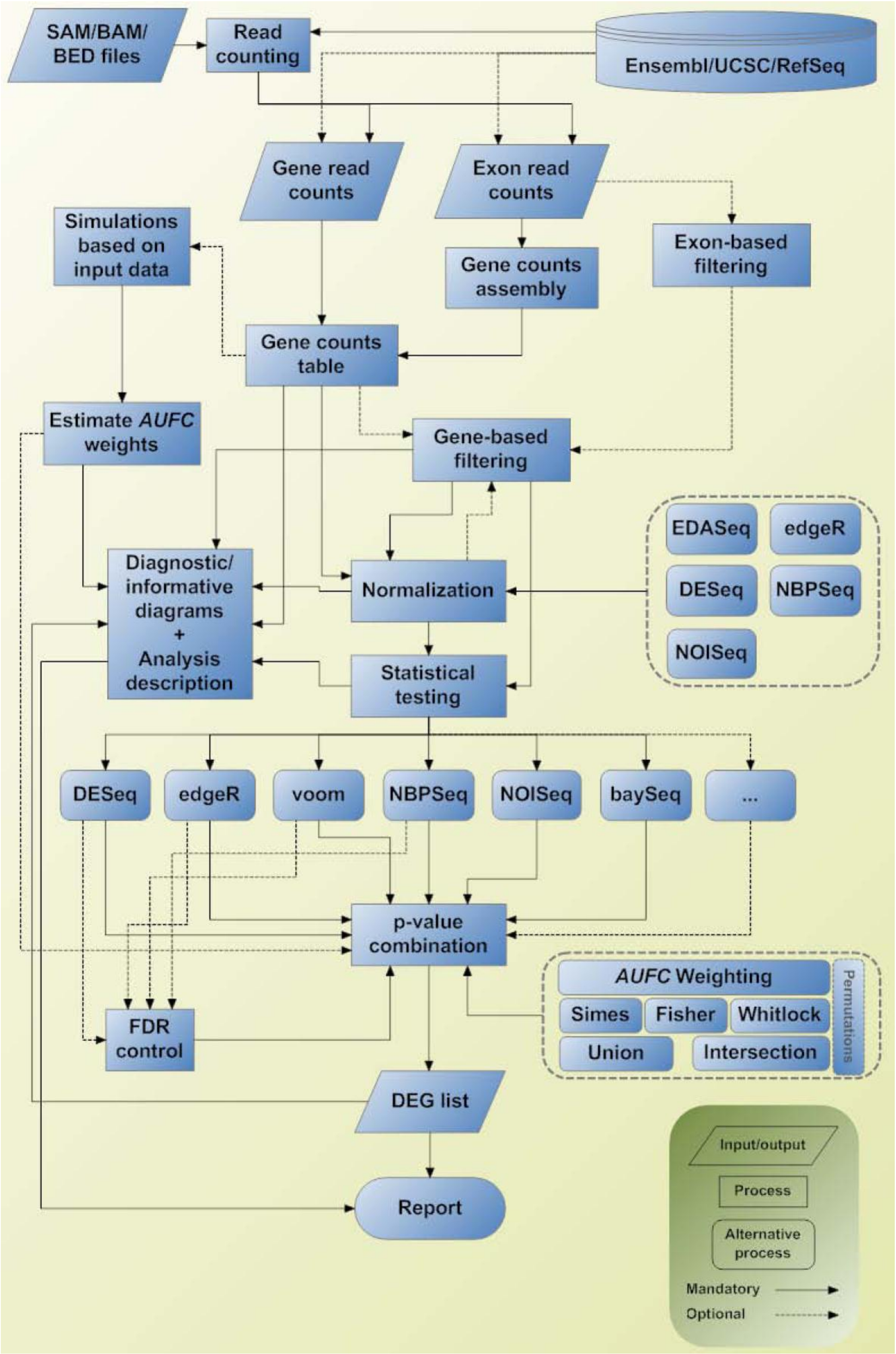

Figure S1: The analysis workflow of metaseqR

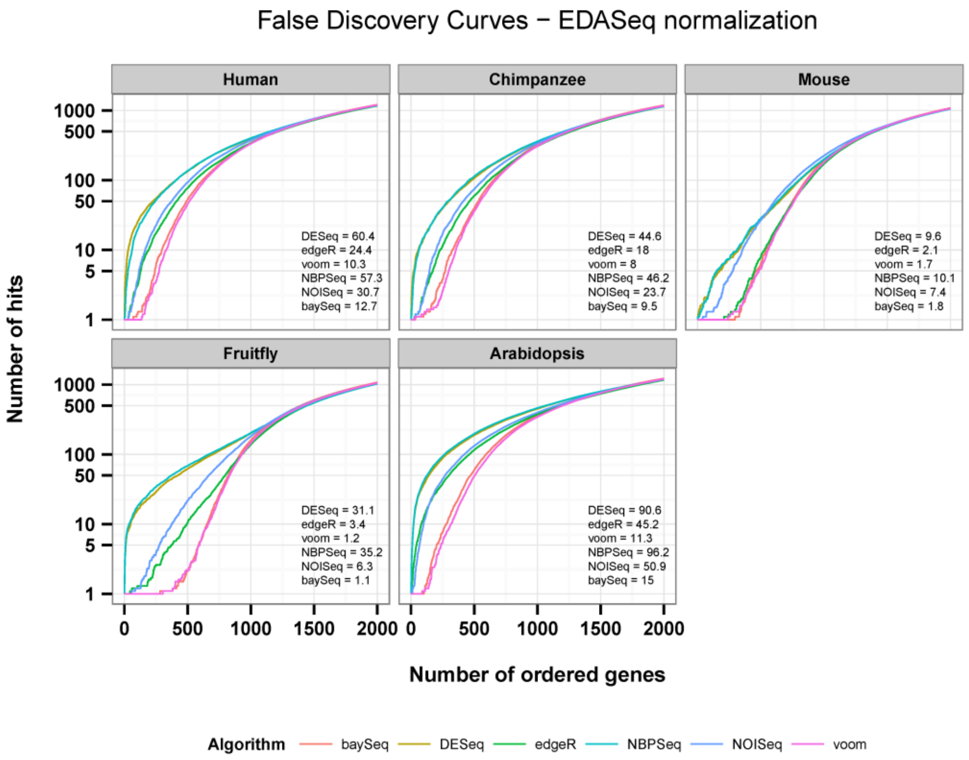

Figure S2: False Discovery Curves for six statistical tests using EDASeq normalization

FDCs for six RNA-Seq data statistical tests applied to simulated data from five organisms using EDASeq as the common normalization algorithm in a simulation with two hypothetical biological conditions, each with three replicates and balanced differential expression between the two. The title of each panel corresponds to a model organism. Their performance measurements according to their AUFC (Supplementary methods) are displayed next to the curves. limma voom shows the smallest value in all cases, thus its AUFC weight is larger for all organisms.

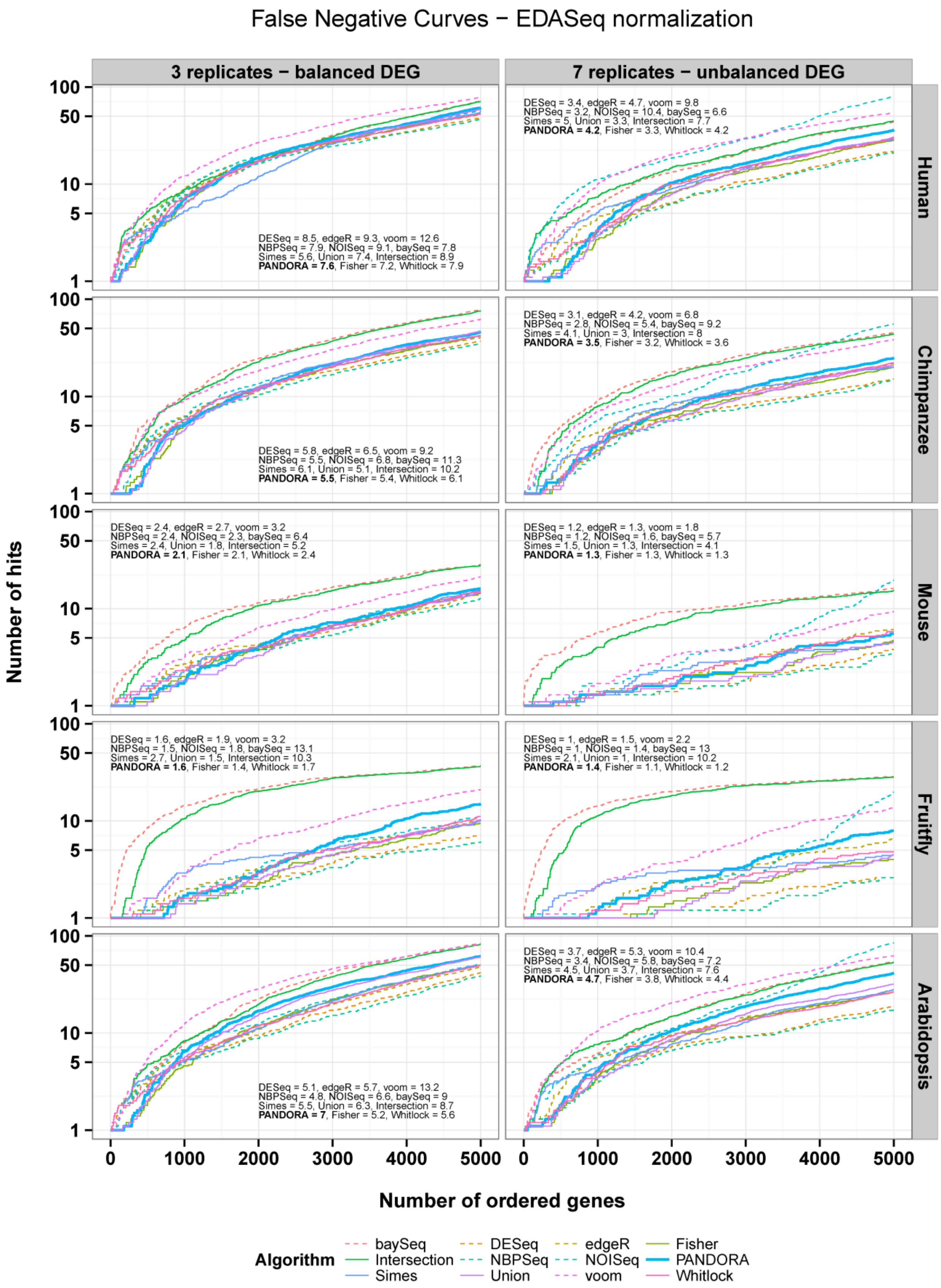
False Negative Curves − EDASeq normalization
3 replicates − balanced DEG
7 replicates − unbalanced DEG
Human
Chimpanzee
Mouse
Fruitfly
Arabidopsis
Number of hits
Number of ordered genes
DESeq = 8.5, edgeR = 9.3, voom = 12.6
NBPSeq = 7.9, NOISeq = 9.1, baySeq = 7.8
Simes = 5.6, Union = 7.4, Intersection = 8.9
PANDORA = 7.6, Fisher = 7.2, Whitlock = 7.9
DESeq = 3.4, edgeR = 4.7, voom = 9.8
NBPSeq = 3.2, NOISeq = 10.4, baySeq = 6.6
Simes = 5, Union = 3.3, Intersection = 7.7
PANDORA = 4.2, Fisher = 3.3, Whitlock = 4.2
DESeq = 5.8, edgeR = 6.5, voom = 9.2
NBPSeq = 5.5, NOISeq = 6.8, baySeq = 11.3
Simes = 6.1, Union = 5.1, Intersection = 10.2
PANDORA = 5.5, Fisher = 5.4, Whitlock = 6.1
DESeq = 3.1, edgeR = 4.2, voom = 6.8
NBPSeq = 2.8, NOISeq = 5.4, baySeq = 9.2
Simes = 4.1, Union = 3, Intersection = 8
PANDORA = 3.5, Fisher = 3.2, Whitlock = 3.6
DESeq = 2.4, edgeR = 2.7, voom = 3.2
NBPSeq = 2.4, NOISeq = 2.3, baySeq = 6.4
Simes = 2.4, Union = 1.8, Intersection = 5.2
PANDORA = 2.1, Fisher = 2.1, Whitlock = 2.4
DESeq = 1.2, edgeR = 1.3, voom = 1.8
NBPSeq = 1.2, NOISeq = 1.6, baySeq = 5.7
Simes = 1.5, Union = 1.3, Intersection = 4.1
PANDORA = 1.3, Fisher = 1.3, Whitlock = 1.3
DESeq = 1.6, edgeR = 1.9, voom = 3.2
NBPSeq = 1.5, NOISeq = 1.8, baySeq = 13.1
Simes = 2.7, Union = 1.5, Intersection = 10.3
PANDORA = 1.6, Fisher = 1.4, Whitlock = 1.7
DESeq = 1, edgeR = 1.5, voom = 2.2
NBPSeq = 1, NOISeq = 1.4, baySeq = 13
Simes = 2.1, Union = 1, Intersection = 10.2
PANDORA = 1.4, Fisher = 1.1, Whitlock = 1.2
DESeq = 5.1, edgeR = 5.7, voom = 13.2
NBPSeq = 4.8, NOISeq = 6.6, baySeq = 9
Simes = 5.5, Union = 6.3, Intersection = 8.7
PANDORA = 7, Fisher = 5.2, Whitlock = 5.6
DESeq = 3.7, edgeR = 5.3, voom = 10.4
NBPSeq = 3.4, NOISeq = 5.8, baySeq = 7.2
Simes = 4.5, Union = 3.7, Intersection = 7.6
PANDORA = 4.7, Fisher = 3.8, Whitlock = 4.4
Algorithm
baySeq
Intersection
Simes
DESeq
NBPSeq
Union
edgeR
NOISeq
voom
Fisher
PANDORA
Whitlock

Figure S3: False Negative Curves using EDASeq normalization and unadjusted p-values

FNCs generated with simulated data for each statistical test and each p-value combination method supported by metaseqR for five organisms and two simulation configurations. The results for each organism and simulation configuration can be distinguished by the right and top side titles of each panel, referring to the organism from which simulation parameters are estimated and the simulation configuration, respectively. The performance value according to the AUFC is displayed in each panel next to the curves (Supplementary methods). The lowest possible value is 1, indicating no false negative hits among the first 2000 ranked genes according to increasing statistical significance (from higher to lower p-values). Dashed lines represent individual tests whereas solid lines represent p-value combinations and the thicker solid line highlights the FNC produced by PANDORA. limma voom and baySeq are constantly among the bottom performing algorithms in terms of missing true positive hits. PANDORA is quite stable, changing slightly performance positions according to simulation configuration and organism. As expected, higher numbers of replicates increases the performance of all algorithms in terms of losing true positives (right panels). The curves as well as their performance values are constructed and calculated respectively across ten simulations for each organism and simulation configuration.

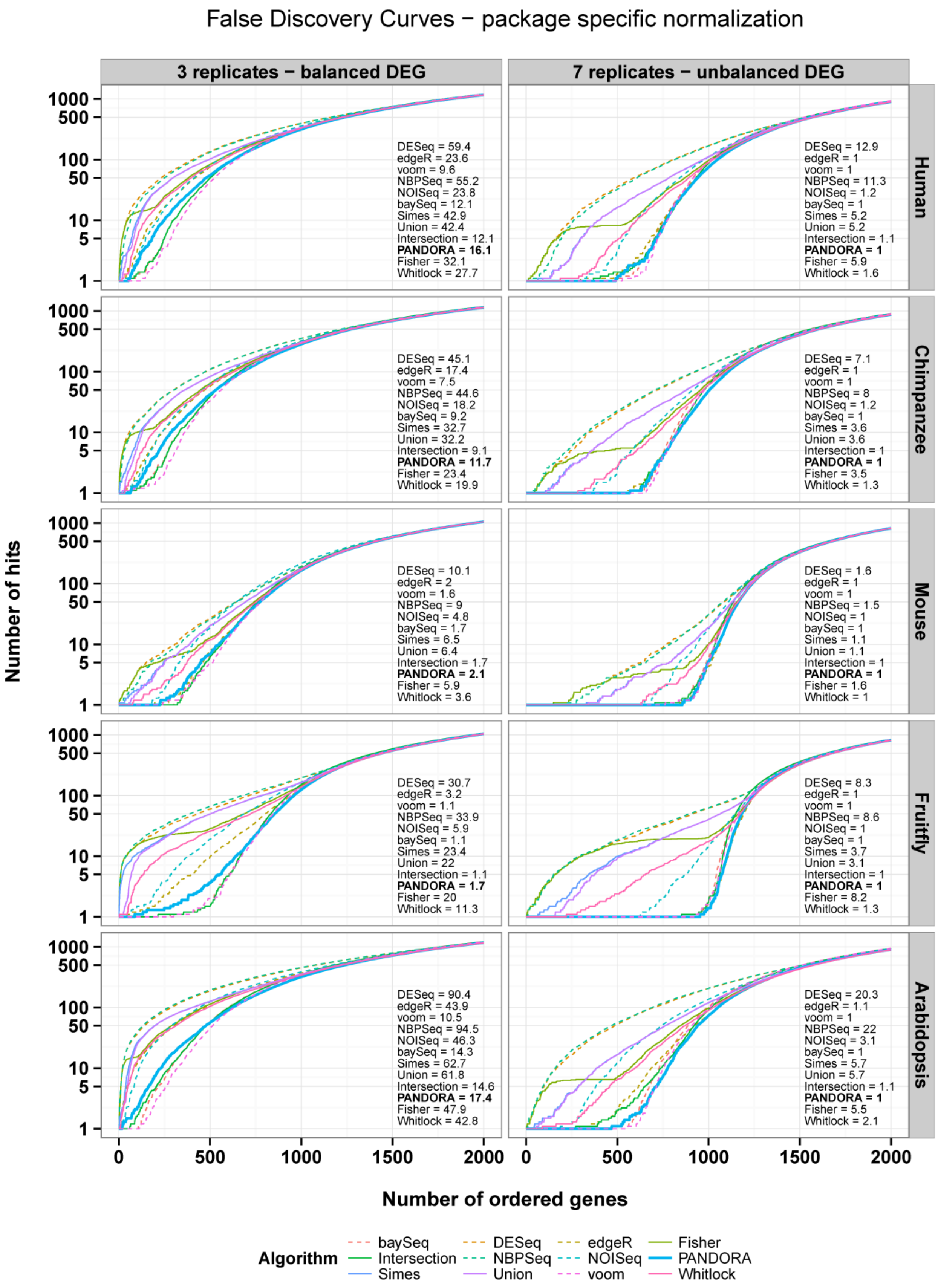
False Discovery Curves − package specific normalization
3 replicates − balanced DEG
7 replicates − unbalanced DEG
Human
Chimpanzee
Mouse
Fruitfly
Arabidopsis
Number of hits
Number of ordered genes
1000
500
100
50
10
5
1
0
500
1000
1500
2000
DESeq = 59.4
edgeR = 23.6
voom = 9.6
NBPSeq = 55.2
NOISeq = 23.8
baySeq = 12.1
Simes = 42.9
Union = 42.4
Intersection = 12.1
PANDORA = 16.1
Fisher = 32.1
Whitlock = 27.7
DESeq = 12.9
edgeR = 1
voom = 1
NBPSeq = 11.3
NOISeq = 1.2
baySeq = 1
Simes = 5.2
Union = 5.2
Intersection = 1.1
PANDORA = 1
Fisher = 5.9
Whitlock = 1.6
DESeq = 45.1
edgeR = 17.4
voom = 7.5
NBPSeq = 44.6
NOISeq = 18.2
baySeq = 9.2
Simes = 32.7
Union = 32.2
Intersection = 9.1
PANDORA = 11.7
Fisher = 23.4
Whitlock = 19.9
DESeq = 7.1
edgeR = 1
voom = 1
NBPSeq = 8
NOISeq = 1.2
baySeq = 1
Simes = 3.6
Union = 3.6
Intersection = 1
PANDORA = 1
Fisher = 3.5
Whitlock = 1.3
DESeq = 10.1
edgeR = 2
voom = 1.6
NBPSeq = 9
NOISeq = 4.8
baySeq = 1.7
Simes = 6.5
Union = 6.4
Intersection = 1.7
PANDORA = 2.1
Fisher = 5.9
Whitlock = 3.6
DESeq = 1.6
edgeR = 1
voom = 1
NBPSeq = 1.5
NOISeq = 1
baySeq = 1
Simes = 1.1
Union = 1.1
Intersection = 1
PANDORA = 1
Fisher = 1.6
Whitlock = 1
DESeq = 30.7
edgeR = 3.2
voom = 1.1
NBPSeq = 33.9
NOISeq = 5.9
baySeq = 1.1
Simes = 23.4
Union = 22
Intersection = 1.1
PANDORA = 1.7
Fisher = 20
Whitlock = 11.3
DESeq = 8.3
edgeR = 1
voom = 1
NBPSeq = 8.6
NOISeq = 1
baySeq = 1
Simes = 3.7
Union = 3.1
Intersection = 1
PANDORA = 1
Fisher = 8.2
Whitlock = 1.3
DESeq = 90.4
edgeR = 43.9
voom = 10.5
NBPSeq = 94.5
NOISeq = 46.3
baySeq = 14.3
Simes = 62.7
Union = 61.8
Intersection = 14.6
PANDORA = 17.4
Fisher = 47.9
Whitlock = 42.8
DESeq = 20.3
edgeR = 1.1
voom = 1
NBPSeq = 22
NOISeq = 3.1
baySeq = 1
Simes = 5.7
Union = 5.7
Intersection = 1.1
PANDORA = 1
Fisher = 5.5
Whitlock = 2.1
Algorithm
baySeq
Intersection
Simes
DESeq
NBPSeq
Union
edgeR
NOISeq
voom
Fisher
PANDORA
Whitlock

Figure S4: False Discovery Curves using each package's specific normalization and unadjusted p-values

FDCs generated with simulated data for each statistical test and each p-value combination method supported by metaseqR for five organisms and two simulation configurations, using the normalization method suggested by each individual package instead of EDASeq. The results for each organism and simulation configuration can be distinguished by the right and top side titles of each panel, referring to the organism from which simulation parameters are estimated and the simulation configuration, respectively. The performance value according to the AUFC is displayed in each panel next to the curves (Supplementary methods). The lowest possible value is 1, indicating no false discoveries among the first 500 top ranked genes according to statistical significance. Dashed lines represent individual tests whereas solid lines represent p-value combinations and the thicker solid line highlights the FDC produced by PANDORA. limma voom is constantly among the best performing algorithms and PANDORA among the top methods in general. As expected, higher numbers of replicates increases the accuracy and performance of all algorithms (right panels). The curves as well as the performance values are constructed and calculated across ten simulations for each organism and simulation configuration, respectively.

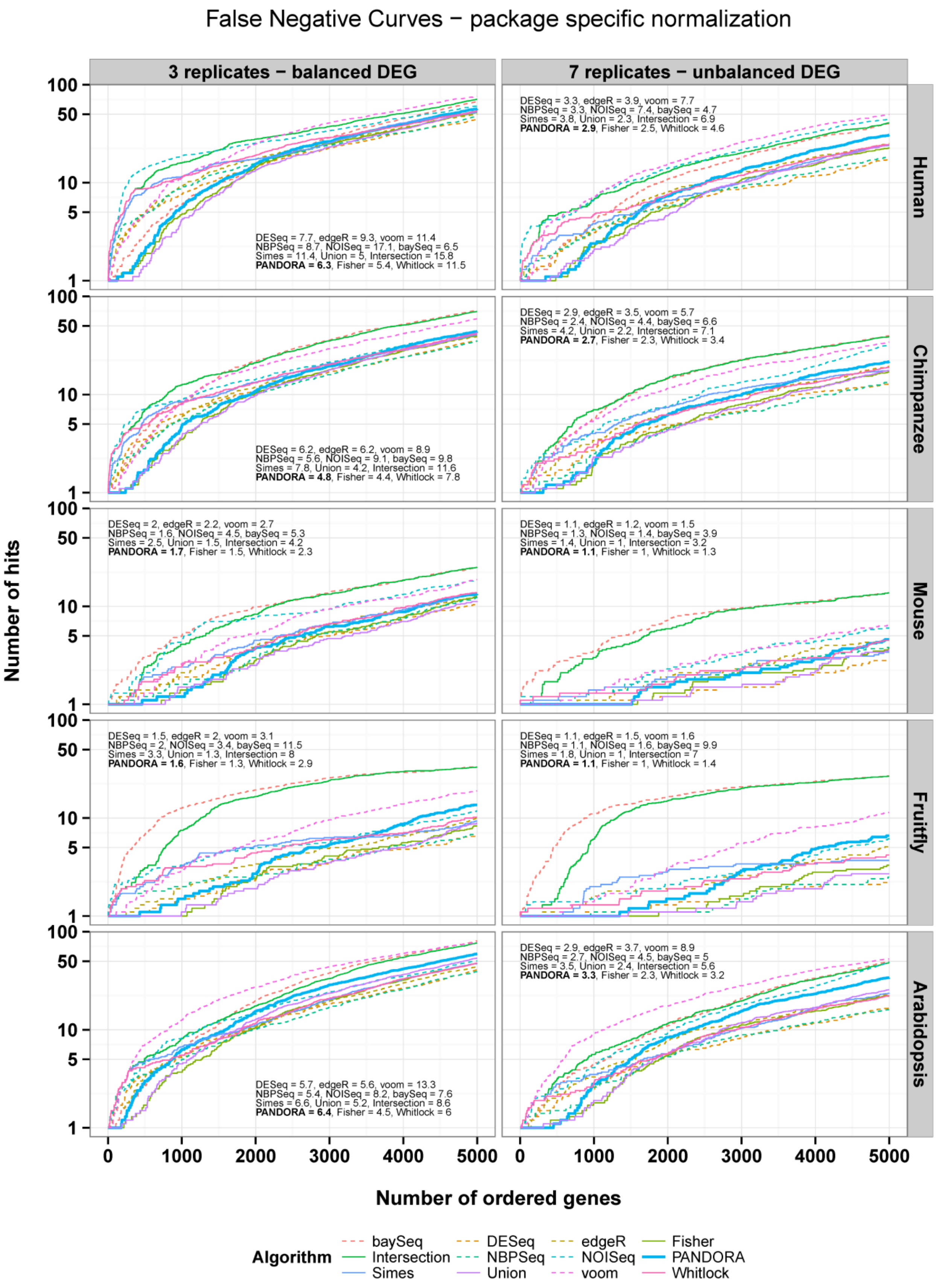

False Negative Curves − package specific normalization
3 replicates − balanced DEG
7 replicates − unbalanced DEG
Human
Chimpanzee
Mouse
Fruitfly
Arabidopsis
Number of hits
Number of ordered genes
DESeq = 7.7, edgeR = 9.3, voom = 11.4
NBPSeq = 8.7, NOISeq = 17.1, baySeq = 6.5
Simes = 11.4, Union = 5, Intersection = 15.8
PANDORA = 6.3, Fisher = 5.4, Whitlock = 11.5
DESeq = 3.3, edgeR = 3.9, voom = 7.7
NBPSeq = 3.3, NOISeq = 7.4, baySeq = 4.7
Simes = 3.8, Union = 2.3, Intersection = 6.9
PANDORA = 2.9, Fisher = 2.5, Whitlock = 4.6
DESeq = 6.2, edgeR = 6.2, voom = 8.9
NBPSeq = 5.6, NOISeq = 9.1, baySeq = 9.8
Simes = 7.8, Union = 4.2, Intersection = 11.6
PANDORA = 4.8, Fisher = 4.4, Whitlock = 7.8
DESeq = 2.9, edgeR = 3.5, voom = 5.7
NBPSeq = 2.4, NOISeq = 4.4, baySeq = 6.6
Simes = 4.2, Union = 2.2, Intersection = 7.1
PANDORA = 2.7, Fisher = 2.3, Whitlock = 3.4
DESeq = 2, edgeR = 2.2, voom = 2.7
NBPSeq = 1.6, NOISeq = 4.5, baySeq = 5.3
Simes = 2.5, Union = 1.5, Intersection = 4.2
PANDORA = 1.7, Fisher = 1.5, Whitlock = 2.3
DESeq = 1.1, edgeR = 1.2, voom = 1.5
NBPSeq = 1.3, NOISeq = 1.4, baySeq = 3.9
Simes = 1.4, Union = 1, Intersection = 3.2
PANDORA = 1.1, Fisher = 1, Whitlock = 1.3
DESeq = 1.5, edgeR = 2, voom = 3.1
NBPSeq = 2, NOISeq = 3.4, baySeq = 11.5
Simes = 3.3, Union = 1.3, Intersection = 8
PANDORA = 1.6, Fisher = 1.3, Whitlock = 2.9
DESeq = 1.1, edgeR = 1.5, voom = 1.6
NBPSeq = 1.1, NOISeq = 1.6, baySeq = 9.9
Simes = 1.8, Union = 1, Intersection = 7
PANDORA = 1.1, Fisher = 1, Whitlock = 1.4
DESeq = 5.7, edgeR = 5.6, voom = 13.3
NBPSeq = 5.4, NOISeq = 8.2, baySeq = 7.6
Simes = 6.6, Union = 5.2, Intersection = 8.6
PANDORA = 6.4, Fisher = 4.5, Whitlock = 6
DESeq = 2.9, edgeR = 3.7, voom = 8.9
NBPSeq = 2.7, NOISeq = 4.5, baySeq = 5
Simes = 3.5, Union = 2.4, Intersection = 5.6
PANDORA = 3.3, Fisher = 2.3, Whitlock = 3.2
Algorithm
baySeq
Intersection
Simes
DESeq
NBPSeq
Union
edgeR
NOISeq
voom
Fisher
PANDORA
Whitlock

Figure S5: False Negative Curves using each package's specific normalization and unadjusted p-values

FNCs generated with simulated data for each statistical test and each p-value combination method supported by metaseqR for five organisms and two simulation configurations, using the normalization method suggested by each individual package instead of EDASeq. The results for each organism and simulation configuration can be distinguished by the right and top side titles of each panel, referring to the organism from which simulation parameters are estimated and the simulation configuration respectively. The performance value according to the AUFC is displayed in each panel next to the curves (Supplementary methods). The lowest possible value is 1, indicating no false negative hits among the first 2000 ranked genes according to increasing statistical significance (from higher to lower p-values). Dashed lines represent individual tests whereas solid lines represent p-value combinations and the thicker solid line highlights the FNC produced by PANDORA. limma voom and baySeq are constantly among the bottom performing algorithms in terms of missing true positive hits. PANDORA is quite stable, changing slightly performance positions according to simulation configuration and organism. As expected, the higher number of replicates increases the performance of all algorithms in terms of losing true positives (right panels). The curves as well as the performance values are constructed and calculated across ten simulations for each organism and simulation configuration, respectively.

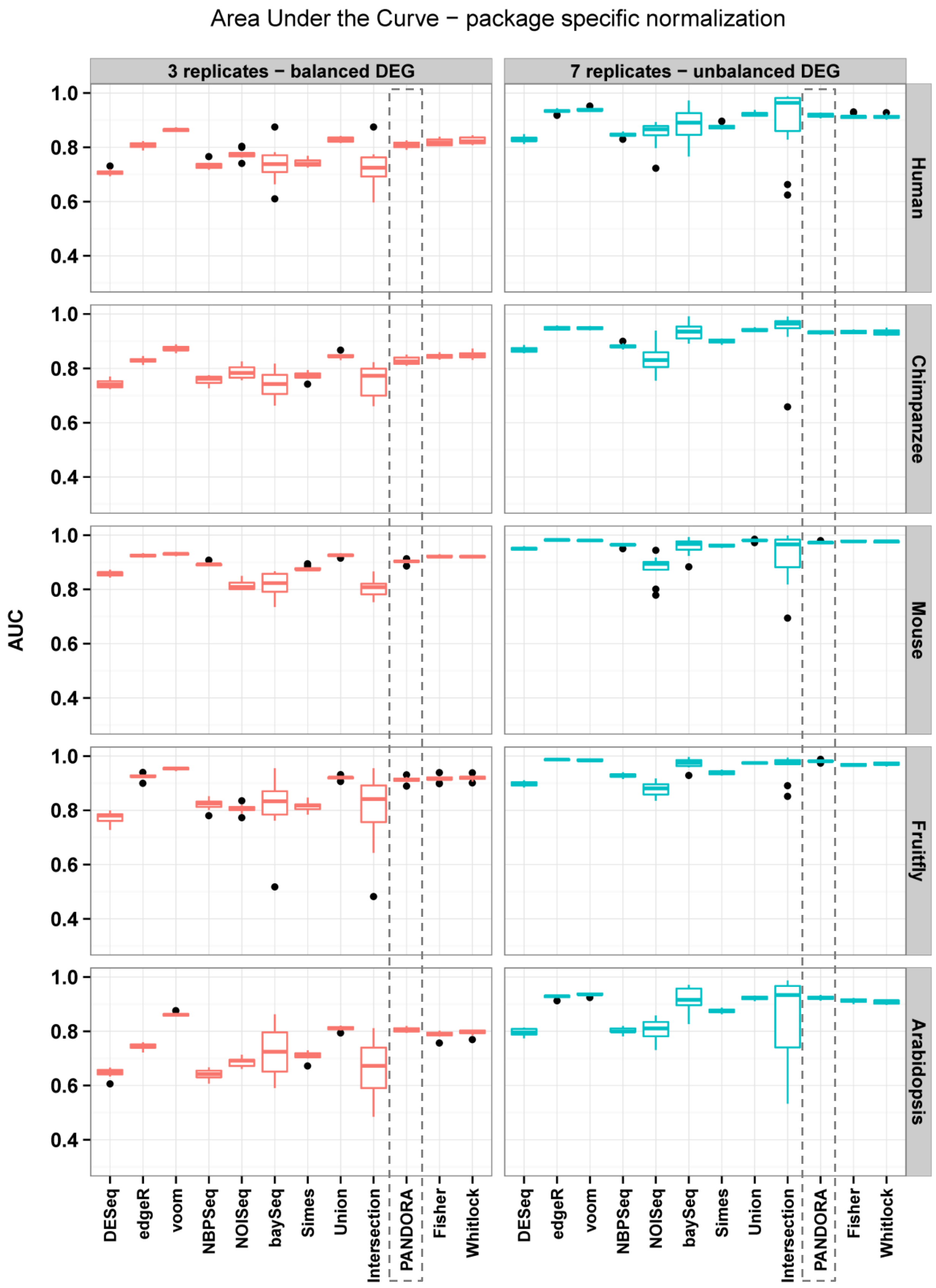
Area Under the Curve − package specific normalization
3 replicates − balanced DEG
7 replicates − unbalanced DEG
AUC
1.0
0.8
0.6
0.4
Human
Chimpanzee
Mouse
Fruitfly
Arabidopsis
DESeq
edgeR
voom
NBPSeq
NOISeq
baySeq
Simes
Union
Intersection
PANDORA
Fisher
Whitlock

Figure S6: Receiver Operating Characteristic analysis using each package's specific normalization and unadjusted p-values

The boxplots depict the summarized areas under the ROC curve across ten simulations for each organism and for each simulation configuration, using each package's specific normalization instead of EDASeq. The results for each organism and simulation configuration can be distinguished by the right and top side titles of each panel, referring to the organism from which simulation parameters are estimated and the simulation configuration, respectively. The dashed rectangle highlights the PANDORA method results. Each statistical test's and each p-value combination method's performance trends are similar, apart from the Mouse case, where the performance is increased in both simulation configurations. The impact of incorporating more replicates to the simulation is evident, as most methods achieve area under the curve values closer to 1 in all cases. PANDORA performs very similar to the highest scoring algorithms according to the area under the ROC curves. The performance trends do not change as compared to assessment with EDASeq normalization.

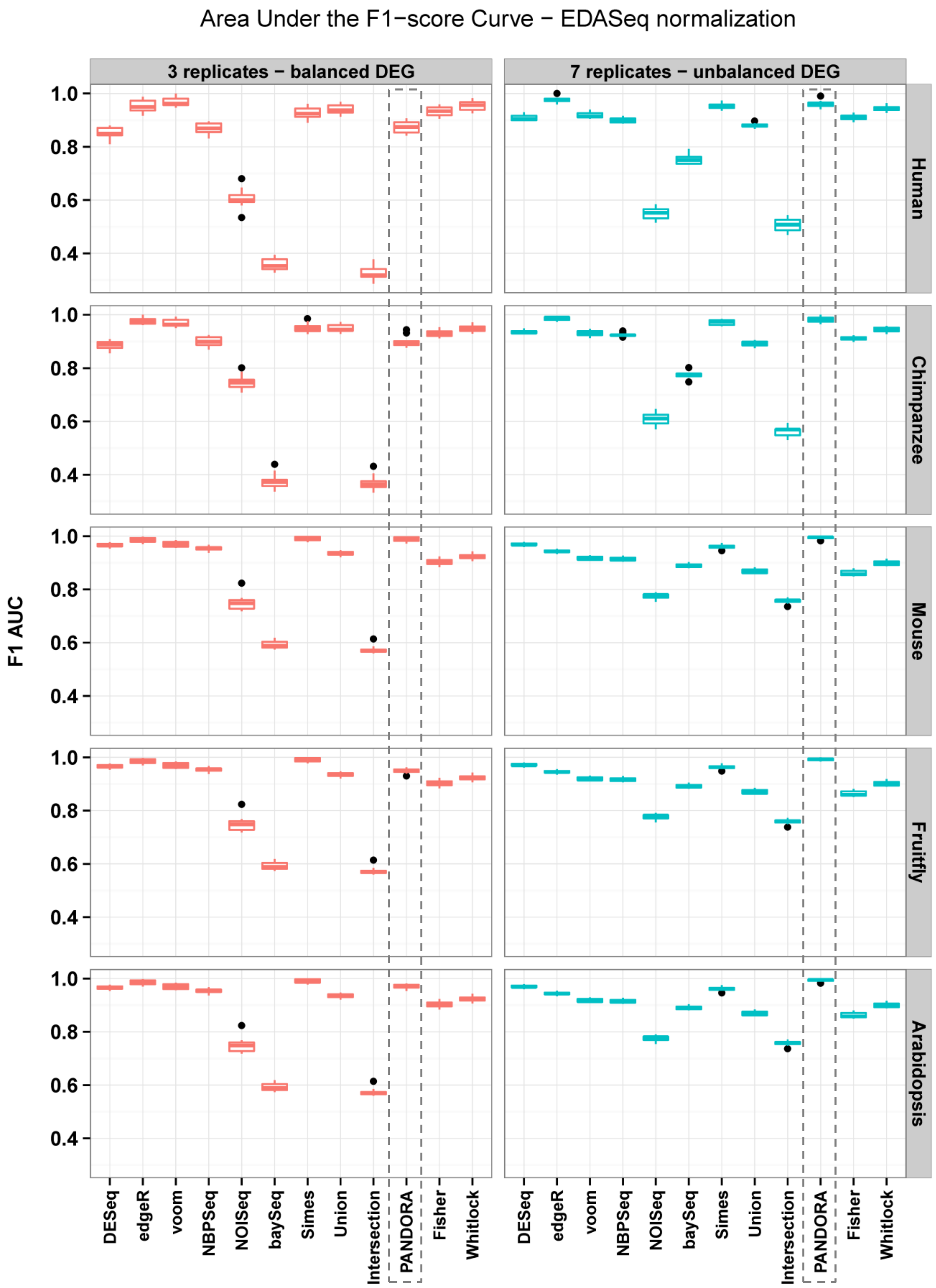
Area Under the F1−score Curve − EDASeq normalization
3 replicates − balanced DEG
7 replicates − unbalanced DEG
Human
Chimpanzee
Mouse
Fruitfly
Arabidopsis
F1 AUC
1.0
0.8
0.6
0.4
DESeq
edgeR
voom
NBPSeq
NOISeq
baySeq
Simes
Union
Intersection
PANDORA
Fisher
Whitlock

Figure S7: Areas under the F_1-score curves using EDASeq normalization and unadjusted p-values

The boxplots depict the summarized areas under the F_1-score curve across ten simulations for each organism and for each simulation configuration. The results for each organism and simulation configuration can be distinguished by the right and top side titles of each panel, referring to the organism from which simulation parameters are estimated and the simulation configuration respectively. The dashed rectangle highlights the PANDORA method which shows the highest quantified area under the F_1-score curve for half the cases. In all other cases it is very close to the best performing method. This indicates that while PANDORA may not achieve F_1-score optimization while looking at the top ranked genes (contributing to the construction of an F_1-score curve), nevertheless, it achieves this optimization when looking at finalized gene lists as depicted in Figure 4 in main text.

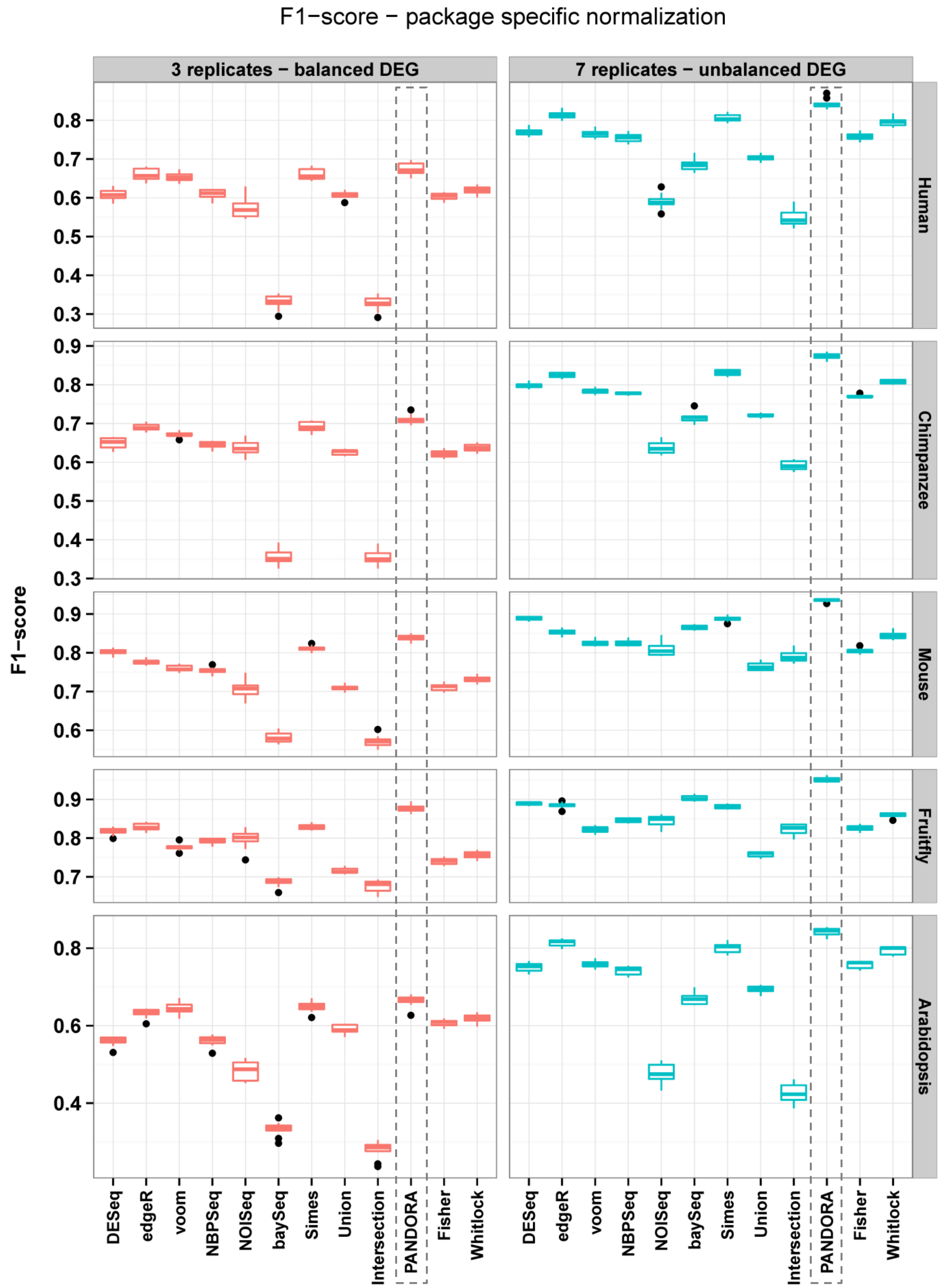

F1−score − package specific normalization
3 replicates − balanced DEG
7 replicates − unbalanced DEG
F1−score
Human
Chimpanzee
Mouse
Fruitfly
Arabidopsis
DESeq
edgeR
voom
NBPSeq
NOISeq
baySeq
Simes
Union
Intersection
PANDORA
Fisher
Whitlock

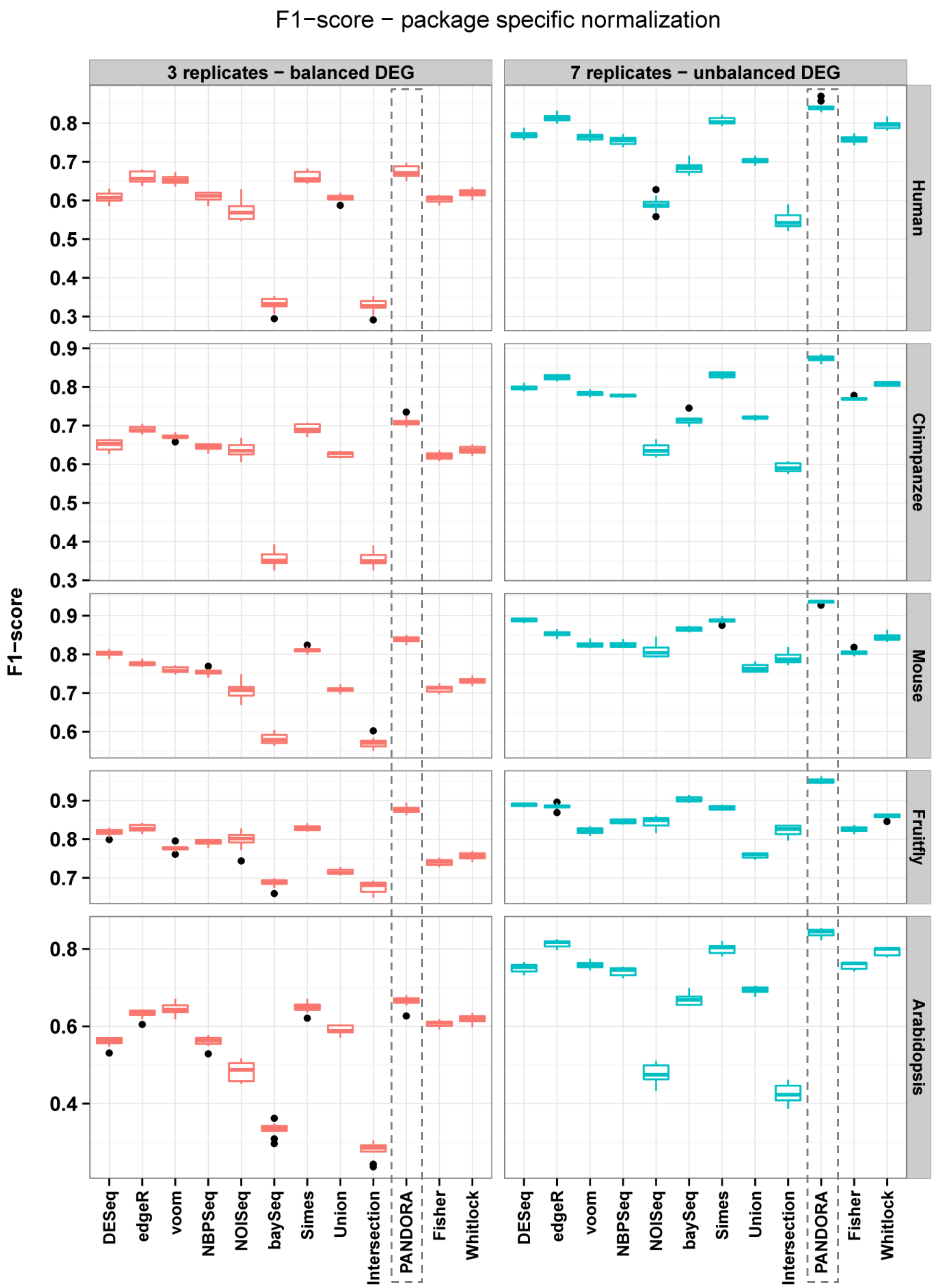
F1−score − package specific normalization
3 replicates − balanced DEG
7 replicates − unbalanced DEG
F1−score
Human
Chimpanzee
Mouse
Fruitfly
Arabidopsis
DESeq
edgeR
voom
NBPSeq
NOISeq
baySeq
Simes
Union
Intersection
PANDORA
Fisher
Whitlock

Figure S8: Analysis of the F_1-score using each package's specific normalization and unadjusted p-values

The boxplots summarize the F_1-score across ten simulations for each organism and for each simulation configuration, when using each package's specific normalization instead of EDASeq. The F_1-scores for each organism and simulation configuration can be distinguished by the right and top side titles of each panel, referring to the organism from which simulation parameters are estimated and the simulation configuration respectively. The dashed rectangle highlights the PANDORA method results. The prevalence of PANDORA is evident in all cases. Intersection and baySeq constantly show the lowest F_1-score. F_1-scores are calculated using the final gene lists returned by each method at a p-value cutoff of 0.05. The general performance trends do not change when compared to the case of using EDASeq normalization.

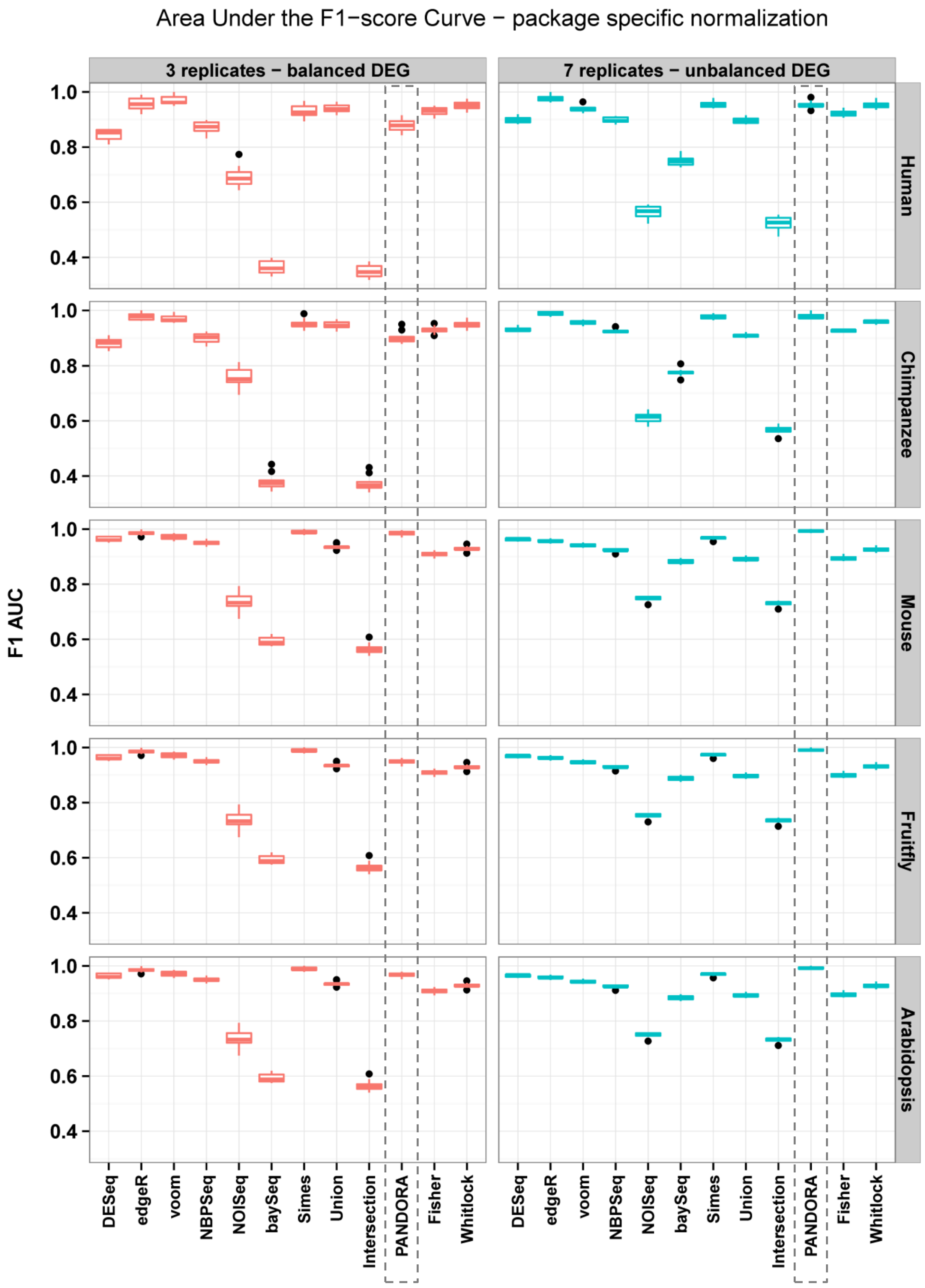
Area Under the F1−score Curve − package specific normalization
3 replicates − balanced DEG
7 replicates − unbalanced DEG
F1 AUC
1.0
0.8
0.6
0.4
Human
Chimpanzee
Mouse
Fruitfly
Arabidopsis
DESeq
edgeR
voom
NBPSeq
NOISeq
baySeq
Simes
Union
Intersection
PANDORA
Fisher
Whitlock

Figure S9: Areas under normalized F_1-score curves using each package's specific normalization and unadjusted p-values

The boxplots depict the summarized areas under the normalized F_1-score curve across ten simulations for each organism and for each simulation configuration when using each package's specific normalization instead of EDASeq. The results for each organism and simulation configuration can be distinguished by the right and top side titles of each panel, referring to the organism from which simulation parameters are estimated and the simulation configuration, respectively. The dashed rectangle highlights the PANDORA method results. PANDORA shows the highest quantified area under the F_1-score curve for half the cases. In all other cases it is very close to the best performing method. This indicates that while PANDORA may not achieve F_1-score optimization while looking at the top ranked genes (contributing to the construction of an F_1-score curve); nevertheless, it achieves this optimization when looking at finalized gene lists, as depicted in Figure S8. The general performance trends do not change when compared to the case of using EDASeq normalization.

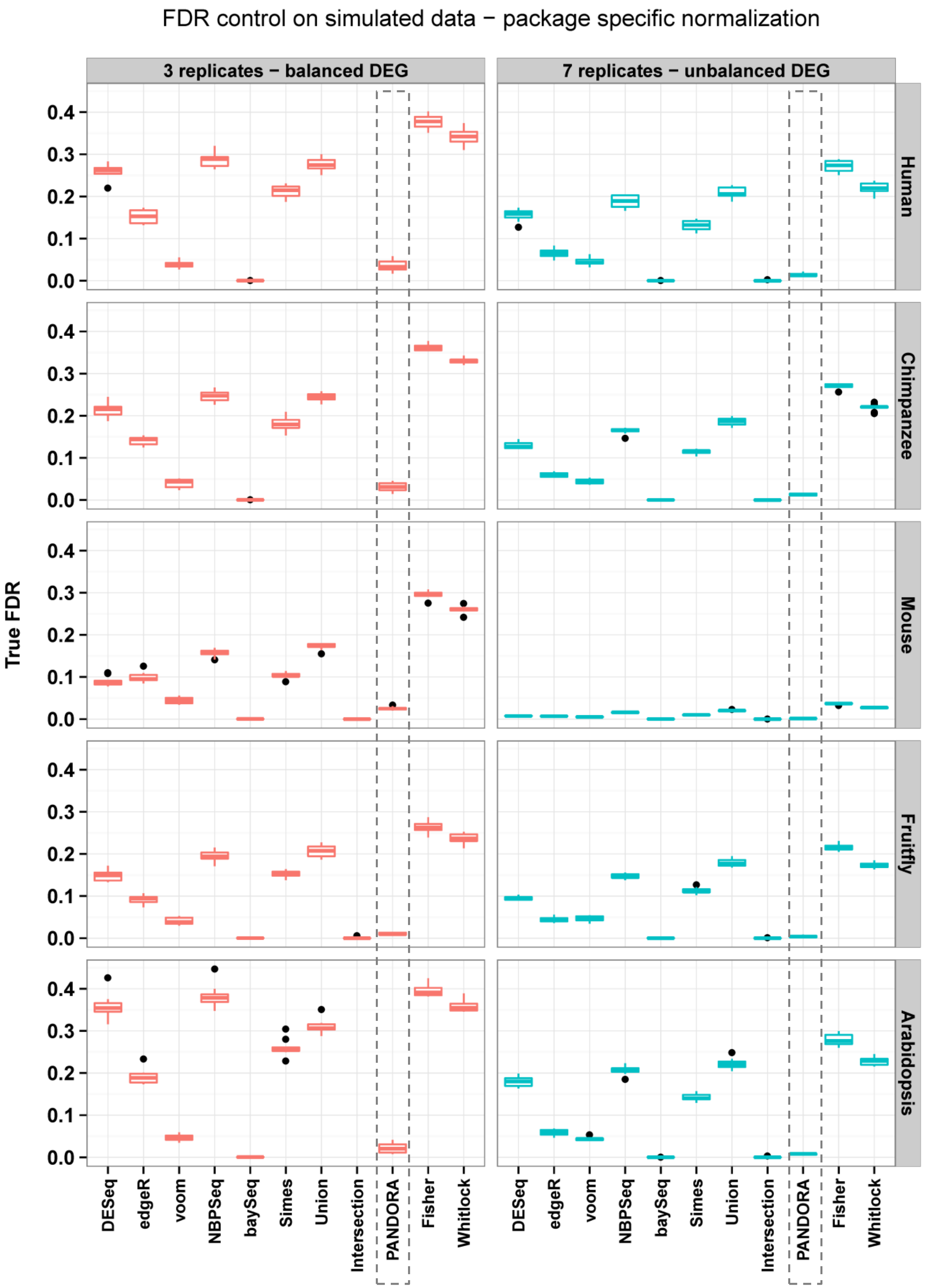
FDR control on simulated data − package specific normalization
3 replicates − balanced DEG
7 replicates − unbalanced DEG
True FDR
0.0
0.1
0.2
0.3
0.4
Human
Chimpanzee
Mouse
Fruitfly
Arabidopsis
DESeq
edgeR
voom
NBPSeq
baySeq
Simes
Union
Intersection
PANDORA
Fisher
Whitlock

Figure S10: Analysis of true False Discovery Rates using each package's specific normalization

The boxplots summarize the true FDRs across ten simulations for each organism and for each simulation configuration using each package's specific normalization. The true FDRs for each organism and simulation configuration can be distinguished by the right and top side titles of each panel, referring to the organism from which simulation parameters are estimated and the simulation configuration, respectively. The true FDRs are estimated as the ratio of false positive hits determined by the a priori knowledge of differentially expressed genes for each simulation, to the respective number of genes passing a Benjamini-Hochberg FDR of 5%. The dashed rectangle highlights the PANDORA method results. From the individual statistical tests, NBPSeq constantly shows the highest true FDR, indicating poor performance regarding false discovery control, and baySeq the lowest. From the p-value combination methods, Fisher and Whitlock show the highest true FDRs. PANDORA is the next best combination method to the Intersection (which is expected to have a good FDR because of stringency) in terms of FDR control. These performance trends are the same when using EDASeq normalization.

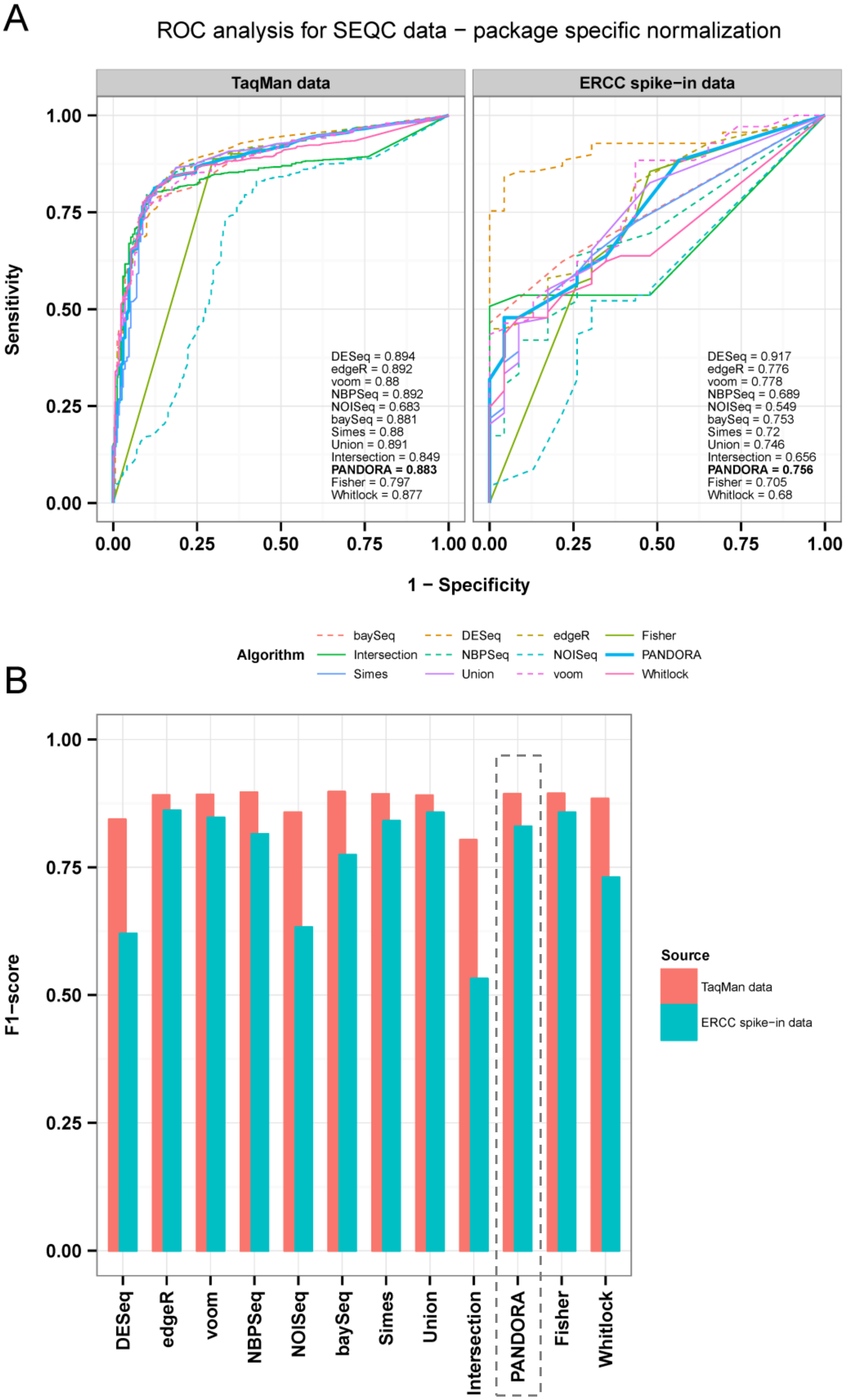

A
ROC analysis for SEQC data − package specific normalization
TaqMan data
ERCC spike−in data
Sensitivity
1 − Specificity
DESeq = 0.894
edgeR = 0.892
voom = 0.88
NBPSeq = 0.892
NOISeq = 0.683
baySeq = 0.881
Simes = 0.88
Union = 0.891
Intersection = 0.849
PANDORA = 0.883
Fisher = 0.797
Whitlock = 0.877
DESeq = 0.917
edgeR = 0.776
voom = 0.778
NBPSeq = 0.689
NOISeq = 0.549
baySeq = 0.753
Simes = 0.72
Union = 0.746
Intersection = 0.656
PANDORA = 0.756
Fisher = 0.705
Whitlock = 0.68
Algorithm
baySeq
DESeq
edgeR
Fisher
Intersection
NBPSeq
NOISeq
PANDORA
Simes
Union
voom
Whitlock
B
F1−score
Source
TaqMan data
ERCC spike−in data
DESeq
edgeR
voom
NBPSeq
NOISeq
baySeq
Simes
Union
Intersection
PANDORA
Fisher
Whitlock

Figure S11: Receiver Operating Characteristic and F_1-score analysis for SEQC data using each package's specific normalization and unadjusted p-values

(A) ROC curves calculated using TaqMan data (left panel) and ERCC spike-in data (right panel), using each package's specific normalization method for data processing instead of EDASeq. Dashed lines represent individual tests whereas solid lines represent p-value combinations and the thicker solid line highlights the ROC produced by PANDORA. Actual area under the curve values are shown on the right of each panel. In the cases of TaqMan data, DESeq performs slightly better than the other methods, whereas in the case of ERCC spike-in data, it performs considerably better. The performance of all other methods drops in the case of ERCC spike-ins, and particularly these of NOISeq and Fisher. The performance of PANDORA lies in the middle of area under the curve values in both cases, indicating good performance.

(B) F_1-scores using TaqMan data (red bars) and ERCC spike-in data (green bars), using each package's specific normalization method for data processing instead of EDASeq. The dashed rectangle highlights the PANDORA method results. All methods apart from DESeq, NOISeq and Intersection achieve similar F_1-score levels when looking only at the TaqMan gene list (not the total putative DEG list of the SEQC data). When using ERCC spike-in data, baySeq, Simes and, Whitlock also perform poorly. PANDORA is again in the middle in both cases, indicating adequate performance.

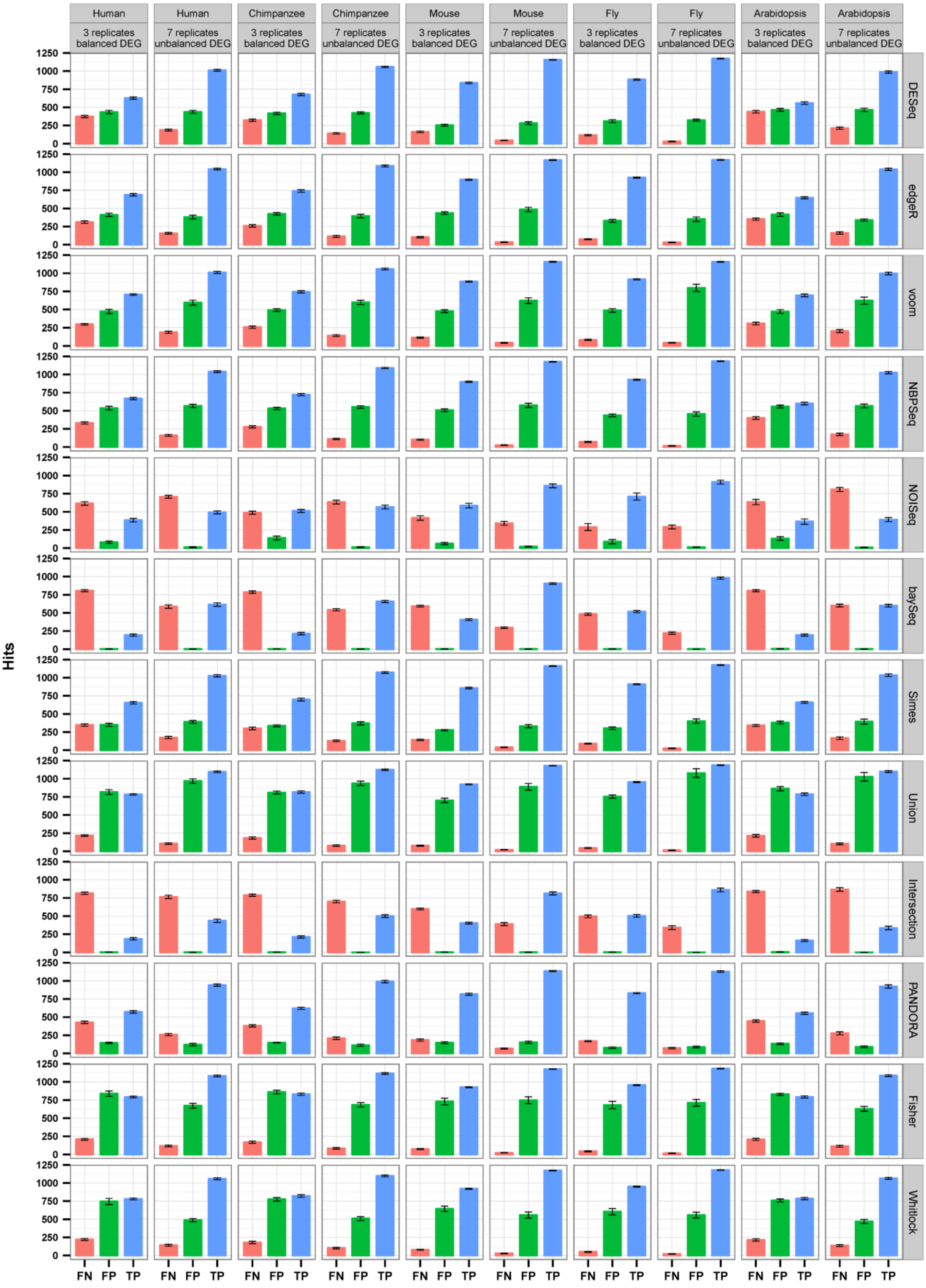

Human
3 replicates balanced DEG
Human
7 replicates unbalanced DEG
Chimpanzee
3 replicates balanced DEG
Chimpanzee
7 replicates unbalanced DEG
Mouse
3 replicates balanced DEG
Mouse
7 replicates unbalanced DEG
Fly
3 replicates balanced DEG
Fly
7 replicates unbalanced DEG
Arabidopsis
3 replicates balanced DEG
Arabidopsis
7 replicates unbalanced DEG
DESeq
edgeR
voom
NBPSeq
NOISeq
baySeq
Simes
Union
Intersection
PANDORA
Fisher
Whitlock
Hits
0
250
500
750
1000
1250
FN
FP
TP

Figure S12: False Negative, False Positive and True positive hits for all simulated data

FNs, FPs and TPs for six statistical tests for RNA-Seq data and seven p-value combination methods, using EDASeq normalization for data processing. Numbers were calculated with unadjusted p-values. The grid of bar graphs decomposes the FDT values reported in Figure 4 in the main text to its components so that actual numbers of FNs, FPs and TPs can be seen. PANDORA consistently shows large numbers of TPs and small numbers of FPs and FNs. The values for each organism, simulation configuration and statistical method can be distinguished by the right and top side titles of each panel, referring to the organism from which simulation parameters are estimated, the simulation configuration and the statistical method, respectively.

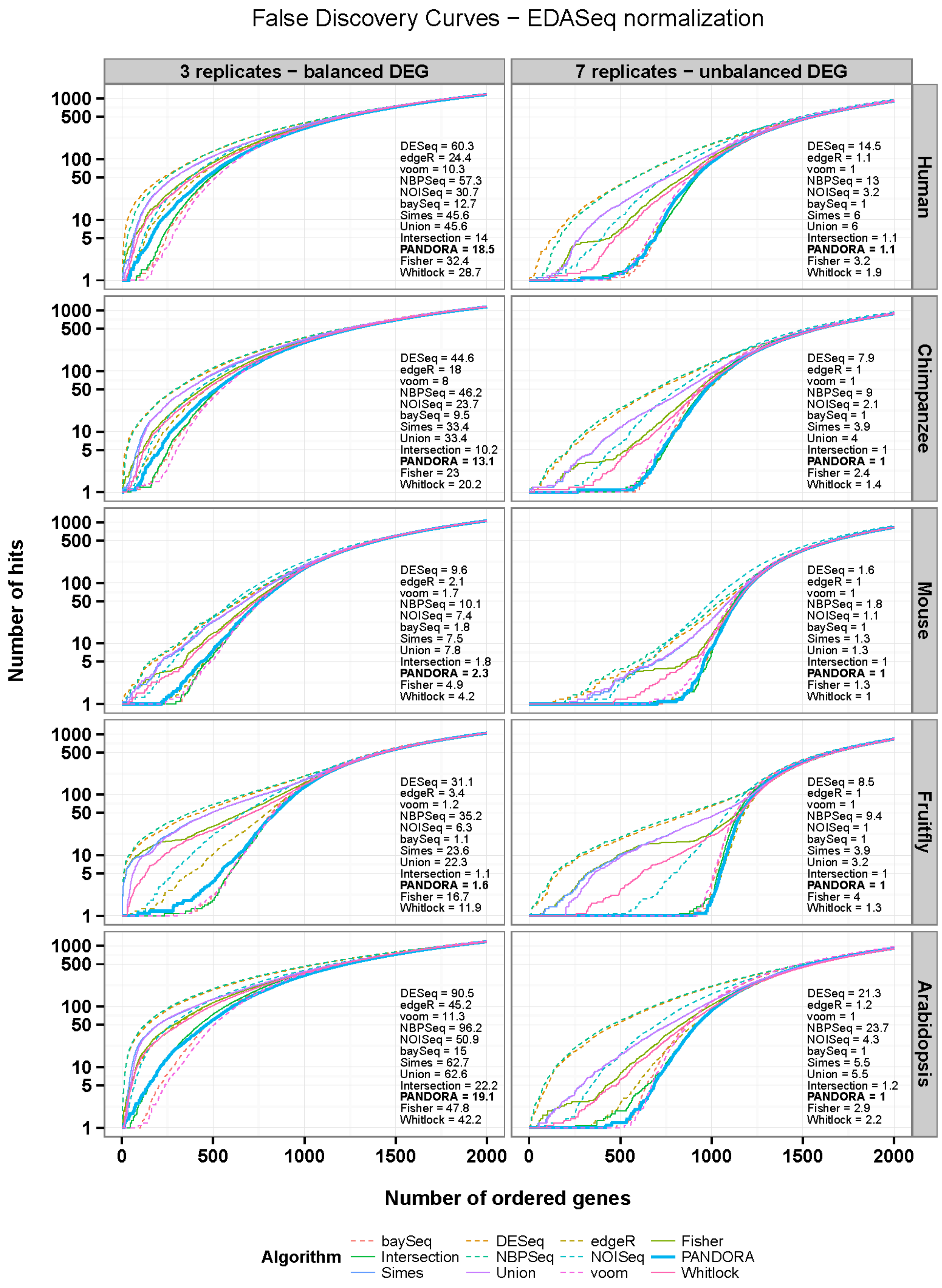
False Discovery Curves − EDASeq normalization
3 replicates − balanced DEG
7 replicates − unbalanced DEG
Human
Chimpanzee
Mouse
Fruitfly
Arabidopsis
Number of hits
Number of ordered genes
1
5
10
50
100
500
1000
0
500
1000
1500
2000
DESeq = 60.3
edgeR = 24.4
voom = 10.3
NBPSeq = 57.3
NOISeq = 30.7
baySeq = 12.7
Simes = 45.6
Union = 45.6
Intersection = 14
PANDORA = 18.5
Fisher = 32.4
Whitlock = 28.7
DESeq = 14.5
edgeR = 1.1
voom = 1
NBPSeq = 13
NOISeq = 3.2
baySeq = 1
Simes = 6
Union = 6
Intersection = 1.1
PANDORA = 1.1
Fisher = 3.2
Whitlock = 1.9
DESeq = 44.6
edgeR = 18
voom = 8
NBPSeq = 46.2
NOISeq = 23.7
baySeq = 9.5
Simes = 33.4
Union = 33.4
Intersection = 10.2
PANDORA = 13.1
Fisher = 23
Whitlock = 20.2
DESeq = 7.9
edgeR = 1
voom = 1
NBPSeq = 9
NOISeq = 2.1
baySeq = 1
Simes = 3.9
Union = 4
Intersection = 1
PANDORA = 1
Fisher = 2.4
Whitlock = 1.4
DESeq = 9.6
edgeR = 2.1
voom = 1.7
NBPSeq = 10.1
NOISeq = 7.4
baySeq = 1.8
Simes = 7.5
Union = 7.8
Intersection = 1.8
PANDORA = 2.3
Fisher = 4.9
Whitlock = 4.2
DESeq = 1.6
edgeR = 1
voom = 1
NBPSeq = 1.8
NOISeq = 1.1
baySeq = 1
Simes = 1.3
Union = 1.3
Intersection = 1
PANDORA = 1
Fisher = 1.3
Whitlock = 1
DESeq = 31.1
edgeR = 3.4
voom = 1.2
NBPSeq = 35.2
NOISeq = 6.3
baySeq = 1.1
Simes = 23.6
Union = 22.3
Intersection = 1.1
PANDORA = 1.6
Fisher = 16.7
Whitlock = 11.9
DESeq = 8.5
edgeR = 1
voom = 1
NBPSeq = 9.4
NOISeq = 1
baySeq = 1
Simes = 3.9
Union = 3.2
Intersection = 1
PANDORA = 1
Fisher = 4
Whitlock = 1.3
DESeq = 90.5
edgeR = 45.2
voom = 11.3
NBPSeq = 96.2
NOISeq = 50.9
baySeq = 15
Simes = 62.7
Union = 62.6
Intersection = 22.2
PANDORA = 19.1
Fisher = 47.8
Whitlock = 42.2
DESeq = 21.3
edgeR = 1.2
voom = 1
NBPSeq = 23.7
NOISeq = 4.3
baySeq = 1
Simes = 5.5
Union = 5.5
Intersection = 1.2
PANDORA = 1
Fisher = 2.9
Whitlock = 2.2
Algorithm
baySeq
DESeq
edgeR
Fisher
Intersection
NBPSeq
NOISeq
PANDORA
Simes
Union
voom
Whitlock

Figure S13: False Discovery Curves using EDASeq normalization and adjusted p-values

FDCs generated with simulated data for each statistical test and each p-value combination method supported by metaseqR for five organisms and two simulation configurations, using Benjamini-Hochberg adjusted p-values. The results for each organism and simulation configuration can be distinguished by the right and top side titles of each panel, referring to the organism from which simulation parameters are estimated and the simulation configuration, respectively. The performance value according to the AUFC is displayed in each panel next to the curves (Supplementary methods). The lowest possible value is 1, indicating no false discoveries among the first 500 top ranked genes according to statistical significance. Dashed lines represent individual tests whereas solid lines represent p-value combinations and the thicker solid line highlights the FDC produced by PANDORA. limma voom is constantly among the best performing algorithms and PANDORA among the best methods in general. As expected, higher numbers of replicates increases the accuracy and performance of all algorithms (right panels). The curves as well as the AUFC values are constructed and calculated across ten simulations for each organism and simulation configuration, respectively. The performance trends are the same as when using unadjusted p-values.

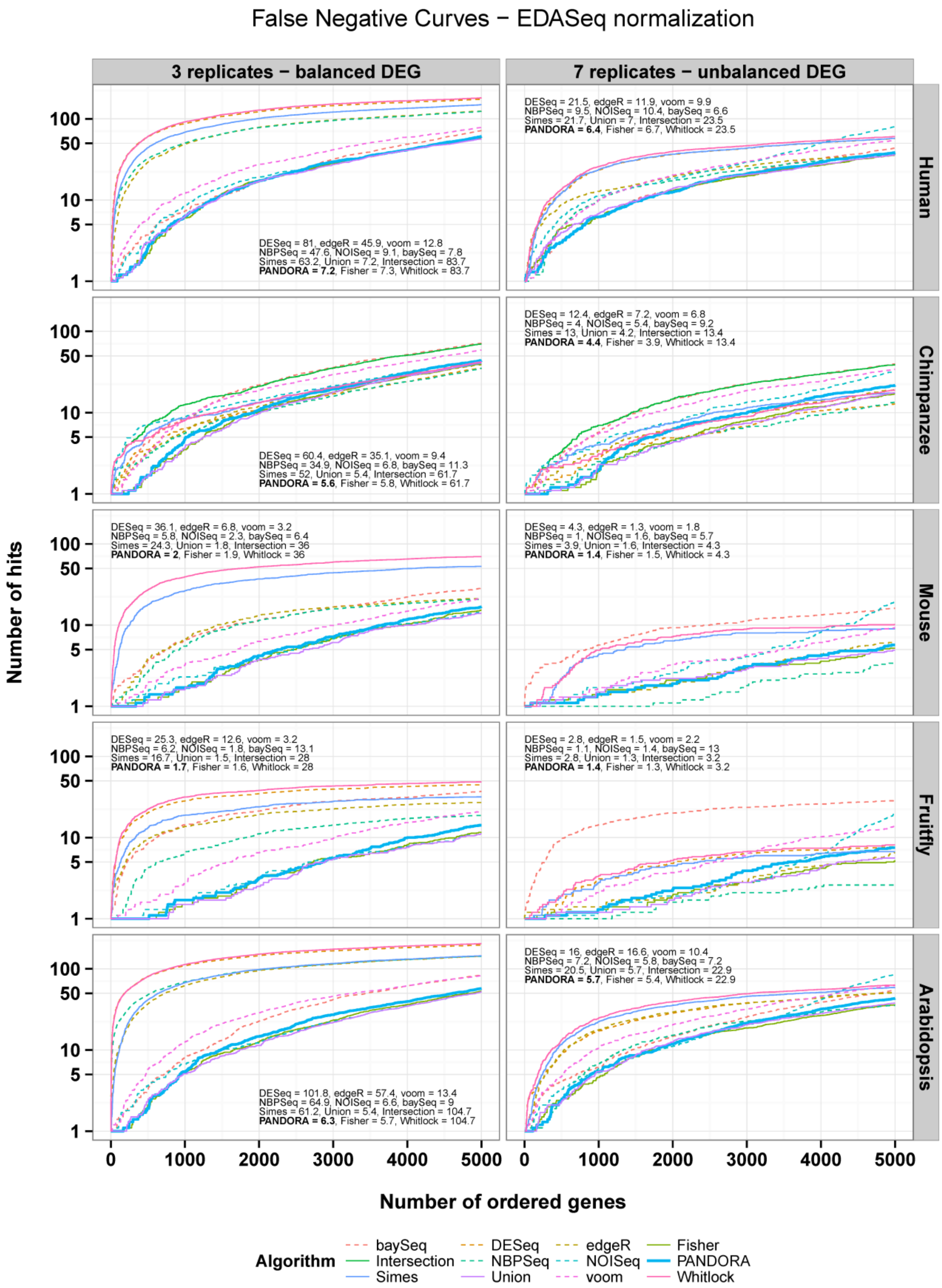
False Negative Curves − EDASeq normalization
3 replicates − balanced DEG
7 replicates − unbalanced DEG
Human
Chimpanzee
Mouse
Fruitfly
Arabidopsis
Number of hits
Number of ordered genes
DESeq = 81, edgeR = 45.9, voom = 12.8
NBPSeq = 47.6, NOISeq = 9.1, baySeq = 7.8
Simes = 63.2, Union = 7.2, Intersection = 83.7
PANDORA = 7.2, Fisher = 7.3, Whitlock = 83.7
DESeq = 21.5, edgeR = 11.9, voom = 9.9
NBPSeq = 9.5, NOISeq = 10.4, baySeq = 6.6
Simes = 21.7, Union = 7, Intersection = 23.5
PANDORA = 6.4, Fisher = 6.7, Whitlock = 23.5
DESeq = 60.4, edgeR = 35.1, voom = 9.4
NBPSeq = 34.9, NOISeq = 6.8, baySeq = 11.3
Simes = 52, Union = 5.4, Intersection = 61.7
PANDORA = 5.6, Fisher = 5.8, Whitlock = 61.7
DESeq = 12.4, edgeR = 7.2, voom = 6.8
NBPSeq = 4, NOISeq = 5.4, baySeq = 9.2
Simes = 13, Union = 4.2, Intersection = 13.4
PANDORA = 4.4, Fisher = 3.9, Whitlock = 13.4
DESeq = 36.1, edgeR = 6.8, voom = 3.2
NBPSeq = 5.8, NOISeq = 2.3, baySeq = 6.4
Simes = 24.3, Union = 1.8, Intersection = 36
PANDORA = 2, Fisher = 1.9, Whitlock = 36
DESeq = 4.3, edgeR = 1.3, voom = 1.8
NBPSeq = 1, NOISeq = 1.6, baySeq = 5.7
Simes = 3.9, Union = 1.6, Intersection = 4.3
PANDORA = 1.4, Fisher = 1.5, Whitlock = 4.3
DESeq = 25.3, edgeR = 12.6, voom = 3.2
NBPSeq = 6.2, NOISeq = 1.8, baySeq = 13.1
Simes = 16.7, Union = 1.5, Intersection = 28
PANDORA = 1.7, Fisher = 1.6, Whitlock = 28
DESeq = 2.8, edgeR = 1.5, voom = 2.2
NBPSeq = 1.1, NOISeq = 1.4, baySeq = 13
Simes = 2.8, Union = 1.3, Intersection = 3.2
PANDORA = 1.4, Fisher = 1.3, Whitlock = 3.2
DESeq = 101.8, edgeR = 57.4, voom = 13.4
NBPSeq = 64.9, NOISeq = 6.6, baySeq = 9
Simes = 61.2, Union = 5.4, Intersection = 104.7
PANDORA = 6.3, Fisher = 5.7, Whitlock = 104.7
DESeq = 16, edgeR = 16.6, voom = 10.4
NBPSeq = 7.2, NOISeq = 5.8, baySeq = 7.2
Simes = 20.5, Union = 5.7, Intersection = 22.9
PANDORA = 5.7, Fisher = 5.4, Whitlock = 22.9
Algorithm
baySeq
Intersection
Simes
DESeq
NBPSeq
Union
edgeR
NOISeq
voom
Fisher
PANDORA
Whitlock

Figure S14: False Negative Curves using EDASeq normalization and adjusted p-values

FNCs generated with simulated data for each statistical test and each p-value combination method supported by metaseqR for five organisms and two simulation configurations, using Benjamini-Hochberg adjusted p-values. The results for each organism and simulation configuration can be distinguished by the right and top side titles of each panel, referring to the organism from which simulation parameters are estimated and the simulation configuration respectively. The performance value according to the AUFC is displayed in each panel next to the curves (Supplementary methods). The lowest possible value is 1, indicating no false negative hits among the first 2000 ranked genes according to increasing statistical significance (from higher to lower p-values). Dashed lines represent individual tests whereas solid lines represent p-value combinations and the thicker solid line highlights the FNC produced by PANDORA. The performance of statistical tests where multiple testing correction can be applied drops considerably, with limma voom showing the most moderate drop. PANDORA is less affected. As expected, higher numbers of replicates partially remedies the aforementioned performance drop. The curves as well as the AUFC values are constructed and calculated respectively across ten simulations for each organism and simulation configuration.

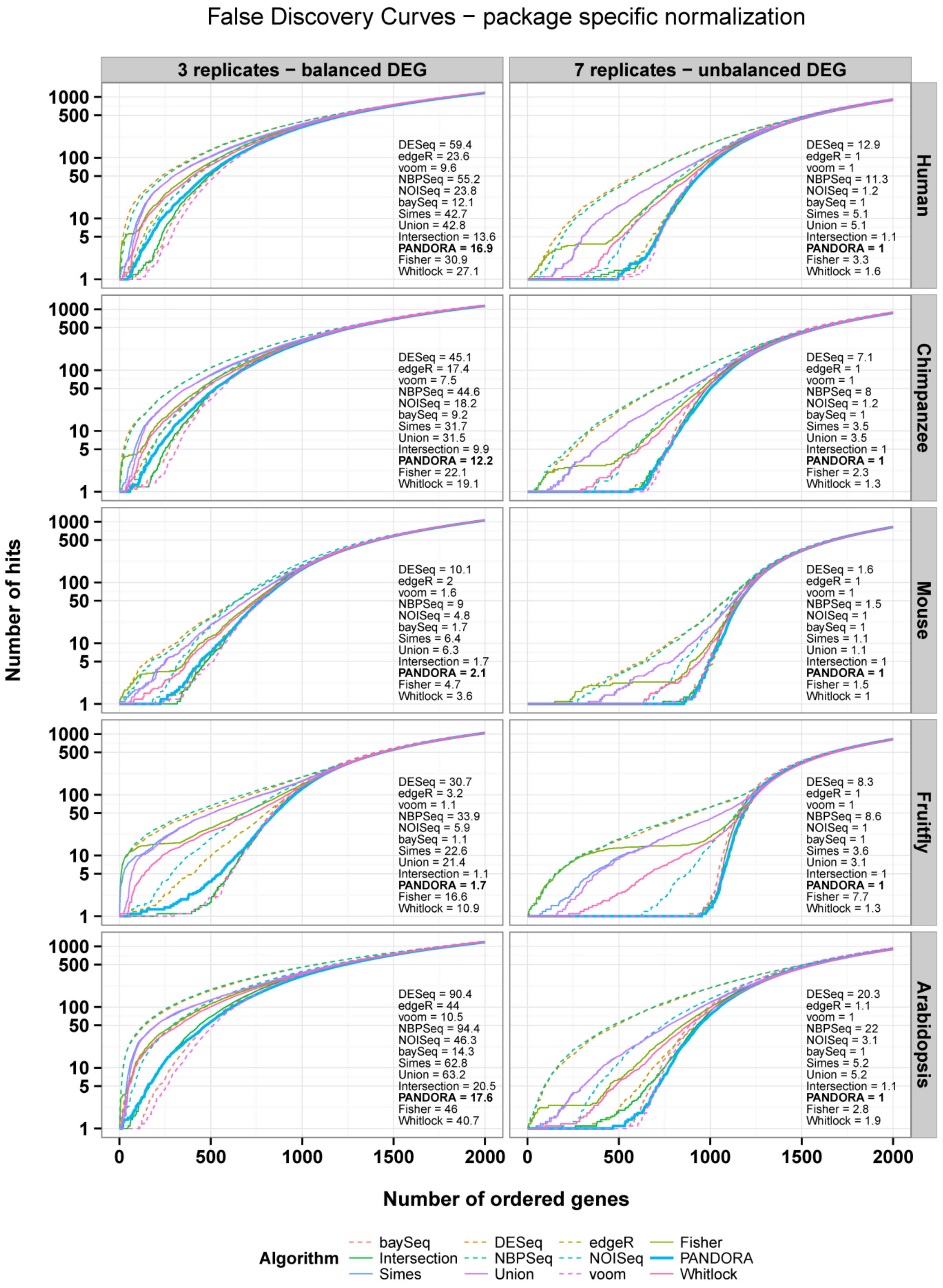
False Discovery Curves − package specific normalization
3 replicates − balanced DEG
7 replicates − unbalanced DEG
Human
Chimpanzee
Mouse
Fruitfly
Arabidopsis
Number of hits
Number of ordered genes
DESeq = 59.4
edgeR = 23.6
voom = 9.6
NBPSeq = 55.2
NOISeq = 23.8
baySeq = 12.1
Simes = 42.7
Union = 42.8
Intersection = 13.6
PANDORA = 16.9
Fisher = 30.9
Whitlock = 27.1
DESeq = 12.9
edgeR = 1
voom = 1
NBPSeq = 11.3
NOISeq = 1.2
baySeq = 1
Simes = 5.1
Union = 5.1
Intersection = 1.1
PANDORA = 1
Fisher = 3.3
Whitlock = 1.6
DESeq = 45.1
edgeR = 17.4
voom = 7.5
NBPSeq = 44.6
NOISeq = 18.2
baySeq = 9.2
Simes = 31.7
Union = 31.5
Intersection = 9.9
PANDORA = 12.2
Fisher = 22.1
Whitlock = 19.1
DESeq = 7.1
edgeR = 1
voom = 1
NBPSeq = 8
NOISeq = 1.2
baySeq = 1
Simes = 3.5
Union = 3.5
Intersection = 1
PANDORA = 1
Fisher = 2.3
Whitlock = 1.3
DESeq = 10.1
edgeR = 2
voom = 1.6
NBPSeq = 9
NOISeq = 4.8
baySeq = 1.7
Simes = 6.4
Union = 6.3
Intersection = 1.7
PANDORA = 2.1
Fisher = 4.7
Whitlock = 3.6
DESeq = 1.6
edgeR = 1
voom = 1
NBPSeq = 1.5
NOISeq = 1
baySeq = 1
Simes = 1.1
Union = 1.1
Intersection = 1
PANDORA = 1
Fisher = 1.5
Whitlock = 1
DESeq = 30.7
edgeR = 3.2
voom = 1.1
NBPSeq = 33.9
NOISeq = 5.9
baySeq = 1.1
Simes = 22.6
Union = 21.4
Intersection = 1.1
PANDORA = 1.7
Fisher = 16.6
Whitlock = 10.9
DESeq = 8.3
edgeR = 1
voom = 1
NBPSeq = 8.6
NOISeq = 1
baySeq = 1
Simes = 3.6
Union = 3.1
Intersection = 1
PANDORA = 1
Fisher = 7.7
Whitlock = 1.3
DESeq = 90.4
edgeR = 44
voom = 10.5
NBPSeq = 94.4
NOISeq = 46.3
baySeq = 14.3
Simes = 62.8
Union = 63.2
Intersection = 20.5
PANDORA = 17.6
Fisher = 46
Whitlock = 40.7
DESeq = 20.3
edgeR = 1.1
voom = 1
NBPSeq = 22
NOISeq = 3.1
baySeq = 1
Simes = 5.2
Union = 5.2
Intersection = 1.1
PANDORA = 1
Fisher = 2.8
Whitlock = 1.9
Algorithm
baySeq
DESeq
edgeR
Fisher
Intersection
NBPSeq
NOISeq
PANDORA
Simes
Union
voom
Whitlock

Figure S15: False Discovery Curves using each package's specific normalization and adjusted p-values

FDCs generated with simulated data for each statistical test and each p-value combination method supported by metaseqR for five organisms and two simulation configurations, using Benjamini-Hochberg adjusted p-values and each package specific normalization algorithm instead of EDASeq. The results for each organism and simulation configuration can be distinguished by the right and top side titles of each panel, referring to the organism from which simulation parameters are estimated and the simulation configuration, respectively. The performance value according to the AUFC is displayed in each panel next to the curves (Supplementary methods). The lowest possible value is 1, indicating no false discoveries among the first 500 top ranked genes according to statistical significance. Dashed lines represent individual tests whereas solid lines represent p-value combinations and the thicker solid line highlights the FDC produced by PANDORA. limma voom is constantly among the best performing algorithms and PANDORA among the top methods in general. As expected, the higher number of replicates increases the accuracy and performance of all algorithms (right panels). The curves as well as the AUFC values are constructed and calculated across ten simulations for each organism and simulation configuration, respectively. The performance trends are the same as when using unadjusted p-values.

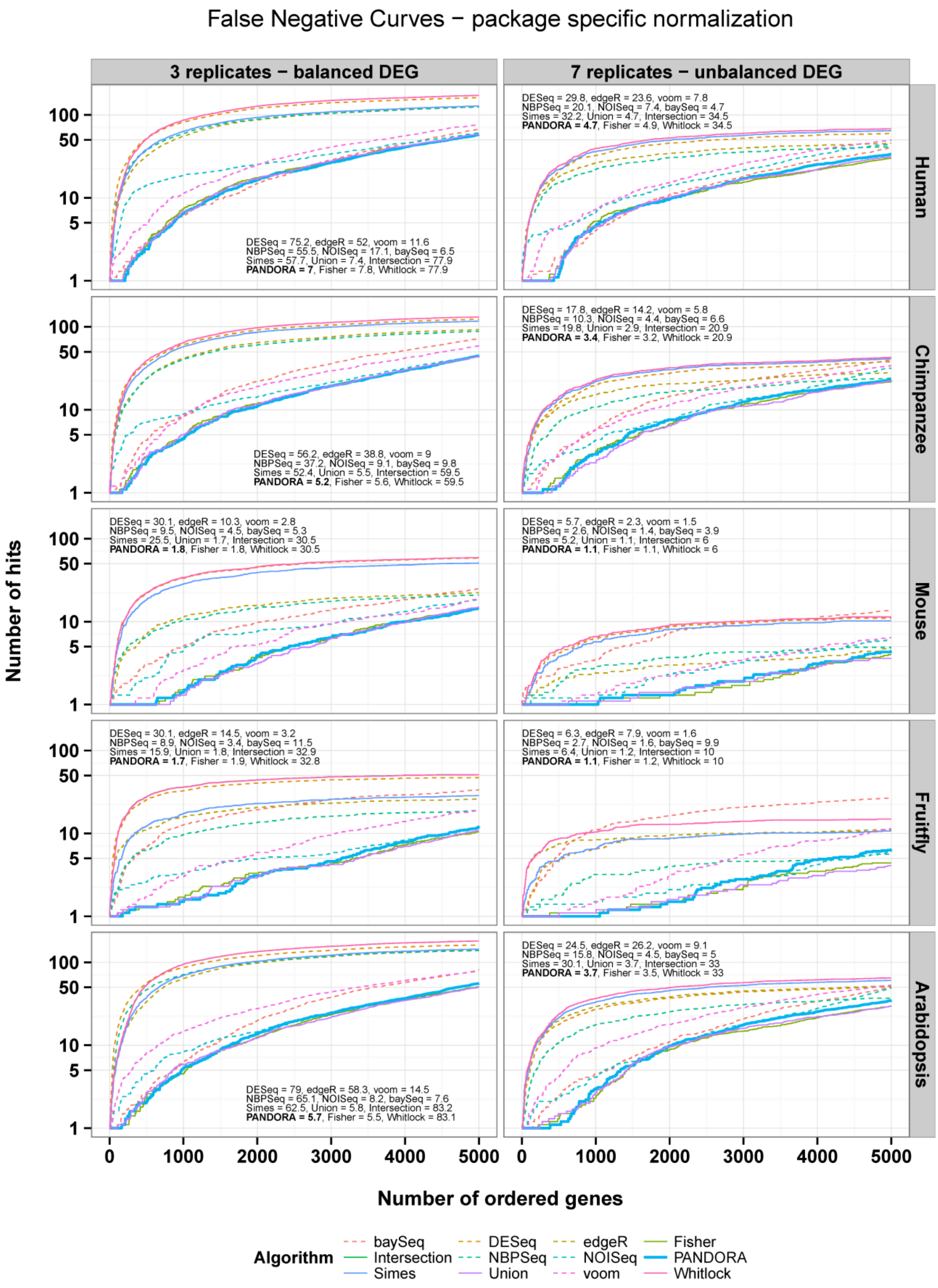
False Negative Curves − package specific normalization
3 replicates − balanced DEG
7 replicates − unbalanced DEG
Human
Chimpanzee
Mouse
Fruitfly
Arabidopsis
Number of hits
Number of ordered genes
DESeq = 75.2, edgeR = 52, voom = 11.6
NBPSeq = 55.5, NOISeq = 17.1, baySeq = 6.5
Simes = 57.7, Union = 7.4, Intersection = 77.9
PANDORA = 7, Fisher = 7.8, Whitlock = 77.9
DESeq = 29.8, edgeR = 23.6, voom = 7.8
NBPSeq = 20.1, NOISeq = 7.4, baySeq = 4.7
Simes = 32.2, Union = 4.7, Intersection = 34.5
PANDORA = 4.7, Fisher = 4.9, Whitlock = 34.5
DESeq = 56.2, edgeR = 38.8, voom = 9
NBPSeq = 37.2, NOISeq = 9.1, baySeq = 9.8
Simes = 52.4, Union = 5.5, Intersection = 59.5
PANDORA = 5.2, Fisher = 5.6, Whitlock = 59.5
DESeq = 17.8, edgeR = 14.2, voom = 5.8
NBPSeq = 10.3, NOISeq = 4.4, baySeq = 6.6
Simes = 19.8, Union = 2.9, Intersection = 20.9
PANDORA = 3.4, Fisher = 3.2, Whitlock = 20.9
DESeq = 30.1, edgeR = 10.3, voom = 2.8
NBPSeq = 9.5, NOISeq = 4.5, baySeq = 5.3
Simes = 25.5, Union = 1.7, Intersection = 30.5
PANDORA = 1.8, Fisher = 1.8, Whitlock = 30.5
DESeq = 5.7, edgeR = 2.3, voom = 1.5
NBPSeq = 2.6, NOISeq = 1.4, baySeq = 3.9
Simes = 5.2, Union = 1.1, Intersection = 6
PANDORA = 1.1, Fisher = 1.1, Whitlock = 6
DESeq = 30.1, edgeR = 14.5, voom = 3.2
NBPSeq = 8.9, NOISeq = 3.4, baySeq = 11.5
Simes = 15.9, Union = 1.8, Intersection = 32.9
PANDORA = 1.7, Fisher = 1.9, Whitlock = 32.8
DESeq = 6.3, edgeR = 7.9, voom = 1.6
NBPSeq = 2.7, NOISeq = 1.6, baySeq = 9.9
Simes = 6.4, Union = 1.2, Intersection = 10
PANDORA = 1.1, Fisher = 1.2, Whitlock = 10
DESeq = 79, edgeR = 58.3, voom = 14.5
NBPSeq = 65.1, NOISeq = 8.2, baySeq = 7.6
Simes = 62.5, Union = 5.8, Intersection = 83.2
PANDORA = 5.7, Fisher = 5.5, Whitlock = 83.1
DESeq = 24.5, edgeR = 26.2, voom = 9.1
NBPSeq = 15.8, NOISeq = 4.5, baySeq = 5
Simes = 30.1, Union = 3.7, Intersection = 33
PANDORA = 3.7, Fisher = 3.5, Whitlock = 33
Algorithm
baySeq
DESeq
edgeR
Fisher
Intersection
NBPSeq
NOISeq
PANDORA
Simes
Union
voom
Whitlock

Figure S16: False Negative Curves using each package's specific normalization and adjusted p-values

FNCs generated with simulated data for each statistical test and each p-value combination method supported by metaseqR for five organisms and two simulation configurations, using Benjamini-Hochberg adjusted p-values and each package specific normalization instead of EDASeq. The results for each organism and simulation configuration can be distinguished by the right and top side titles of each panel, referring to the organism from which simulation parameters are estimated and the simulation configuration respectively. The performance value according to the AUFC is displayed in each panel next to the curves (Supplementary methods). The lowest possible value is 1, indicating no false negative hits among the first 2000 ranked genes according to increasing statistical significance (from higher to lower p-values). Dashed lines represent individual tests whereas solid lines represent p-value combinations and the thicker solid line highlights the FNC produced by PANDORA. The performance of statistical tests where multiple testing correction can be applied drops considerably, with limma voom showing the most moderate drop. PANDORA is less affected. As expected, the higher number of replicates partially remedies the aforementioned performance drop. The curves as well as the AUFC values are constructed and calculated across ten simulations for each organism and simulation configuration, respectively.

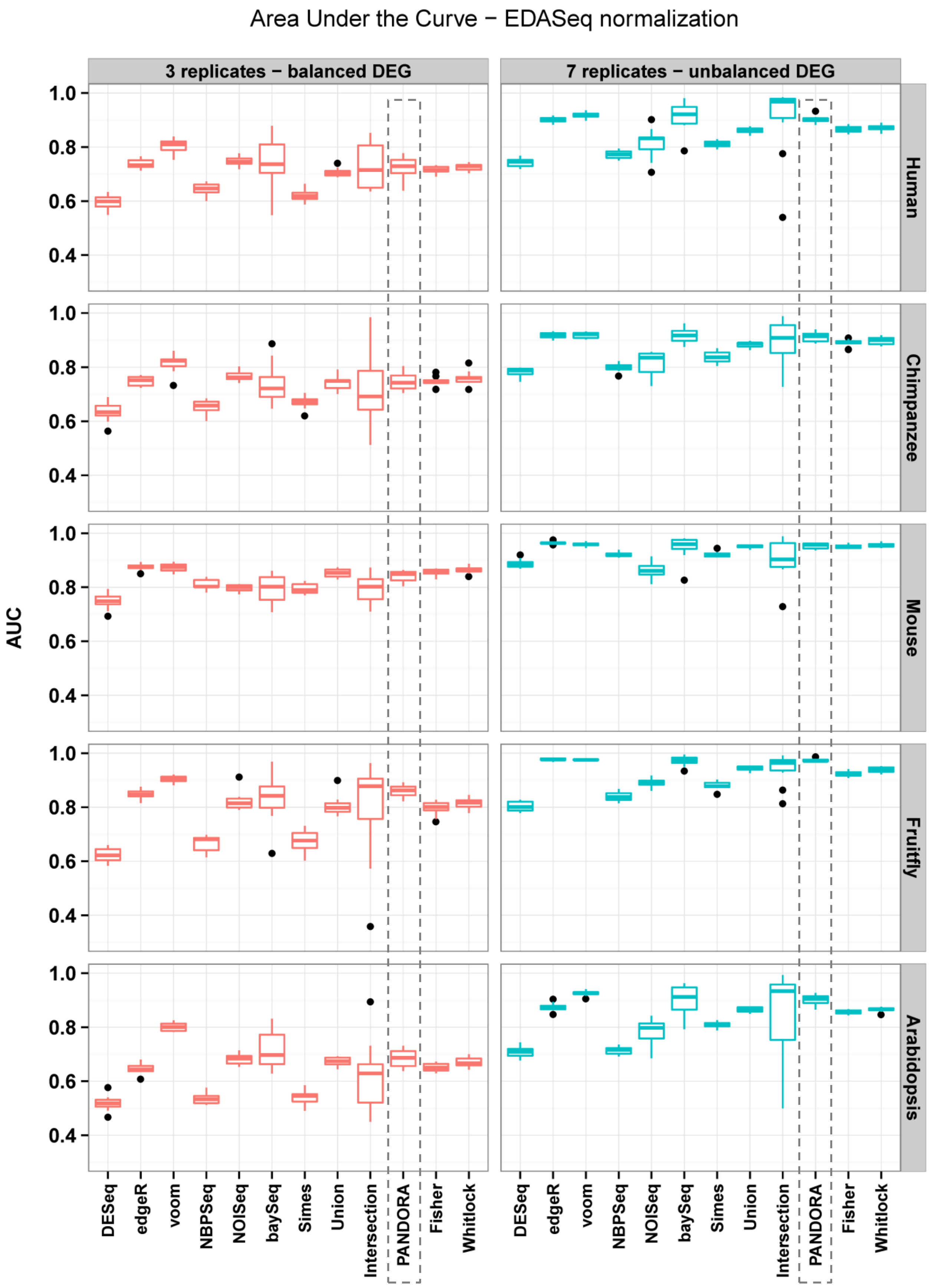

Area Under the Curve − EDASeq normalization
3 replicates − balanced DEG
7 replicates − unbalanced DEG
AUC
1.0
0.8
0.6
0.4
Human
Chimpanzee
Mouse
Fruitfly
Arabidopsis
DESeq
edgeR
voom
NBPSeq
NOISeq
baySeq
Simes
Union
Intersection
PANDORA
Fisher
Whitlock

Figure S17: Receiver Operating Characteristic analysis using EDASeq normalization and adjusted p-values

The boxplots depict the summarized areas under the ROC curve across ten simulations for each organism and for each simulation configuration, using EDASeq normalization. The results for each organism and simulation configuration can be distinguished by the right and top side titles of each panel, referring to the organism from which simulation parameters are estimated and the simulation configuration, respectively. The dashed rectangle highlights the PANDORA method results. Each statistical test's and each p-value combination method's performance trends are similar, apart from the Mouse case, where the performance is increased in both simulation configurations. The impact of adjusted p-values is evident on DESeq, edgeR, limma voom and NBPSeq, as their area under the curve is lower than the unadjusted case. Incorporating more replicates to the simulation remedies this problem in most cases. PANDORA performs very similar to the highest scoring algorithms, according to the area under the ROC curves.

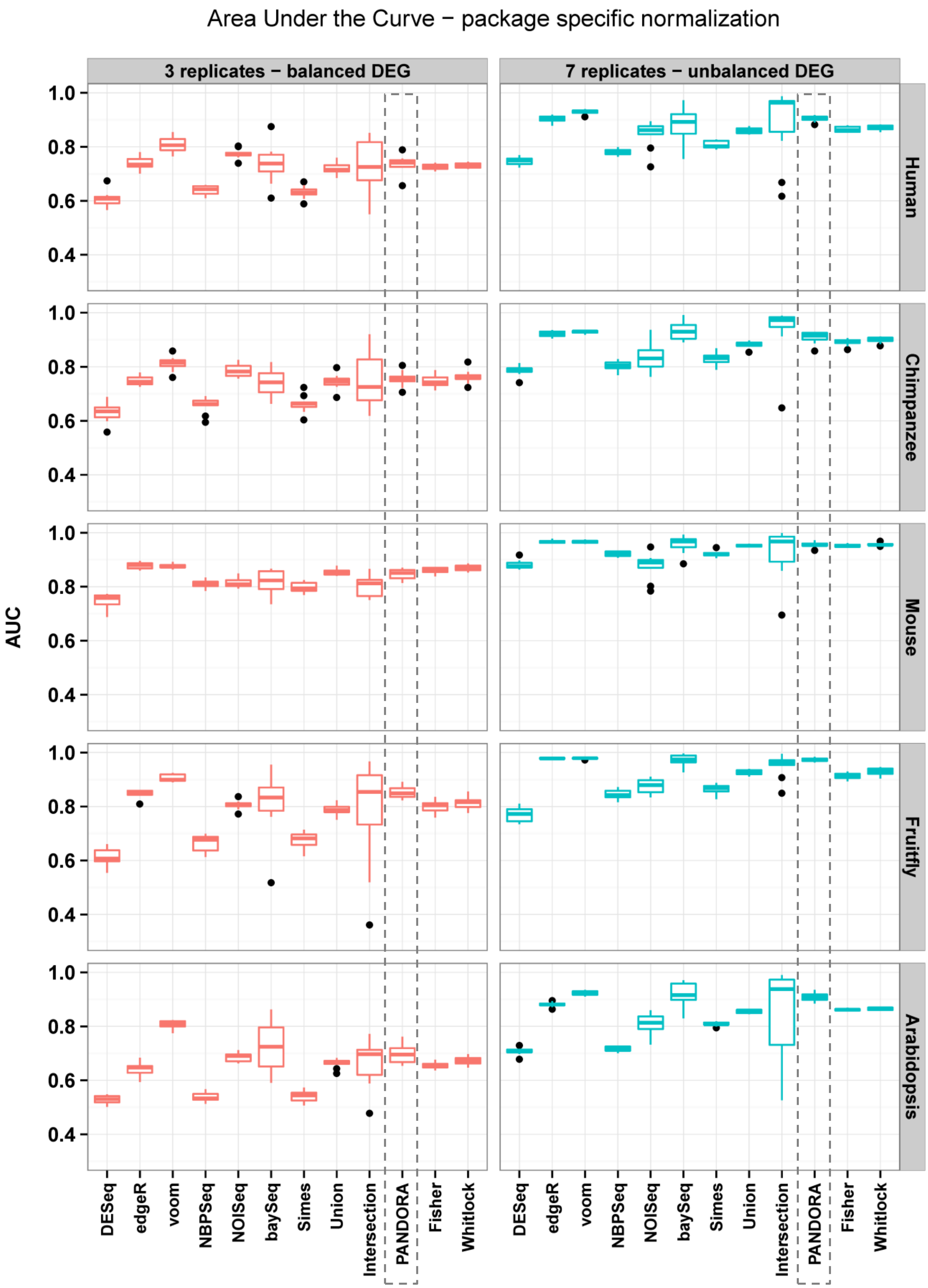

Area Under the Curve − package specific normalization
3 replicates − balanced DEG
7 replicates − unbalanced DEG
AUC
1.0
0.8
0.6
0.4
Human
Chimpanzee
Mouse
Fruitfly
Arabidopsis
DESeq
edgeR
voom
NBPSeq
NOISeq
baySeq
Simes
Union
Intersection
PANDORA
Fisher
Whitlock

Figure S18: Receiver Operating Characteristic analysis using each package's specific normalization and adjusted p-values

The boxplots depict the summarized areas under the ROC curve across ten simulations for each organism and for each simulation configuration, using each package's specific normalization instead of EDASeq. The results for each organism and simulation configuration can be distinguished by the right and top side titles of each panel, referring to the organism from which simulation parameters are estimated and the simulation configuration, respectively. The dashed rectangle highlights the PANDORA method results. Each statistical test's and each p-value combination method's performance trends are similar, apart from the Mouse case, where the performance is increased in both simulation configurations. The impact of adjusted p-values is evident on DESeq, edgeR, limma voom and NBPSeq, as their area under the curve is lower than the unadjusted case. Incorporating more replicates to the simulation remedies this problem in most cases. PANDORA performs very similar to the highest scoring algorithms, according to the area under the ROC curves. The performance trends do not change as compared to assessment with EDASeq normalization.

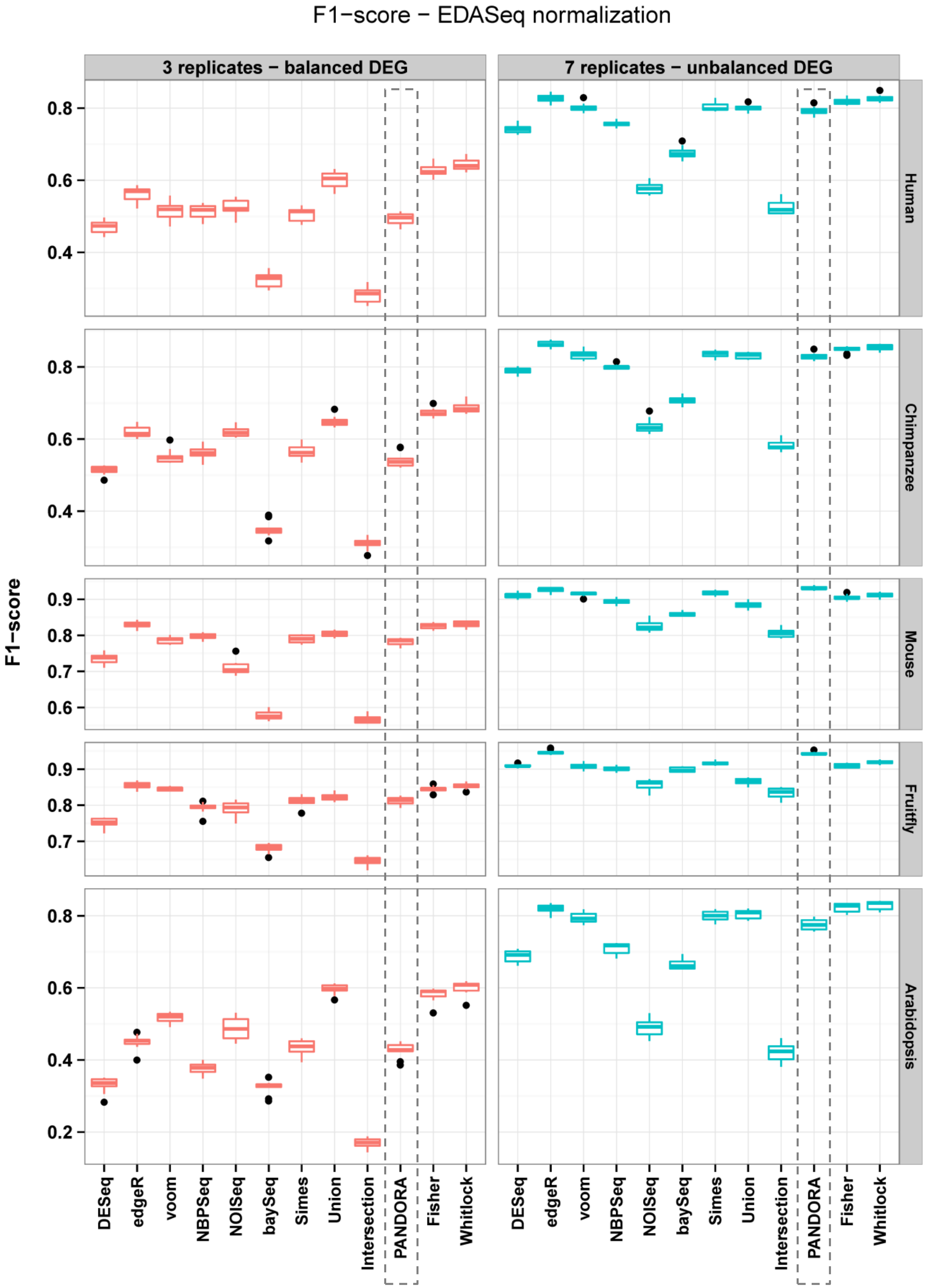

F1−score − EDASeq normalization
3 replicates − balanced DEG
7 replicates − unbalanced DEG
F1−score
Human
Chimpanzee
Mouse
Fruitfly
Arabidopsis
0.2
0.4
0.6
0.7
0.8
0.9
DESeq
edgeR
voom
NBPSeq
NOISeq
baySeq
Simes
Union
Intersection
PANDORA
Fisher
Whitlock

Figure S19: Analysis of the F_1-score using EDASeq normalization and adjusted p-values

The boxplots summarize the F_1-scores across ten simulations for each organism and for each simulation configuration using EDASeq normalization and Benjamini-Hochberg adjusted p-values. The F_1-scores for each organism and simulation configuration can be distinguished by the right and top side titles of each panel, referring to the organism from which simulation parameters are estimated and the simulation configuration respectively. The dashed rectangle highlights the PANDORA method results. PANDORA, although not prevailing, performs well in most cases.

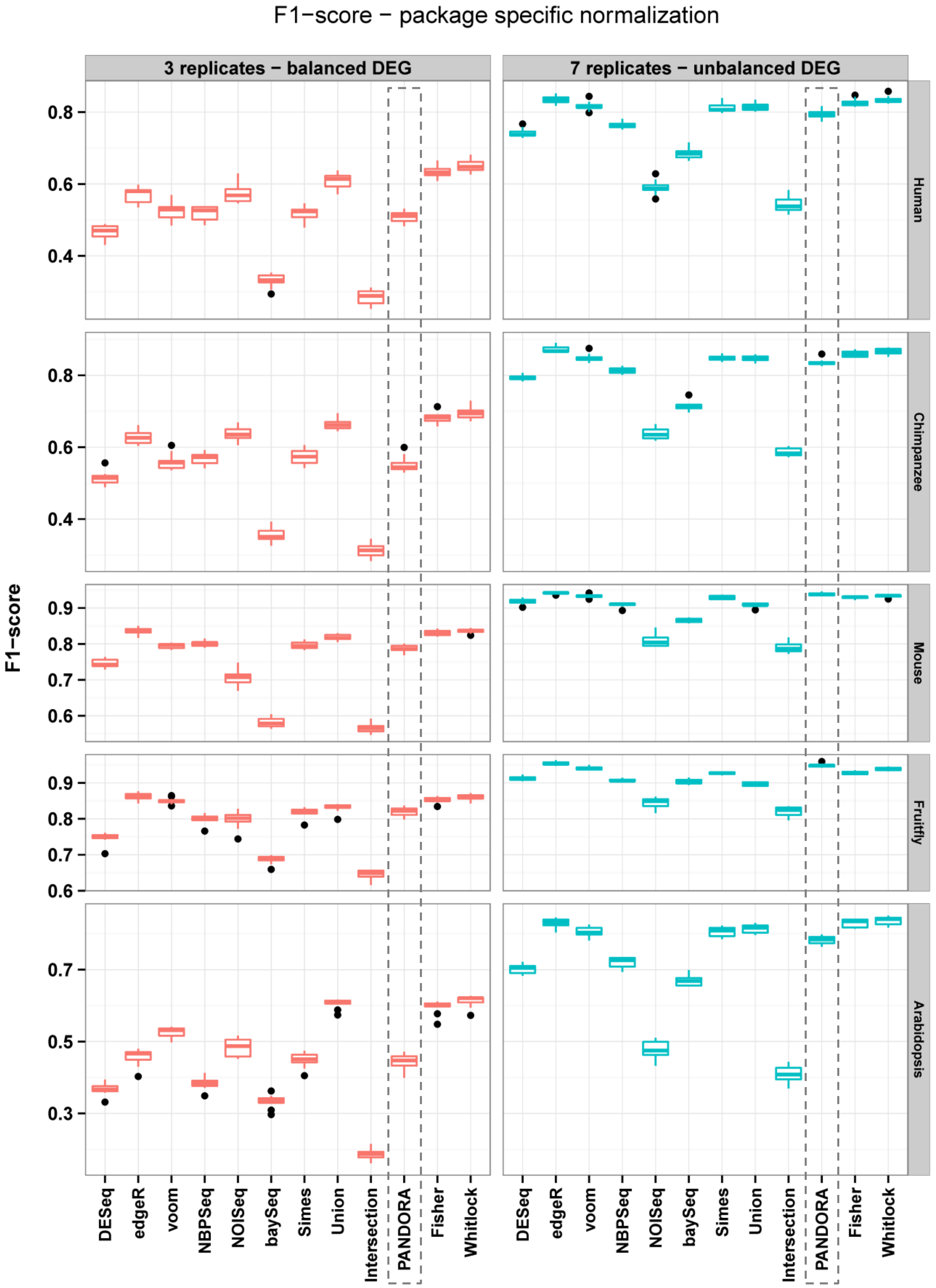
F1−score − package specific normalization
3 replicates − balanced DEG
7 replicates − unbalanced DEG
F1−score
Human
Chimpanzee
Mouse
Fruitfly
Arabidopsis
0.8
0.6
0.4
0.9
0.7
0.5
0.3
DESeq
edgeR
voom
NBPSeq
NOISeq
baySeq
Simes
Union
Intersection
PANDORA
Fisher
Whitlock

Figure S20: Analysis of the F_1-score using each package's specific normalization and adjusted p-values

The boxplots summarize the F_1-scores across ten simulations for each organism and for each simulation configuration, using each package's specific normalization instead of EDASeq and Benjamini-Hochberg adjusted p-values. The F_1-scores for each organism and simulation configuration can be distinguished by the right and top side titles of each panel, referring to the organism from which simulation parameters are estimated and the simulation configuration, respectively. The dashed rectangle highlights the PANDORA method results. PANDORA, although not prevailing, performs well in most cases.

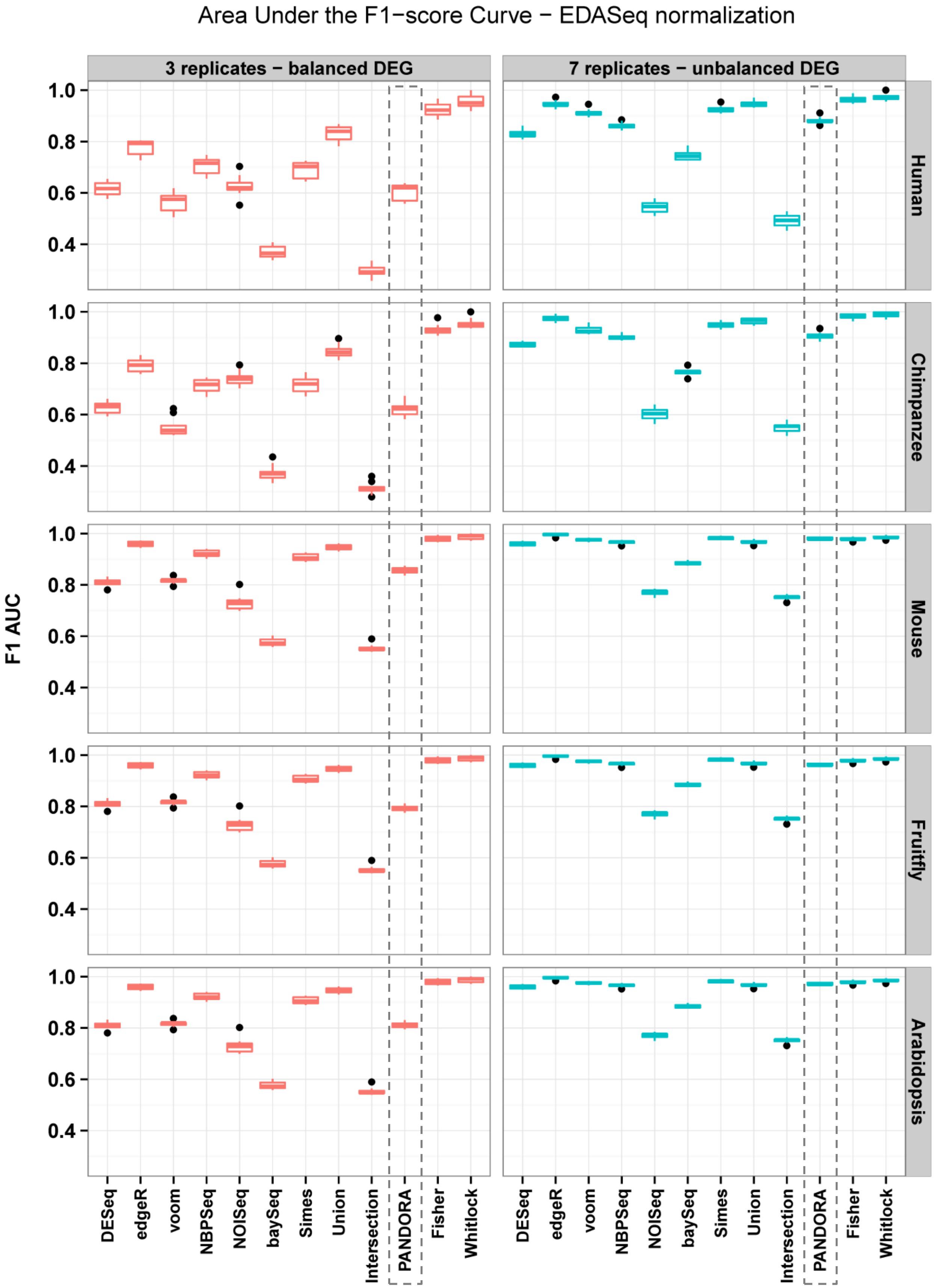
Area Under the F1−score Curve − EDASeq normalization
3 replicates − balanced DEG
7 replicates − unbalanced DEG
F1 AUC
1.0
0.8
0.6
0.4
Human
Chimpanzee
Mouse
Fruitfly
Arabidopsis
DESeq
edgeR
voom
NBPSeq
NOISeq
baySeq
Simes
Union
Intersection
PANDORA
Fisher
Whitlock

Figure S21: Areas under the F_1-score curves using EDASeq normalization and adjusted p-values

The boxplots depict the summarized areas under the F_1-scores curve across ten simulations for each organism and for each simulation configuration, using EDASeq normalization and Benjamini-Hochberg adjusted p-values. The results for each organism and simulation configuration can be distinguished by the right and top side titles of each panel, referring to the organism from which simulation parameters are estimated and the simulation configuration, respectively. The dashed rectangle highlights the PANDORA method results. PANDORA, although not prevalent, performs well in most cases, when looking at the top ranked genes (contributing to the construction of an F_1-scores curve).

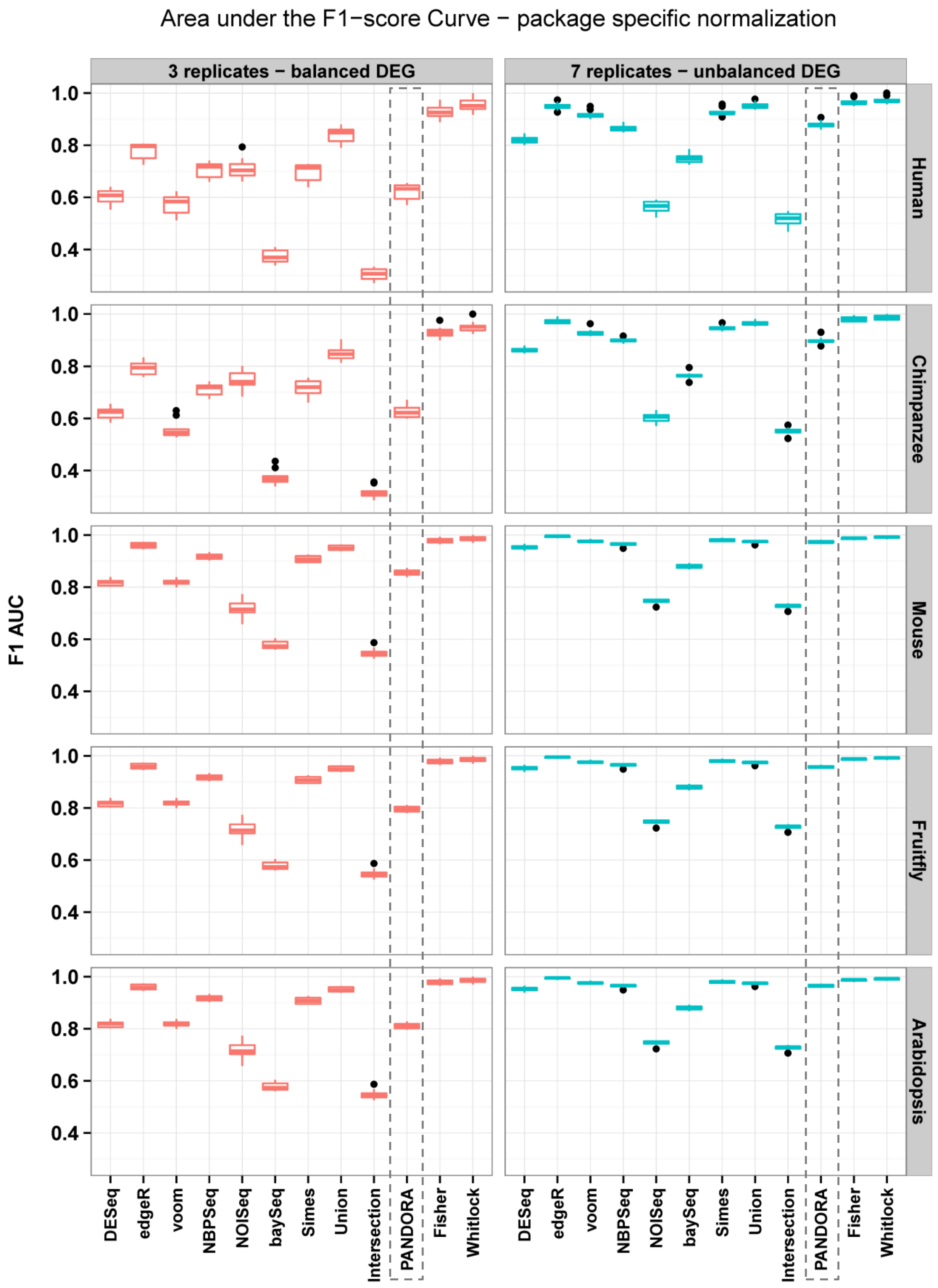

Area under the F1−score Curve − package specific normalization
3 replicates − balanced DEG
7 replicates − unbalanced DEG
F1 AUC
1.0
0.8
0.6
0.4
Human
Chimpanzee
Mouse
Fruitfly
Arabidopsis
DESeq
edgeR
voom
NBPSeq
NOISeq
baySeq
Simes
Union
Intersection
PANDORA
Fisher
Whitlock

Figure S22: Areas under the F_1-score curves using each package's specific normalization and adjusted p-values

The boxplots depict the summarized areas under the F_1-score curve across ten simulations for each organism and for each simulation configuration, using each package's specific normalization instead of EDASeq and Benjamini-Hochberg adjusted p-values. The results for each organism and simulation configuration can be distinguished by the right and top side titles of each panel, referring to the organism from which simulation parameters are estimated and the simulation configuration, respectively. The dashed rectangle highlights the PANDORA method results. PANDORA, although not prevalent, performs well in most cases, when looking at the top ranked genes (contributing to the construction of an F_1-score curve).

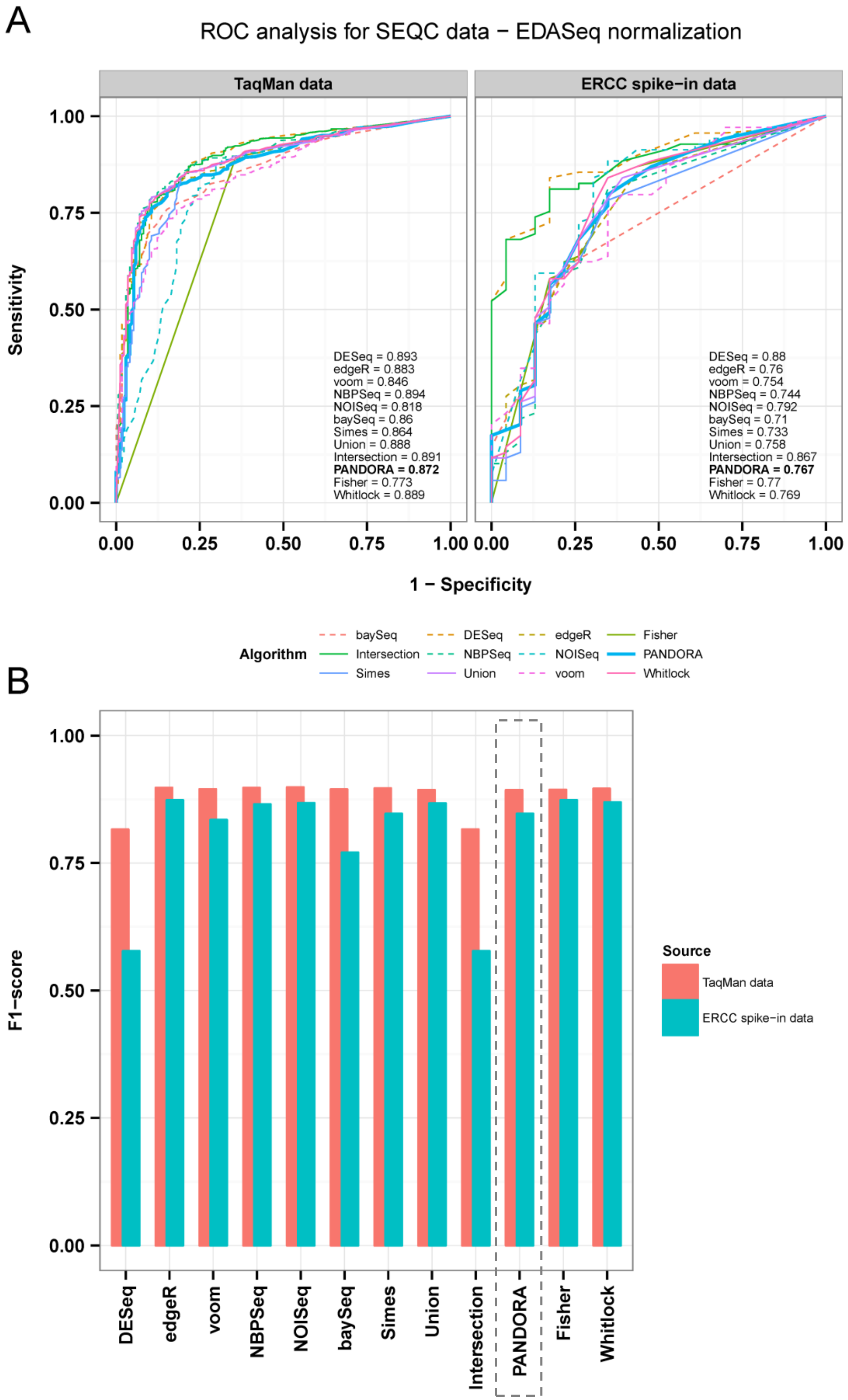
A
ROC analysis for SEQC data − EDASeq normalization
TaqMan data
ERCC spike−in data
Sensitivity
1 − Specificity
DESeq = 0.893
edgeR = 0.883
voom = 0.846
NBPSeq = 0.894
NOISeq = 0.818
baySeq = 0.86
Simes = 0.864
Union = 0.888
Intersection = 0.891
PANDORA = 0.872
Fisher = 0.773
Whitlock = 0.889
DESeq = 0.88
edgeR = 0.76
voom = 0.754
NBPSeq = 0.744
NOISeq = 0.792
baySeq = 0.71
Simes = 0.733
Union = 0.758
Intersection = 0.867
PANDORA = 0.767
Fisher = 0.77
Whitlock = 0.769
Algorithm
baySeq
DESeq
edgeR
Fisher
Intersection
NBPSeq
NOISeq
PANDORA
Simes
Union
voom
Whitlock
B
F1−score
Source
TaqMan data
ERCC spike−in data
DESeq
edgeR
voom
NBPSeq
NOISeq
baySeq
Simes
Union
Intersection
PANDORA
Fisher
Whitlock

Figure S23: Receiver Operating Characteristic and F_1-score analysis for SEQC data using EDASeq normalization and adjusted p-values

(A) ROC curves calculated using TaqMan data (left panel) and ERCC spike-in data (right panel) using EDASeq normalization for data processing and Benjamini-Hochberg corrected p-values. Dashed lines represent individual tests whereas solid lines represent p-value combinations and the thicker solid line highlights the ROC produced by PANDORA. Actual area under the curve values are shown on the right of each panel. In both cases DESeq performs slightly better than all the rest. In the case of TaqMan data, NBPSeq demonstrates similar performance with DESeq. The performance of PANDORA lies in the middle of area under the curve values in both cases, indicating good performance.

(B) F_1-scores using TaqMan data (red bars) and ERCC spike-in data (green bars) using EDASeq normalization for data processing and Benjamini-Hochberg corrected p-values. The dashed rectangle highlights the PANDORA method results. All methods apart from DESeq and Intersection achieve similar F_1-score levels when looking only at the TaqMan gene list (not the total putative DEG list of the SEQC data). When using ERCC spike-in data, baySeq and Simes also perform poorly. PANDORA, although not among the prevalent methods, performs well.

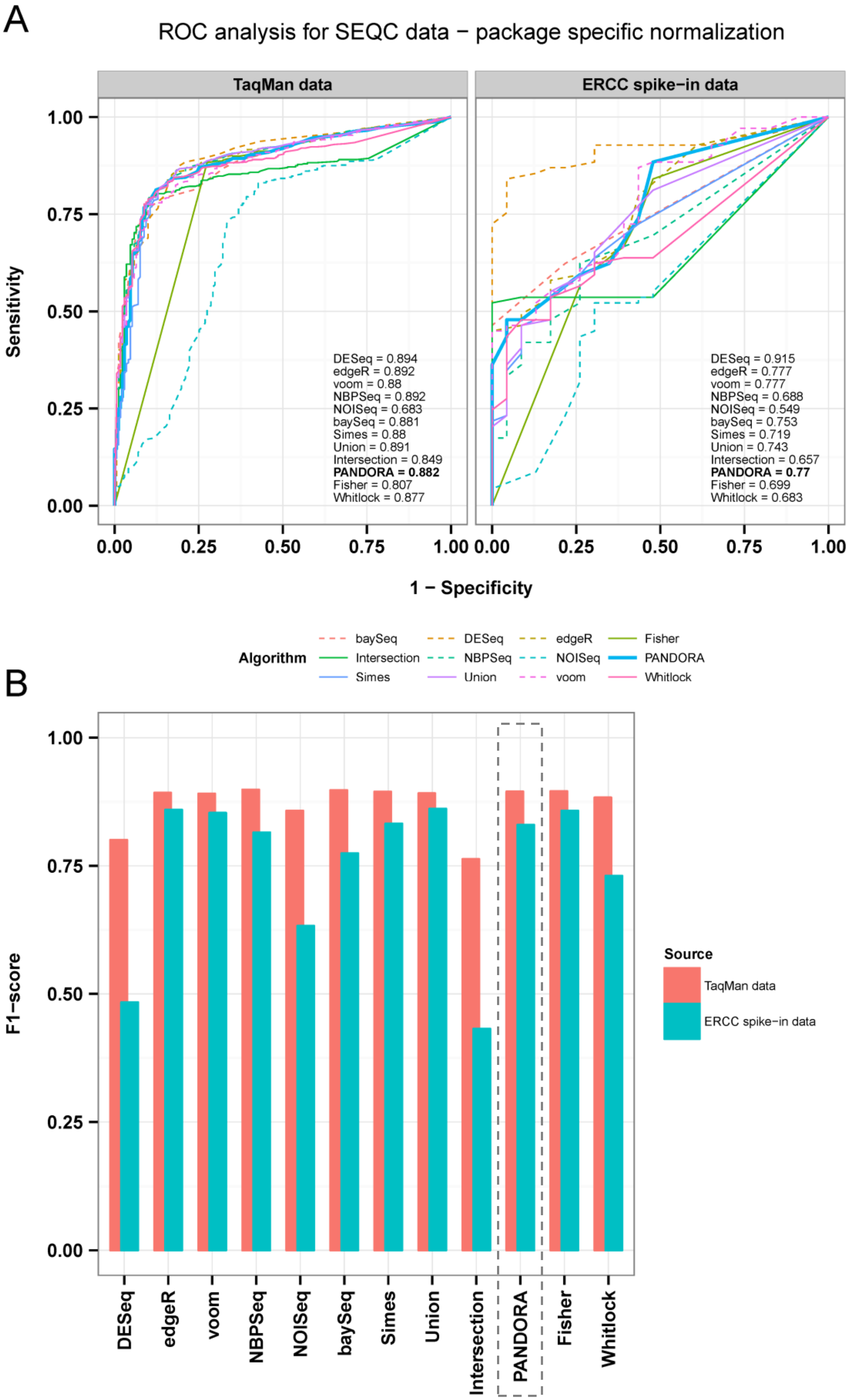
A
ROC analysis for SEQC data − package specific normalization
TaqMan data
ERCC spike−in data
Sensitivity
1 − Specificity
0.00
0.25
0.50
0.75
1.00
DESeq = 0.894
edgeR = 0.892
voom = 0.88
NBPSeq = 0.892
NOISeq = 0.683
baySeq = 0.881
Simes = 0.88
Union = 0.891
Intersection = 0.849
PANDORA = 0.882
Fisher = 0.807
Whitlock = 0.877
DESeq = 0.915
edgeR = 0.777
voom = 0.777
NBPSeq = 0.688
NOISeq = 0.549
baySeq = 0.753
Simes = 0.719
Union = 0.743
Intersection = 0.657
PANDORA = 0.77
Fisher = 0.699
Whitlock = 0.683
Algorithm
baySeq
DESeq
edgeR
Fisher
Intersection
NBPSeq
NOISeq
PANDORA
Simes
Union
voom
Whitlock
B
F1−score
Source
TaqMan data
ERCC spike−in data
DESeq
edgeR
voom
NBPSeq
NOISeq
baySeq
Simes
Union
Intersection
PANDORA
Fisher
Whitlock

Figure S24: Receiver Operating Characteristic and F_1-score analysis for SEQC data using each package's specific normalization and adjusted p-values

(A) ROC curves calculated using TaqMan data (left panel) and ERCC spike-in data (right panel) using each package's specific normalization for data processing instead of EDASeq and Benjamini-Hochberg corrected p-values. Actual area under the curve values are shown on the right of each panel. Dashed lines represent individual tests whereas solid lines represent p-value combinations and the thicker solid line highlights the ROC produced by PANDORA. In the cases of TaqMan data, DESeq performs slightly better than all the rest, whereas in the case of ERCC spike-in data, it performs considerably better. The performance of all other methods drops in the case of ERCC spike-ins, and particularly that of NOISeq and Fisher. The performance of PANDORA lies in the middle of area under the curve values in both cases, indicating good performance.

(B) F_1-scores using TaqMan data (red bars) and ERCC spike-in data (green bars) using EDASeq normalization for data processing and Benjamini-Hochberg corrected p-values. The dashed rectangle highlights the PANDORA method results. All methods apart from DESeq, NOISeq and Intersection achieve similar F_1-score levels when looking only at the TaqMan gene list (not the total putative DEG list of the SEQC data). When using ERCC spike-in data, baySeq, Simes and Whitlock also perform poorly. PANDORA is again in the middle in both cases, indicating good performance.

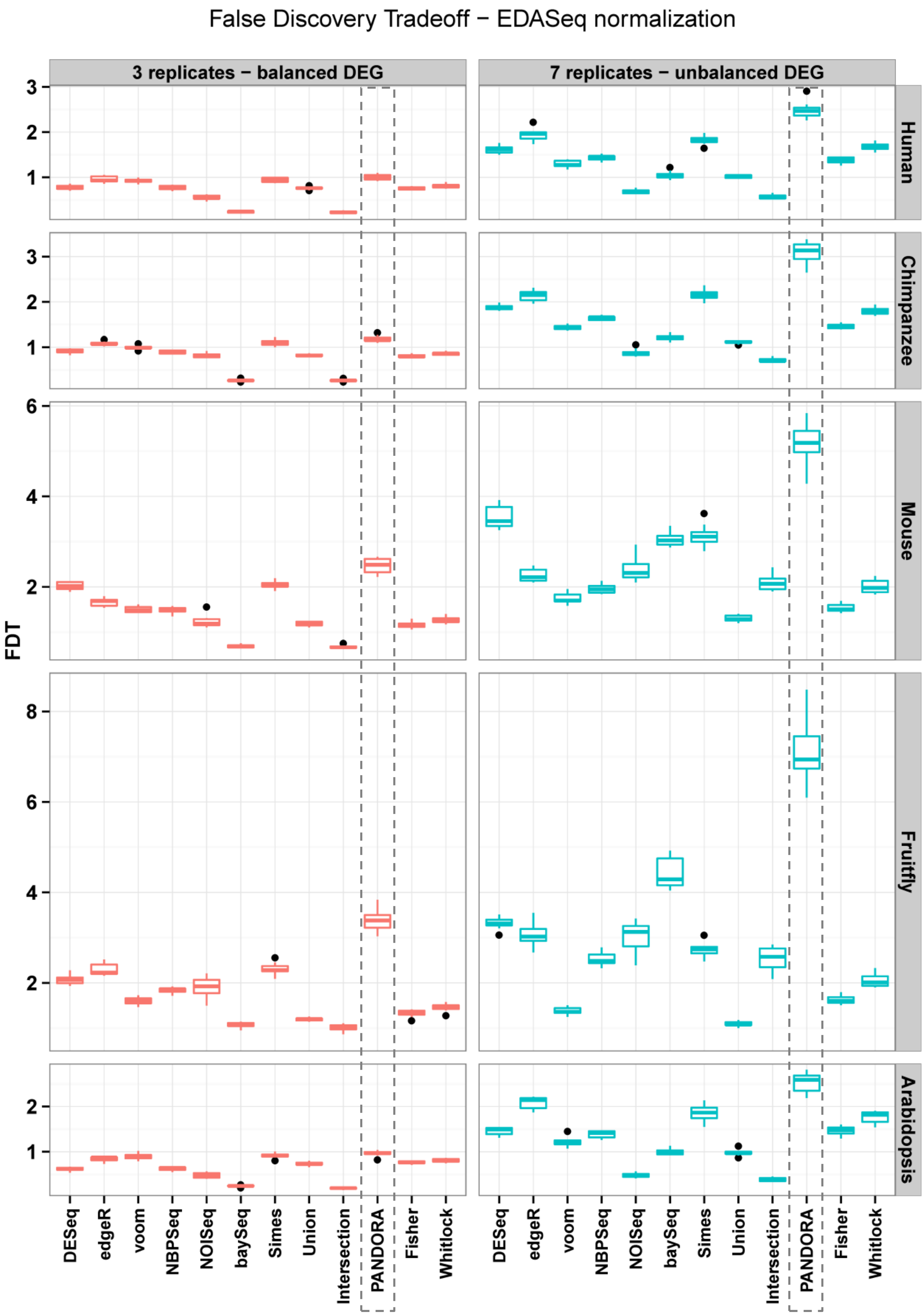

False Discovery Tradeoff − EDASeq normalization
3 replicates − balanced DEG
7 replicates − unbalanced DEG
FDT
Human
Chimpanzee
Mouse
Fruitfly
Arabidopsis
DESeq
edgeR
voom
NBPSeq
NOISeq
baySeq
Simes
Union
Intersection
PANDORA
Fisher
Whitlock

Figure S25: Analysis of the False Discovery Tradeoff using EDASeq normalization and unadjusted p-values

The boxplots summarize the FDTs across ten simulations for each organism and for each simulation configuration. The FDTs for each organism and simulation configuration can be distinguished by the right and top side titles of each panel, referring to the organism from which simulation parameters are estimated and the simulation configuration respectively. The dashed rectangle highlights the PANDORA method results. The prevalence of PANDORA is evident in all cases. Intersection and baySeq constantly show the lowest FDT. FDTs are calculated using the final gene lists returned by each method at a p-value cutoff of 0.05.

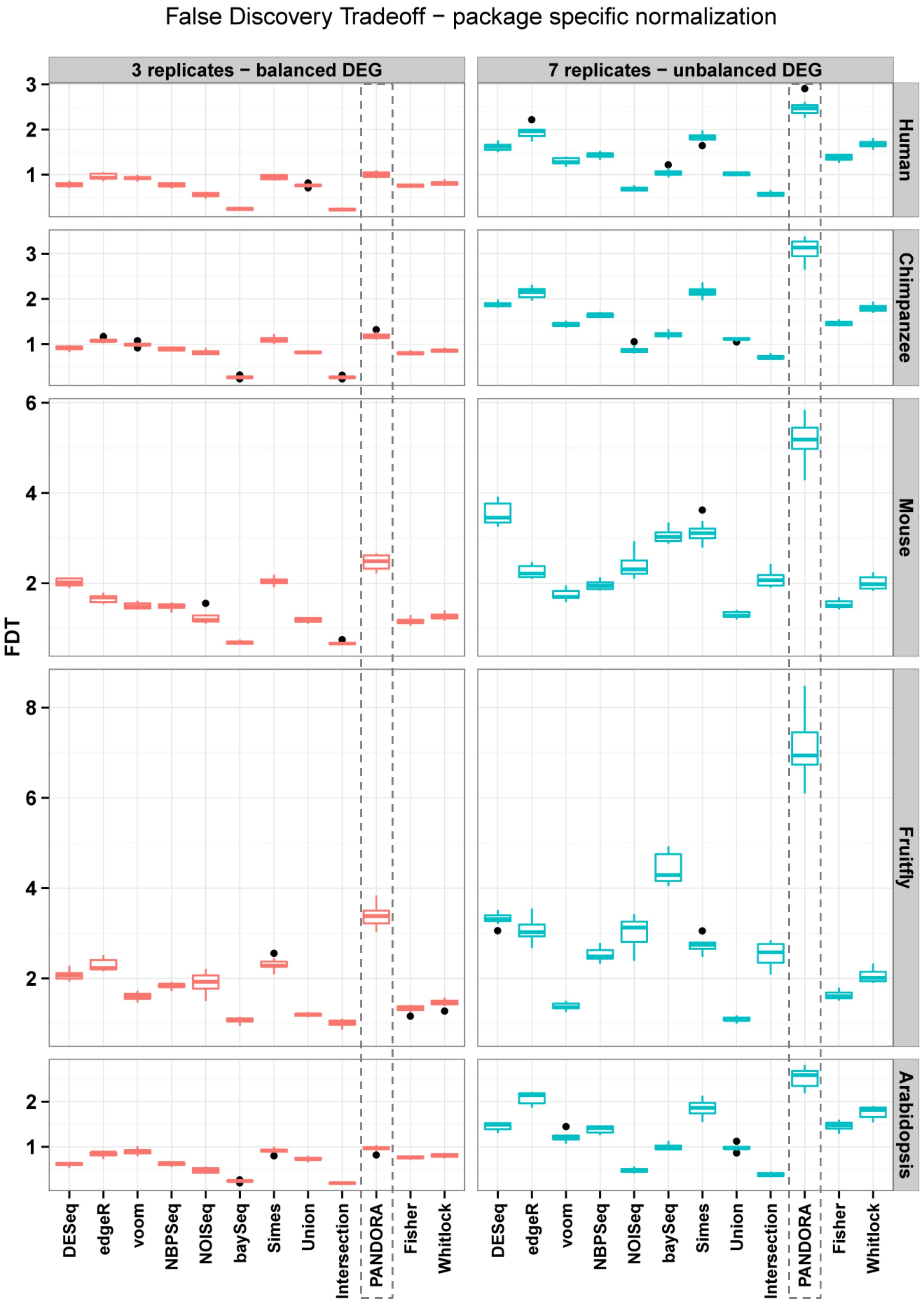

False Discovery Tradeoff − package specific normalization
3 replicates − balanced DEG
7 replicates − unbalanced DEG
Human
Chimpanzee
Mouse
Fruitfly
Arabidopsis
FDT
DESeq
edgeR
voom
NBPSeq
NOISeq
baySeq
Simes
Union
Intersection
PANDORA
Fisher
Whitlock

Figure S26: Analysis of the False Discovery Tradeoff using each package's specific normalization and unadjusted p-values

The boxplots summarize the FDTs across ten simulations for each organism and for each simulation configuration, when using each package's specific normalization instead of EDASeq. The FDTs for each organism and simulation configuration can be distinguished by the right and top side titles of each panel, referring to the organism from which simulation parameters are estimated and the simulation configuration respectively. The dashed rectangle highlights the PANDORA method results. The prevalence of PANDORA is evident in all cases. Intersection and baySeq constantly show the lowest FDT. FDTs are calculated using the final gene lists returned by each method at a p-value cutoff of 0.05. The general performance trends do not change when compared to the case of using EDASeq normalization.

4. Supplementary tables

Table S1 Numbers of statistically significant, TPs, FNs and FPs for each metaseqR method over ten simulations

		3 replicates – balanced DEG				7 replicates – unbalanced DEG			
		Significant	TP	FN	FP	Significant	TP	FN	FP
**Human**	**DESeq**	1091 +/- 20	838 +/- 11	162 +/- 11	253 +/- 14	1437 +/- 19	1155 +/- 4	45 +/- 4	282 +/- 20
	**edgeR**	1333 +/- 13	897 +/- 9	103 +/- 9	436 +/- 17	1651 +/- 31	1166 +/- 6	34 +/- 6	485 +/- 31
	**voom**	1362 +/- 19	886 +/- 8	114 +/- 8	476 +/- 20	1778 +/- 38	1157 +/- 6	43 +/- 6	622 +/- 39
	**NBPSeq**	1406 +/- 15	899 +/- 9	101 +/- 9	508 +/- 18	1750 +/- 28	1175 +/- 4	25 +/- 4	575 +/- 29
	**NOISeq**	646 +/- 44	585 +/- 32	415 +/- 32	61 +/- 15	879 +/- 26	858 +/- 26	342 +/- 26	21 +/- 7
	**baySeq**	410 +/- 12	408 +/- 12	592 +/- 12	3 +/- 1	907 +/- 10	905 +/- 10	295 +/- 10	2 +/- 2
	**Simes**	1134 +/- 14	857 +/- 10	143 +/- 10	276 +/- 10	1492 +/- 21	1160 +/- 5	40 +/- 5	332 +/- 23
	**Union**	1624 +/- 29	922 +/- 7	78 +/- 7	702 +/- 30	2068 +/- 45	1180 +/- 3	20 +/- 3	889 +/- 46
	**Intersection**	405 +/- 12	402 +/- 12	597 +/- 12	3 +/- 1	813 +/- 21	812 +/- 21	388 +/- 21	1 +/- 1
	**PANDORA**	936 +/- 23	806 +/- 16	194 +/- 16	130 +/- 12	1265 +/- 19	1129 +/- 9	71 +/- 9	137 +/- 16
	**Weight-X**	1286 +/- 21	882 +/- 10	118 +/- 10	403 +/- 19	1677 +/- 35	1160 +/- 5	40 +/- 5	517 +/- 38
	**Fisher**	1656 +/- 47	927 +/- 7	73 +/- 8	729 +/- 46	1924 +/- 48	1177 +/- 4	23 +/- 4	747 +/- 47
	**Whitlock**	1563 +/- 36	920 +/- 7	80 +/- 7	643 +/- 37	1729 +/- 43	1171 +/- 5	29 +/- 5	558 +/- 43
**Chimpanzee**	**DESeq**	1091 +/- 20	838 +/- 11	162 +/- 11	253 +/- 14	1437 +/- 19	1155 +/- 4	45 +/- 4	282 +/- 20
	**edgeR**	1333 +/- 13	897 +/- 9	103 +/- 9	436 +/- 17	1651 +/- 31	1166 +/- 6	34 +/- 6	485 +/- 31
	**Voom**	1362 +/- 19	886 +/- 8	114 +/- 8	476 +/- 20	1778 +/- 38	1157 +/- 6	43 +/- 6	622 +/- 39
	**NBPSeq**	1406 +/- 15	899 +/- 9	101 +/- 9	508 +/- 18	1750 +/- 28	1175 +/- 4	25 +/- 4	575 +/- 29
	**NOISeq**	646 +/- 44	585 +/- 32	415 +/- 32	61 +/- 15	879 +/- 26	858 +/- 26	342 +/- 26	21 +/- 7
	**baySeq**	410 +/- 12	408 +/- 12	592 +/- 12	3 +/- 1	907 +/- 10	905 +/- 10	295 +/- 10	2 +/- 2
	**Simes**	1134 +/- 14	857 +/- 10	143 +/- 10	276 +/- 10	1492 +/- 21	1160 +/- 5	40 +/- 5	332 +/- 23
	**Union**	1624 +/- 29	922 +/- 7	78 +/- 7	702 +/- 30	2068 +/- 45	1180 +/- 3	20 +/- 3	889 +/- 46
	**Intersection**	405 +/- 12	402 +/- 12	597 +/- 12	3 +/- 1	813 +/- 21	812 +/- 21	388 +/- 21	1 +/- 1
	**PANDORA**	930 +/- 23	804 +/- 17	196 +/- 17	126 +/- 13	1261 +/- 19	1128 +/- 9	72 +/- 9	133 +/- 16
	**Weight-X**	1286 +/- 21	882 +/- 10	118 +/- 10	403 +/- 19	1677 +/- 35	1160 +/- 5	40 +/- 5	517 +/- 38
	**Fisher**	1656 +/- 47	927 +/- 7	73 +/- 8	729 +/- 46	1924 +/- 48	1177 +/- 4	23 +/- 4	747 +/- 47
	**Whitlock**	1563 +/- 36	920 +/- 7	80 +/- 7	643 +/- 37	1729 +/- 43	1171 +/- 5	29 +/- 5	558 +/- 43
**Mouse**	**DESeq**	1091 +/- 20	838 +/- 11	162 +/- 11	253 +/- 14	1437 +/- 19	1155 +/- 4	45 +/- 4	282 +/- 20
	**edgeR**	1333 +/- 13	897 +/- 9	103 +/- 9	436 +/- 17	1651 +/- 31	1166 +/- 6	34 +/- 6	485 +/- 31
	**Voom**	1362 +/- 19	886 +/- 8	114 +/- 8	476 +/- 20	1778 +/- 38	1157 +/- 6	43 +/- 6	622 +/- 39
	**NBPSeq**	1406 +/- 15	899 +/- 9	101 +/- 9	508 +/- 18	1750 +/- 28	1175 +/- 4	25 +/- 4	575 +/- 29
	**NOISeq**	646 +/- 44	585 +/- 32	415 +/- 32	61 +/- 15	879 +/- 26	858 +/- 26	342 +/- 26	21 +/- 7
	**baySeq**	410 +/- 12	408 +/- 12	592 +/- 12	3 +/- 1	907 +/- 10	905 +/- 10	295 +/- 10	2 +/- 2
	**Simes**	1134 +/- 14	857 +/- 10	143 +/- 10	276 +/- 10	1492 +/- 21	1160 +/- 5	40 +/- 5	332 +/- 23
	**Union**	1624 +/- 29	922 +/- 7	78 +/- 7	702 +/- 30	2068 +/- 45	1180 +/- 3	20 +/- 3	889 +/- 46
	**Intersection**	405 +/- 12	402 +/- 12	597 +/- 12	3 +/- 1	813 +/- 21	812 +/- 21	388 +/- 21	1 +/- 1
	**PANDORA**	964 +/- 21	816 +/- 15	184 +/- 15	148 +/- 13	1288 +/- 16	1134 +/- 7	66 +/- 7	154 +/- 16
	**Weight-X**	1286 +/- 21	882 +/- 10	118 +/- 10	403 +/- 19	1677 +/- 35	1160 +/- 5	40 +/- 5	517 +/- 38
	**Fisher**	1656 +/- 47	927 +/- 7	73 +/- 8	729 +/- 46	1924 +/- 48	1177 +/- 4	23 +/- 4	747 +/- 47
	**Whitlock**	1563 +/- 36	920 +/- 7	80 +/- 7	643 +/- 37	1729 +/- 43	1171 +/- 5	29 +/- 5	558 +/- 43
**Fruitfly**	**DESeq**	1091 +/- 20	838 +/- 11	162 +/- 11	253 +/- 14	1437 +/- 19	1155 +/- 4	45 +/- 4	282 +/- 20
	**edgeR**	1333 +/- 13	897 +/- 9	103 +/- 9	436 +/- 17	1651 +/- 31	1166 +/- 6	34 +/- 6	485 +/- 31
	**Voom**	1362 +/- 19	886 +/- 8	114 +/- 8	476 +/- 20	1778 +/- 38	1157 +/- 6	43 +/- 6	622 +/- 39
	**NBPSeq**	1406 +/- 15	899 +/- 9	101 +/- 9	508 +/- 18	1750 +/- 28	1175 +/- 4	25 +/- 4	575 +/- 29
	**NOISeq**	646 +/- 44	585 +/- 32	415 +/- 32	61 +/- 15	879 +/- 26	858 +/- 26	342 +/- 26	21 +/- 7
	**baySeq**	410 +/- 12	408 +/- 12	592 +/- 12	3 +/- 1	907 +/- 10	905 +/- 10	295 +/- 10	2 +/- 2
	**Simes**	1134 +/- 14	857 +/- 10	143 +/- 10	276 +/- 10	1492 +/- 21	1160 +/- 5	40 +/- 5	332 +/- 23
	**Union**	1624 +/- 29	922 +/- 7	78 +/- 7	702 +/- 30	2068 +/- 45	1180 +/- 3	20 +/- 3	889 +/- 46
	**Intersection**	405 +/- 12	402 +/- 12	597 +/- 12	3 +/- 1	813 +/- 21	812 +/- 21	388 +/- 21	1 +/- 1
	**PANDORA**	842 +/- 13	764 +/- 12	236 +/- 12	78 +/- 7	1190 +/- 20	1105 +/- 12	95 +/- 12	84 +/- 13
	**Weight-X**	1286 +/- 21	882 +/- 10	118 +/- 10	403 +/- 19	1677 +/- 35	1160 +/- 5	40 +/- 5	517 +/- 38
	**Fisher**	1656 +/- 47	927 +/- 7	73 +/- 8	729 +/- 46	1924 +/- 48	1177 +/- 4	23 +/- 4	747 +/- 47
	**Whitlock**	1563 +/- 36	920 +/- 7	80 +/- 7	643 +/- 37	1729 +/- 43	1171 +/- 5	29 +/- 5	558 +/- 43
**Arabidopsis**	**DESeq**	1091 +/- 20	838 +/- 11	162 +/- 11	253 +/- 14	1437 +/- 19	1155 +/- 4	45 +/- 4	282 +/- 20
	**edgeR**	1333 +/- 13	897 +/- 9	103 +/- 9	436 +/- 17	1651 +/- 31	1166 +/- 6	34 +/- 6	485 +/- 31
	**Voom**	1362 +/- 19	886 +/- 8	114 +/- 8	476 +/- 20	1778 +/- 38	1157 +/- 6	43 +/- 6	622 +/- 39
	**NBPSeq**	1406 +/- 15	899 +/- 9	101 +/- 9	508 +/- 18	1750 +/- 28	1175 +/- 4	25 +/- 4	575 +/- 29
	**NOISeq**	646 +/- 44	585 +/- 32	415 +/- 32	61 +/- 15	879 +/- 26	858 +/- 26	342 +/- 26	21 +/- 7
	**baySeq**	410 +/- 12	408 +/- 12	592 +/- 12	3 +/- 1	907 +/- 10	905 +/- 10	295 +/- 10	2 +/- 2
	**Simes**	1134 +/- 14	857 +/- 10	143 +/- 10	276 +/- 10	1492 +/- 21	1160 +/- 5	40 +/- 5	332 +/- 23
	**Union**	1624 +/- 29	922 +/- 7	78 +/- 7	702 +/- 30	2068 +/- 45	1180 +/- 3	20 +/- 3	889 +/- 46
	**Intersection**	405 +/- 12	402 +/- 12	597 +/- 12	3 +/- 1	813 +/- 21	812 +/- 21	388 +/- 21	1 +/- 1
	**PANDORA**	914 +/- 19	797 +/- 14	202 +/- 13	117 +/- 12	1254 +/- 21	1124 +/- 10	76 +/- 10	130 +/- 16
	**Weight-X**	1286 +/- 21	882 +/- 10	118 +/- 10	403 +/- 19	1677 +/- 35	1160 +/- 5	40 +/- 5	517 +/- 38
	**Fisher**	1656 +/- 47	927 +/- 7	73 +/- 8	729 +/- 46	1924 +/- 48	1177 +/- 4	23 +/- 4	747 +/- 47
	**Whitlock**	1563 +/- 36	920 +/- 7	80 +/- 7	643 +/- 37	1729 +/- 43	1171 +/- 5	29 +/- 5	558 +/- 43

Table S2 False Discovery Rates (Benjamini-Hochberg adjusted p-values) from three "same vs same" comparisons (package specific normalization)

	DESeq	edgeR	voom	NBPSeq	baySeq	Simes	Union	Intersection	Weight AUFC	Weight X	Fisher	Whitlock AUFC
**SEQC group A**	0.0224	0.0006	<0.0001	0.0233	<0.0001	0.0194	0.0259	<0.0001	0.0004	<0.0001	0.0426	0.0260
**SEQC group B**	0.0003	0.0005	0.0008	0.0007	0.0001	0.0007	0.0008	<0.0001	0.0001	0.0005	0.0237	0.0049
**Brawand data**	0.0029	0.0002	<0.0001	0.0031	<0.0001	0.0021	0.0040	<0.0001	<0.0001	<0.0001	0.0282	0.0193

5. References

1. Gentleman, R.C., Carey, V.J., Bates, D.M., Bolstad, B., Dettling, M., Dudoit, S., Ellis, B., Gautier, L., Ge, Y., Gentry, J. *et al.* (2004) Bioconductor: open software development for computational biology and bioinformatics. *Genome Biol*, **5**, R80.
2. Robinson, M.D. and Oshlack, A. (2010) A scaling normalization method for differential expression analysis of RNA-seq data. *Genome Biol*, **11**, R25.
3. Dillies, M.A., Rau, A., Aubert, J., Hennequet-Antier, C., Jeanmougin, M., Servant, N., Keime, C., Marot, G., Castel, D., Estelle, J. *et al.* (2013) A comprehensive evaluation of normalization methods for Illumina high-throughput RNA sequencing data analysis. *Brief Bioinform*, **14**, 671-683.
4. Soneson, C. and Delorenzi, M. (2013) A comparison of methods for differential expression analysis of RNA-seq data. *BMC Bioinformatics*, **14**, 91.
5. Law, C.W., Chen, Y., Shi, W. and Smyth, G.K. (2014) Voom: precision weights unlock linear model analysis tools for RNA-seq read counts. *Genome Biol*, **15**, R29.
6. Rapaport, F., Khanin, R., Liang, Y., Pirun, M., Krek, A., Zumbo, P., Mason, C.E., Socci, N.D. and Betel, D. (2013) Comprehensive evaluation of differential gene expression analysis methods for RNA-seq data. *Genome Biol*, **14**, R95.
7. Mokry, M., Hatzis, P., Schuijers, J., Lansu, N., Ruzius, F.P., Clevers, H. and Cuppen, E. (2012) Integrated genome-wide analysis of transcription factor occupancy, RNA polymerase II binding and steady-state RNA levels identify differentially regulated functional gene classes. *Nucleic Acids Res*, **40**, 148-158.
8. Risso, D., Schwartz, K., Sherlock, G. and Dudoit, S. (2011) GC-content normalization for RNA-Seq data. *BMC Bioinformatics*, **12**, 480.
9. Robinson, M.D., McCarthy, D.J. and Smyth, G.K. (2010) edgeR: a Bioconductor package for differential expression analysis of digital gene expression data. *Bioinformatics*, **26**, 139-140.
10. Tarazona, S., Garcia-Alcalde, F., Dopazo, J., Ferrer, A. and Conesa, A. (2011) Differential expression in RNA-seq: a matter of depth. *Genome Res*, **21**, 2213-2223.
11. Yanming, D., W, S.D., S, C.J. and H, C.J. (2011) The NBP Negative Binomial Model for Assessing Differential Gene Expression from RNA-Seq. *Statistical Applications in Genetics and Molecular Biology*, **10**, 1-28.
12. Anders, S. and Huber, W. (2010) Differential expression analysis for sequence count data. *Genome Biol*, **11**, R106.
13. Hardcastle, T.J. and Kelly, K.A. (2010) baySeq: empirical Bayesian methods for identifying differential expression in sequence count data. *BMC Bioinformatics*, **11**, 422.
14. Simes, R.J. (1986) An improved Bonferroni procedure for multiple tests of significance. *Biometrika*, **73**, 751-754.
15. Fisher, R.A. (1932) *Statistical Methods for Research Workers*. Oliver and Boyd, Edinburgh.
16. Whitlock, M.C. (2005) Combining probability from independent tests: the weighted Z-method is superior to Fisher's approach. *J Evol Biol*, **18**, 1368-1373.
